# Combining Data-Driven and Structure-Based Approaches in Designing Dual PARP1-BRD4 Inhibitors for Breast Cancer Treatment

**DOI:** 10.1021/acs.jcim.4c01421

**Published:** 2024-09-18

**Authors:** Bo Feng, Hui Yu, Xu Dong, Alejandro Díaz-Holguín, Albert A. Antolin, Huabin Hu

**Affiliations:** † Department of Pharmacy, The Affiliated Hospital of Yangzhou University, Yangzhou University, Yangzhou, 225000, P. R. China; ‡ Information School, University of Sheffield, 211 Portobello, Sheffield, S1 4DP, U.K.; § Science for Life Laboratory, Department of Cell and Molecular Biology, Uppsala University, BMC, Box 596, SE-751 24, Uppsala, Sweden; ∥ Centre for Cancer Drug Discovery, Division of Cancer Therapeutics, The Institute of Cancer Research, London SW7 3RP, U.K.; ⊥ ProCURE, Catalan Institute of Oncology, Oncobell, Bellvitge Institute for Biomedical Research (IDIBELL), L’Hospitalet del Llobregat, Barcelona, Catalonia 08907, Spain

## Abstract

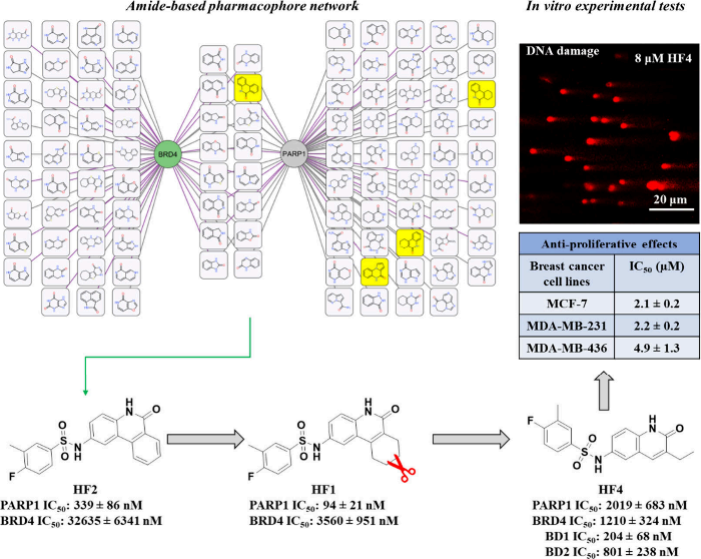

Poly(ADP-ribose) polymerase 1 (PARP1) inhibitors have revolutionized the treatment of many cancers with DNA-repairing deficiencies via synthetic lethality. Advocated by the polypharmacology concept, recent evidence discovered that a significantly synergistic effect in increasing the death of cancer cells was observed by simultaneously perturbating the enzymatic activities of bromodomain-containing protein 4 (BRD4) and PARP1. Here, we developed a novel cheminformatics approach combined with a structure-based method aiming to facilitate the design of dual PARP1-BRD4 inhibitors. Instead of linking pharmacophores, the developed approach first identified merged pharmacophores (a pool of amide-containing ring systems), from which phenanthridin-6(5*H*)-one was further prioritized. Based on this starting point, several small molecules were rationally designed, among which HF4 exhibited low micromolar inhibitory activity against BRD4 and PARP1, particularly exhibiting strong inhibition of BRD4 BD1 with an IC_50_ value of 204 nM. Furthermore, it demonstrated potent antiproliferative effects against breast cancer gene-deficient and proficient breast cancer cell lines by arresting cell cycle progression and impeding DNA damage repair. Collectively, our systematic efforts to design lead-like molecules have the potential to open doors for the exploration of dual PARP1-BRD4 inhibitors as a promising avenue for breast cancer treatment. Furthermore, the developed approach can be extended to systematically design inhibitors targeting PARP1 and other related targets.

## Introduction

Conventional drug discovery mainly focuses on designing chemical entities that are highly selective and potent for their primary target. Such a single-target therapeutic tactic strongly adheres to a direct cause-effect relationship linking the phenotype of disease to the loss of function of a particular protein.^1−3^ However, diseases—especially multifactorial disorders—are frequently perceived as aberrant signaling transductions in a physiological network pathway involving multiple targets.^4^ Accordingly, based on the network pharmacology concept,^5^ multitarget profiles of a compound are gaining momentum in drug discovery pipeline in recent decades and represent an effective strategy for the treatment of complex and multifactorial diseases including oncology and neurodegenerative diseases.^6−9^

**Figure 1:**
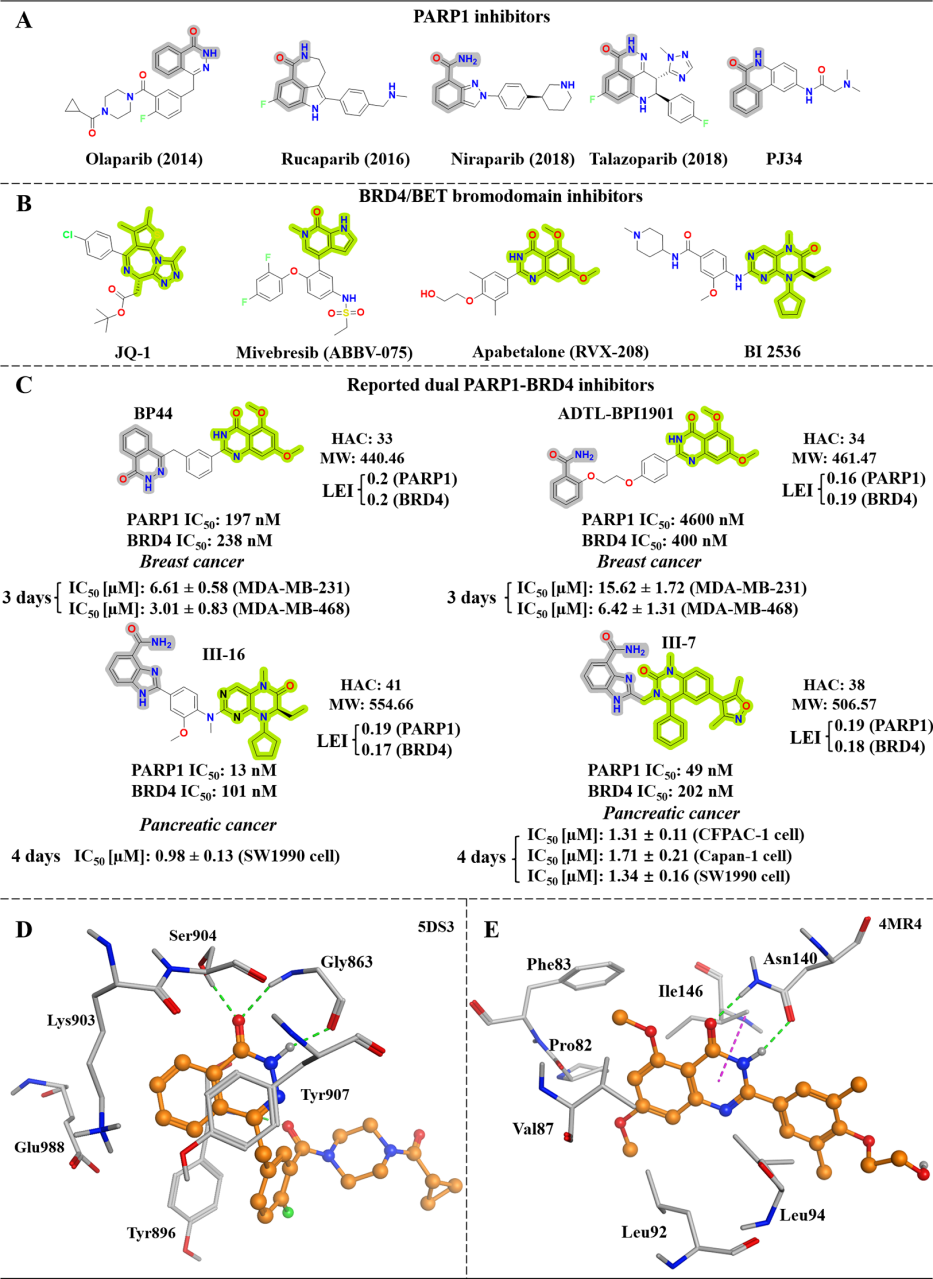
**Exemplary PARP1, BRD4, dual-target inhibitors, and binding mode analysis.** (A) The chemical structures of both FDA-approved and non-FDA-approved PARP1 inhibitors are presented. The essential benzamide pharmacophore responsible for interacting with the PARP1 protein is shaded in gray. The year of their initial FDA approval is denoted in parentheses. (B) The chemical structures of representative inhibitors known to target BRD4 are provided, with the essential pharmacophores highlighted in green. (C) The chemical structures of inhibitors that are known to target both PARP1 and BRD4 are shown. For each inhibitor, the activities (IC_50_ values) for both targets are provided, along with its heavy atom count (HAC), molecular weight (MW), and ligand efficiency index (LEI, calculated by dividing pIC_50_ by HAC).^15−19^ Furthermore, the antiproliferative activities of each dual-target inhibitor are also reported. (D) Analysis of the binding mode of olaparib in complex with PARP1 (PDB code: 5DS3). (E) Binding mode analysis using RVX-208 in complex with BRD4 (PDB code: 4MR4). Only essential residues surrounding the amide-containing scaffold are provided. Protein carbons and ligand carbons are colored gray and orange, respectively. Hydrogen bonds are depicted as green dashed lines, while hydrogen-π interactions are highlighted in magenta.

Poly(ADP-ribose) polymerase 1 (PARP1) has been confirmed as a therapeutical target in oncology whose inhibitors have been successfully approved as a monotherapeutic agent in clinics for the treatment of patients harboring breast cancer gene (BRCA) mutation via the synthetic lethality mechanism.^10,11^ Thus far, the U.S. Food and Drug Administration (FDA) has authorized four PARP1 inhibitors (olaparib, rucaparib, talazoparib, and niraparib) for the treatment of diverse tumor types, such as ovarian cancer and HER2-negative breast cancer.^12^ From a chemical viewpoint, all approved PARP1 inhibitors and PJ34 (a potent and nonselective PARP inhibitor)^13^ have a common feature: the presence of a benzamide group, which can be either a primary or secondary amide, as shown in Figure 1A. This structural element is strategically incorporated into various scaffolds to mimic the nicotinamide moiety of the PARP1 substrate NAD^+^. It possesses the ability to form advantageous hydrogen bonding networks with crucial amino acid residues (Gly863 and Ser904) and participate in π–π stacking interactions with Tyr907, as depicted in Figure 1D. Similar recurrent interactions can be detected in other biologically active PARP1 inhibitors, ensuring their effective inhibition of PARP1 catalytic activity.^14^

Bromodomain-containing protein 4 (BRD4), a member of the bromodomain and extra-terminal domain (BET) family, serves as a pivotal transcriptional and epigenetic regulator.^20^ BRD4 assumes critical functions in the control of gene expression by engaging with acetylated histones, such as histones H3 and H4, through its paired bromodomains (BD1 and BD2). In this manner, it orchestrates the recruitment of enzymes responsible for chromatin regulation.^21^ BRD4 has emerged as a promising therapeutic target in several malignancies, including the clinically aggressive triple-negative breast cancer.^22−24^ Multiple BRD4 inhibitors have been revealed, such as JQ-1^25^ and mivebresib^26^ (Figure 1B). Given the high sequence identity of bromodomains, many of the reported inhibitors lack high selectivity when it comes to targeting BET bromodomains, often affecting multiple members of the BET bromodomains (pan-BET inhibitors), hence in certain degree compromising the therapeutic efficacy of BRD inhibitors in clinic development.^27^ Alternatively, certain BET inhibitors that show specificity for either BD1 or BD2 such as apabetalone, also known as RVX-208, which specifically inhibits BET-BD2 (Figure 1B),^28^ were identified and exhibited some advantages over pan-BET inhibitors. Furthermore, there is another distinct class of BRD4 inhibitors represented by a potent polo-like kinase 1 inhibitor known as BI 2536 (Figure 1B). BI 2536 has shown strong inhibitory activity against BRD4 at the nanomolar level.^29,30^ These inhibitors are characterized by the presence of acetylated lysine mimetics with the incorporation of an amide or amide-like functional group, which frequently interacts with a conserved asparagine residue, such as Asn140, within the binding pocket.^27^ Illustrative examples of such inhibitors include RVX-208 (Figure 1E) and BI 2536.

Recent advancements have highlighted the substantial role played by BRD4 in the repair and propagation of DNA damage via regulating the activities of other repair machineries, such as TOPBP1, BRCA1, and Rad51.^18,31^ Hence, inhibiting BRD4 activity increases the sensitivity of tumors with DNA repair deficiencies to PARP1 inhibitors, a phenomenon demonstrated in breast cancer cells both *in vitro* and in xenograft tumor models.^18^ This synergistic antitumor effect has also been confirmed in other cancer types, including pancreatic cancer, cholangiocarcinoma, and BRCA-proficient ovarian cancers.^15,31,32^ Consequently, the simultaneous modulation of both targets by using a single ligand represents a promising strategy for combating cancer.

Systematic development of compounds with the ability to manipulate multiple targets of interest is still far from being a standard and widely adopted approach in the design process. Typically, the strategy for designing dual-target compounds involves molecular hybridization, a technique that combines, merges, or links essential pharmacophores from different targets of interest.^1,33^ Several research investigations have been reported to develop dual PARP1-BRD4 inhibitors for addressing pancreatic cancer^15^ and triple-negative breast cancer.^18^Figure 1C provides an overview of the chemical structures of these dual PARP1-BRD4 inhibitors.^15−18^ These inhibitors have displayed remarkable antitumor effectiveness *in vitro* and *in vivo*, highlighting their potential therapeutic significance. An in-depth examination of these compounds reveals a common design approach, namely, the integration of two pharmacophores associated with PARP1 and BRD4. This strategy frequently led to the rapid attainment of dual-target profiles. However, if the pharmacophores are not carefully selected, their combination can lead to the development of bulky molecules with poor drug-like properties, such as a large molecular weight (≥500, as seen with III-16 and III-7 in Figure 1C) and increased hydrophobicity, indicated by a cLogP value greater than 5.0 (III-7, Figure 1C). Additionally, all known PARP1-BRD4 inhibitors (Figure 1C) showed lower ligand efficiency index (LEI ≤ 0.2),^19^ along with imbalanced dual-target activity profiles, such as those observed with ADTL-BPI1901. Therefore, new cheminformatics approaches to facilitate the rational design of dual inhibitors leveraging available Big Data are thus needed.

In this study, we were inspired by the chemical patterns evident in the inhibitors of PARP1 and BRD4 (as illustrated in Figure 1A,B), as well as the consistent hydrogen bond interactions with Gly863 (as shown in the olaparib-PARP1 complex, Figure 1D) and Asn140 (as evidenced in the complex formed by RVX-208 and BRD4, Figure 1E). This prompted us to contemplate that, rather than linking two pharmacophores, it might be more advantageous to identify a common key structural element as a starting point for the design of more drug-like molecules. Consequently, we developed a new cheminformatics pipeline to facilitate the rational design of inhibitors active against both PARP1 and BRD4. Initially, the approach involved the identification of all potential informative amide-containing scaffolds by fragmenting publicly available known PARP1 and BRD4 inhibitors. Subsequently, these scaffolds were systematically arranged into a network, facilitating the discovery of the shared pharmacophore. Using this scaffold as a foundation, several compounds were prioritized by using molecular docking and analyzing structure–activity relationship (SAR) of known bioactive compounds and then experimentally validated through an iterative design-make-test-analyze cycle. Among them, HF4 emerged as a promising candidate, displaying single-digit inhibitory activity against both targets, further validated by its strong antiproliferative effect against three breast cancer cells. Due to the significant impact of PARP1 in oncology, the newly developed cheminformatics approach presented in this study can be similarly employed for exploring inhibitors targeting PARP1 and other associated targets, including PI3Kα and EZH2.^34^ Comprehensive details of these results are presented herein.

## Results and Discussion

### Occurrence of Frequency of Amide-Based Scaffolds in PARP1 and BRD4

**Figure 2:**
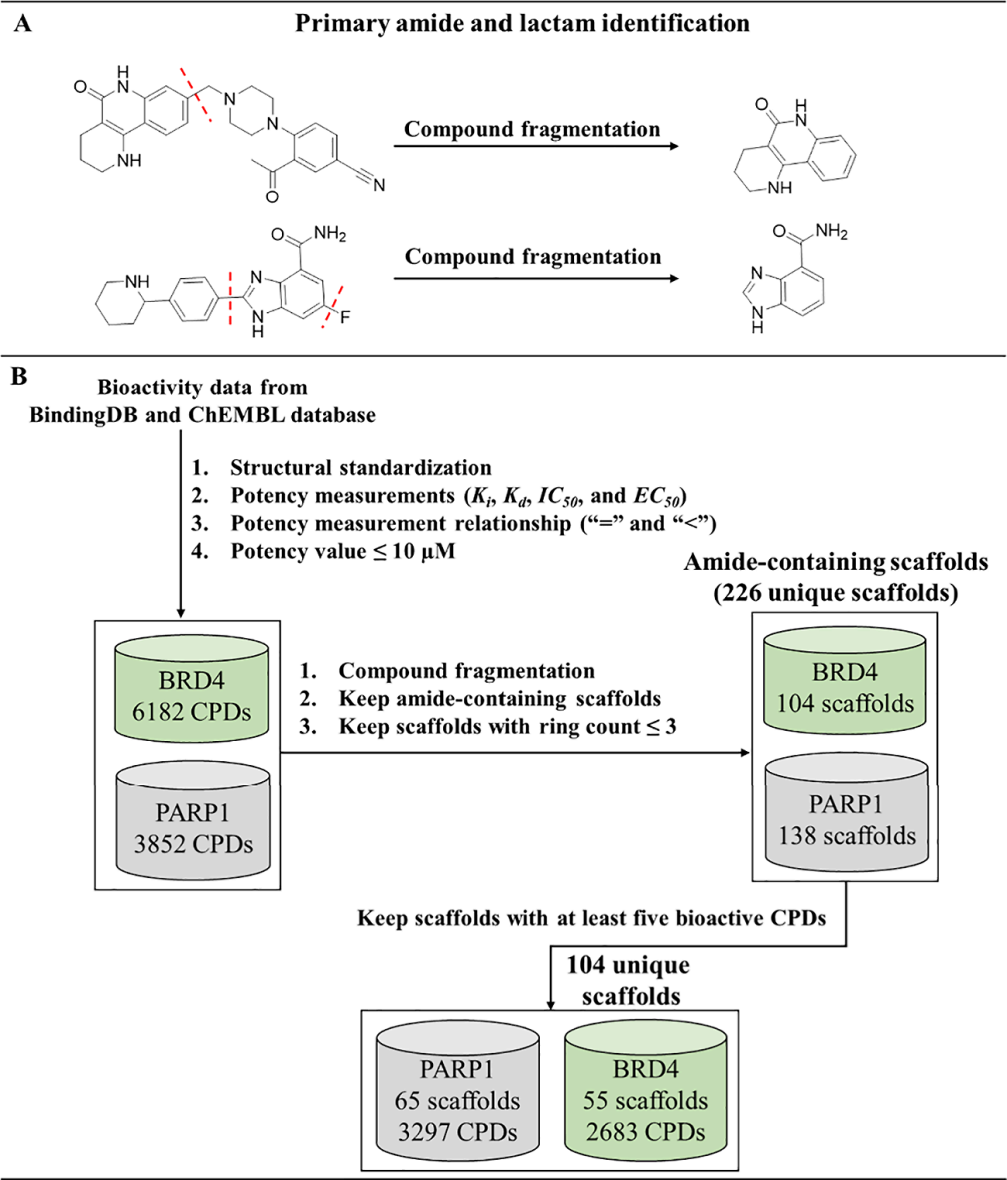
**Amide-containing pharmacophore identification.** (A) Two different amide pharmacophores were individually recognized through the cleavage of bonds (red dashed line) in exemplary compounds. (B) The flowchart depicts the sequential filtrations aimed at obtaining amide scaffolds featuring bicyclic or tricyclic ring systems. CPD: compound.

The rich reservoir of bioactivity data stored in publicly accessible databases, such as ChEMBL^35^ and BindingDB,^36^ serves as a valuable resource for uncovering significant insights into SAR information. In this research, we have gathered all documented bioactive compounds associated with PARP1 or BRD4 activities from these two databases. Following the data preprocessing protocol (see Materials and Methods for details), a total of 3852 and 6182 compounds were obtained for PARP1 and BRD4, respectively. This collection of bioactive compounds from ChEMBL and Binding DB is intended to facilitate the identification of shared pharmacophore elements between both targets. To achieve this, the curated bioactive compounds undergo a decomposition process, involving the cleavage of all acyclic single bonds except for the bond connecting the primary amide to the ring system (see Materials and Methods for details and Figure 2A). To obtain informative amide-containing scaffolds, the following filtering criteria were applied: (1) from the scaffold pool, only bicyclic and tricyclic ring systems with an amide functional group (primary amide or lactam), which are key features in PARP1 inhibitors (see Figure 1A), were selected; (2) only the amide scaffolds present in a minimum of five bioactive compounds were taken into consideration (Figure 2B). The second criterion was applied to ensure that the selected scaffolds have reliable known SAR information, which is valuable for guiding rapid optimization. Although this criterion reduced the number of unique amide-based ring systems from 226 to 104 (Figure 2B), none of the excluded scaffolds are shared between PARP1 and BRD4. Additionally, 84% of these excluded scaffolds are associated with two or fewer compounds with single-target activity. To facilitate structure-based compound design, we retrieved confirmed binding modes for ligands associated with both targets—69 for PARP1 and 449 for BRD4 (details are provided in the Materials and Methods section)—from the PDB database.^37^ These data were processed by using the same protocol as the bioactive compounds from the ChEMBL and BindingDB databases.

**Figure 3:**
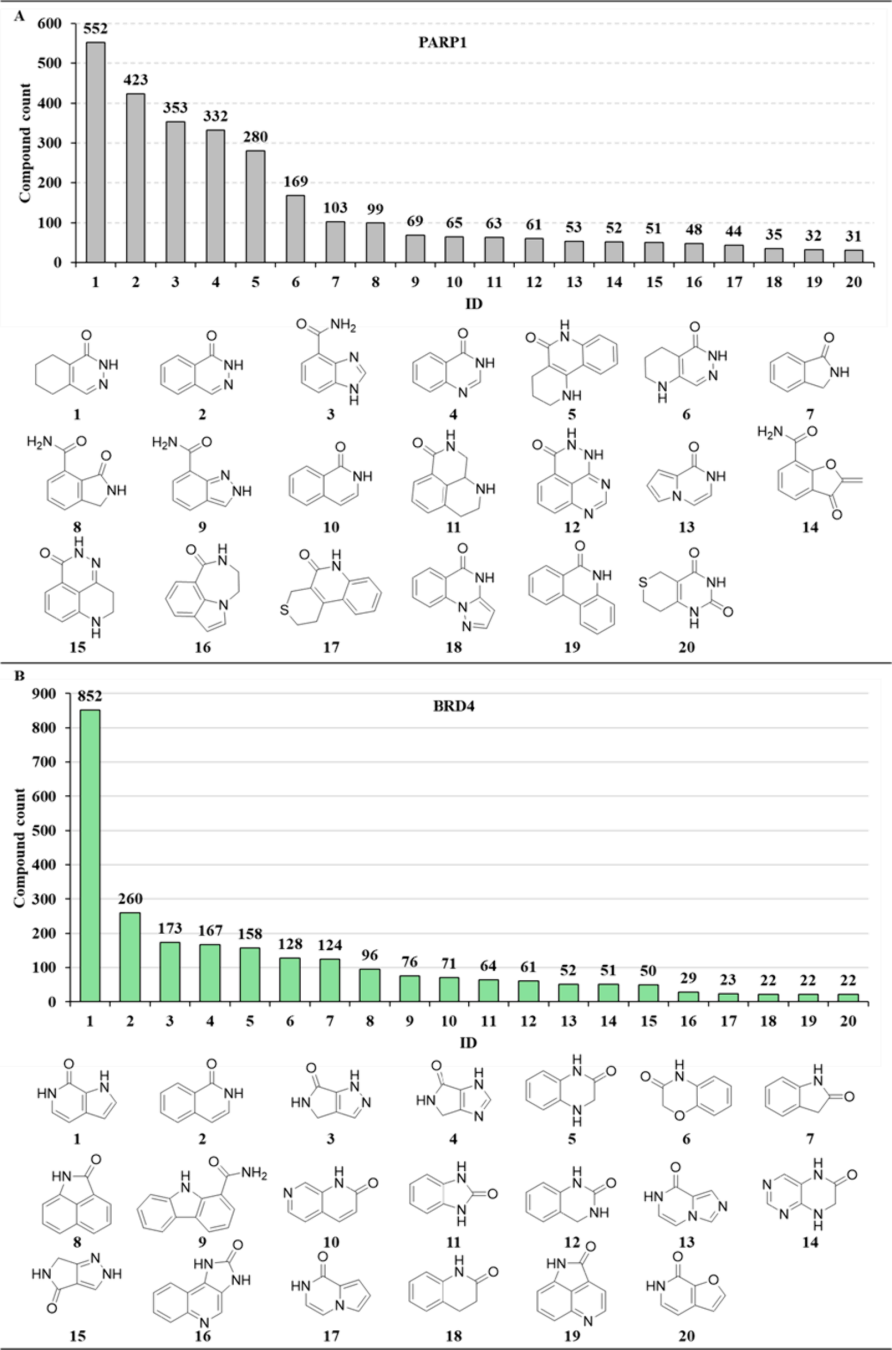
**Most favored amide-containing scaffolds in PARP1 and BRD4.** The top 20 amide-based scaffolds, as favored in PARP1 (A) and BRD4 (B), have been listed. The bar chart provides the occurrence of frequency of these scaffolds in reported bioactive inhibitors, with the number at the top of each bar indicating the quantity of inhibitors featuring the specific scaffold.

Concerning PARP1, a selection of 65 bicyclic and tricyclic pharmacophores that contain amides was obtained, which were derived from 3297 PARP1 inhibitors (Figure 2B), resulting in an average compound-to-core ratio (calculated by dividing the number of amide-containing compounds by the total number of amide-containing scaffolds) of approximately 51:1. These amide-containing pharmacophores constitute 85.6% of known PARP1 compounds, highlighting their extensive utilization in the development of PARP1 compounds within medicinal chemistry endeavors. In Figure 3A, the top 20 preferred scaffold structures are presented, comprising roughly 72.9% of the reported inhibitors, along with their corresponding chemical structures displayed at the bottom. The most commonly employed structural framework is 5,6,7,8-tetrahydrophthalazin-1(2*H*)-one (scaffold 1), which appears in 552 bioactive compounds. Following closely is phthalazin-1(2*H*)-one (scaffold 2) with 423 inhibitors for PARP1, and 1*H*-benzo[d]imidazole-4-carboxamide (scaffold 3) with 353 inhibitors. Out of these preferred structural patterns, 12 are characterized by bicyclic ring structures and eight exhibit tricyclic frameworks. Notably, specific favored frameworks incorporate primary amides, as exemplified by scaffolds 3, 8, 9, and 14, while others incorporate lactams. Furthermore, some of these favored scaffolds can be identified in FDA-approved PARP1 inhibitors such as scaffold 2 in olaparib, scaffold 9 in niraparib, and scaffold 15 in talazoparib.

In terms of BRD4, 55 amide-containing core structures have been identified within a collection of 2683 BRD4 inhibitors, comprising 43.4% of all BRD4 inhibitors (Figure 2B). We noticed that around 56% of BRD4 compounds were excluded from the analysis due to the lack of an amide-based ring system (key features for PARP1 inhibitors interacting with critical residues Gly863 and Ser904 at the active site; Figures 1A,D). Upon further examination, Figure S1 presents the top 10 non-amide-based scaffolds found in BRD4 inhibitors. As expected, these structural features make them unsuitable for designing PARP1 inhibitors, unless the scaffolds undergo further fine-tuning (e.g., embedding primary amide or lactam features). Figure 3B illustrates the top 20 most prevalent core structures. The most frequently occurring amide core structure is 1,6-dihydro-7*H*-pyrrolo[2,3-*c*]pyridin-7-one (scaffold 1), which is present in 852 BRD4 inhibitors, such as mivebresib. Following closely is isoquinolin-1(2*H*)-one (scaffold 2) featured in 260 bioactive compounds.

In contrast to PARP1, where the primary or secondary amide is frequently observed, it is important to highlight that within BRD4, the amide functional group can also manifest as a tertiary amide, exemplified by compounds such as mivebresib and BI 2536 (Figure 1B). Nonetheless, upon comparing the top 20 amide core structures in both PARP1 and BRD4, common chemical features are revealed that may serve as compelling starting points for the development of dual-target compounds.

### Scaffold Prioritization for Dual-Target Compound Design

**Figure 4:**
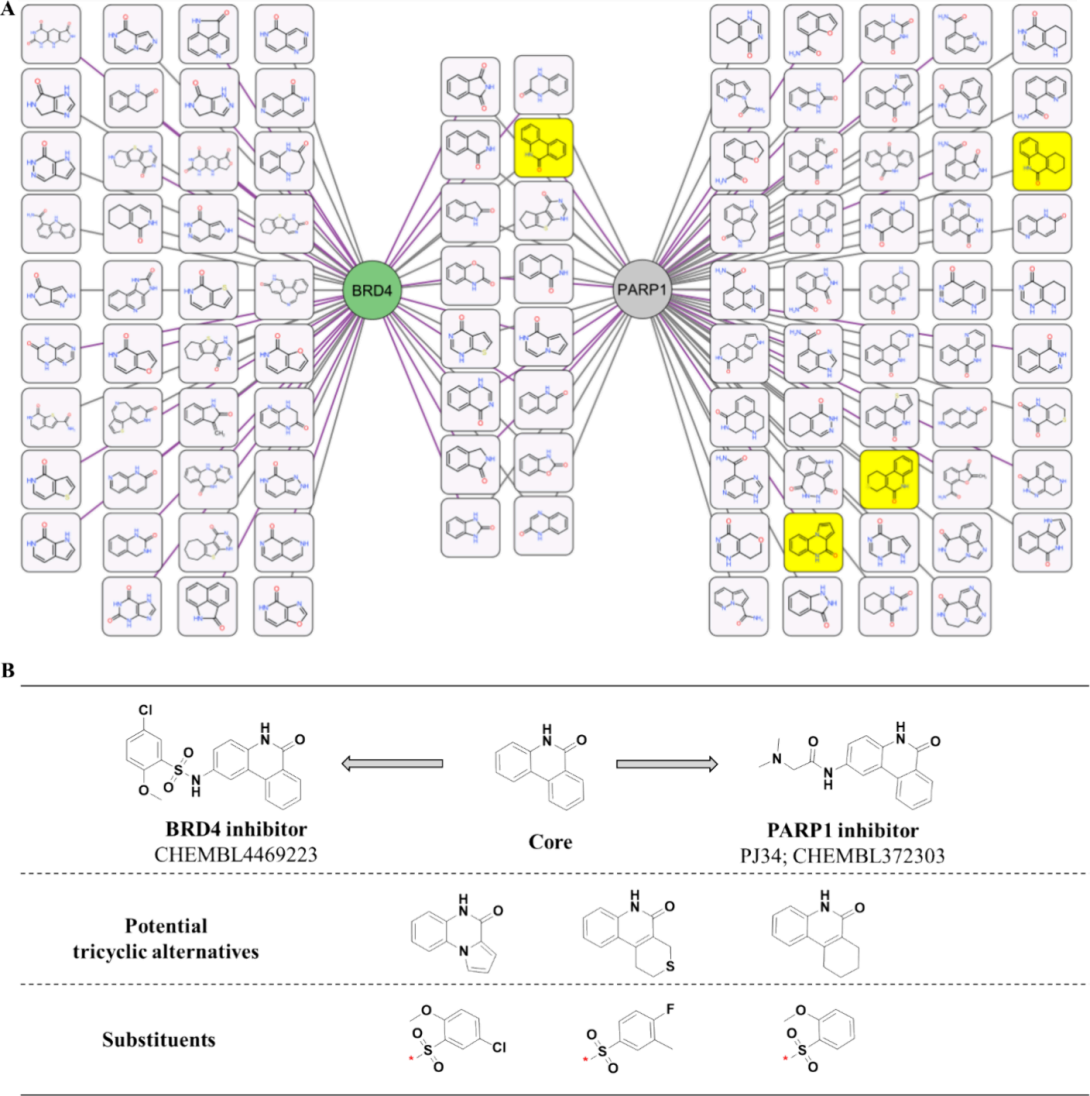
**Shared pharmacophore identification and prioritization.** (A) Amide-functionalized scaffolds were generated by fragmenting compounds sourced from PARP1 and BRD4. Only those scaffolds featuring amide functional groups were retained, and they were visually represented in a network format. In this network, rectangular shapes represent scaffolds, while circular nodes depict targets. A scaffold is connected to a target if it originates from a specific target. Additionally, edges are colored purple if at least one scaffold’s parent compounds were cocrystallized with the corresponding target protein; otherwise, they are colored gray. The examination and prioritization of shared scaffolds in the middle of the network are based on factors, such as the substitution site and crystallographic information. Visual inspection of the shared scaffolds led to the identification of a tricyclic ring system, i.e., phenanthridin-6(5*H*)-one highlighted in yellow. (B) The original compounds linked to this identified scaffold are provided along with their ChEMBL IDs. As alternatives to the identified scaffold, three other tricyclic amide-containing scaffolds from PARP1 (colored yellow in Figure 4A) are also considered as potential candidates for scaffold hopping. Additionally, two other substituents, similar to CHEMBL4469223 in BRD4, were also taken into consideration for the compound design and SAR analysis.

To facilitate the identification of optimal chemical scaffolds, all identified amide-containing scaffolds were organized globally in a network manner (Figure 4A). Within the network, nodes symbolized the names of target genes and amide scaffolds. A scaffold was linked to a target gene if the parent compound of the scaffold exhibited activity against that specific target. The network analysis resulted in the discovery of 104 unique amide scaffolds. Among them, 39 belonged exclusively to BRD4, while 49 were exclusive to PARP1 (Figure 4A). Sixteen scaffolds were identified as shared between both targets, prompting a detailed examination. First, 10 out of 16 scaffolds, whose parent compounds are associated with at least one PDB structural complex (Figure 4A, purple edge), allow for structure-based compound design. Upon examination of these structural complexes and ligands that share the scaffolds, we found that most are less prioritized due to differences in the vectors of substitution sites, such as in the first pair (Figure S2). However, we identified a pair that shares the same growth vector on the scaffold (Figure S2, bottom). Despite this, the BRD4 inhibitor RVX-208 showed no activity in our test against PARP1 at 10 μM. It is worth noting that our analysis does not suggest that using these scaffolds to design dual PARP1-BRD4 inhibitors is impossible but rather that it may require additional medicinal chemistry efforts and more detailed SAR analysis to successfully identify dual-target inhibitors (such as the second pair in Figure S2). Following this comprehensive analysis and as a proof of concept, our focus was directed toward a tricyclic amide scaffold, specifically phenanthridin-6(5*H*)-one (highlighted in yellow at the center of Figure 4A), as the starting point for the development of dual-target inhibitors. This decision stemmed from the observation that compounds with this shared pharmacophore, such as CHEMBL4469223 for BRD4 and CHEMBL372303 for PARP1, have a common substitution site (growth vector; Figure 4B). Moreover, there is an available crystal structure of the parent compound complexed with PARP1 (CHEMBL372303, PDB code: 4UXB), enabling compound design through a structure-based approach. In addition to this core framework, we also investigated three additional alternative tricyclic scaffolds derived from PARP1 (as shown in Figure 4B), highlighted in yellow on the right side of Figure 4A. These were evaluated as potential choices for scaffold hopping within the context of phenanthridin-6(5*H*)-one. Additionally, by incorporating the substituent originating from the BRD4 inhibitor CHEMBL4469223, our investigation also encompassed an examination of two analogous substituents, as visually presented in Figure 4B.

### Compound Design with the Aid of Molecular Docking

**Figure 5:**
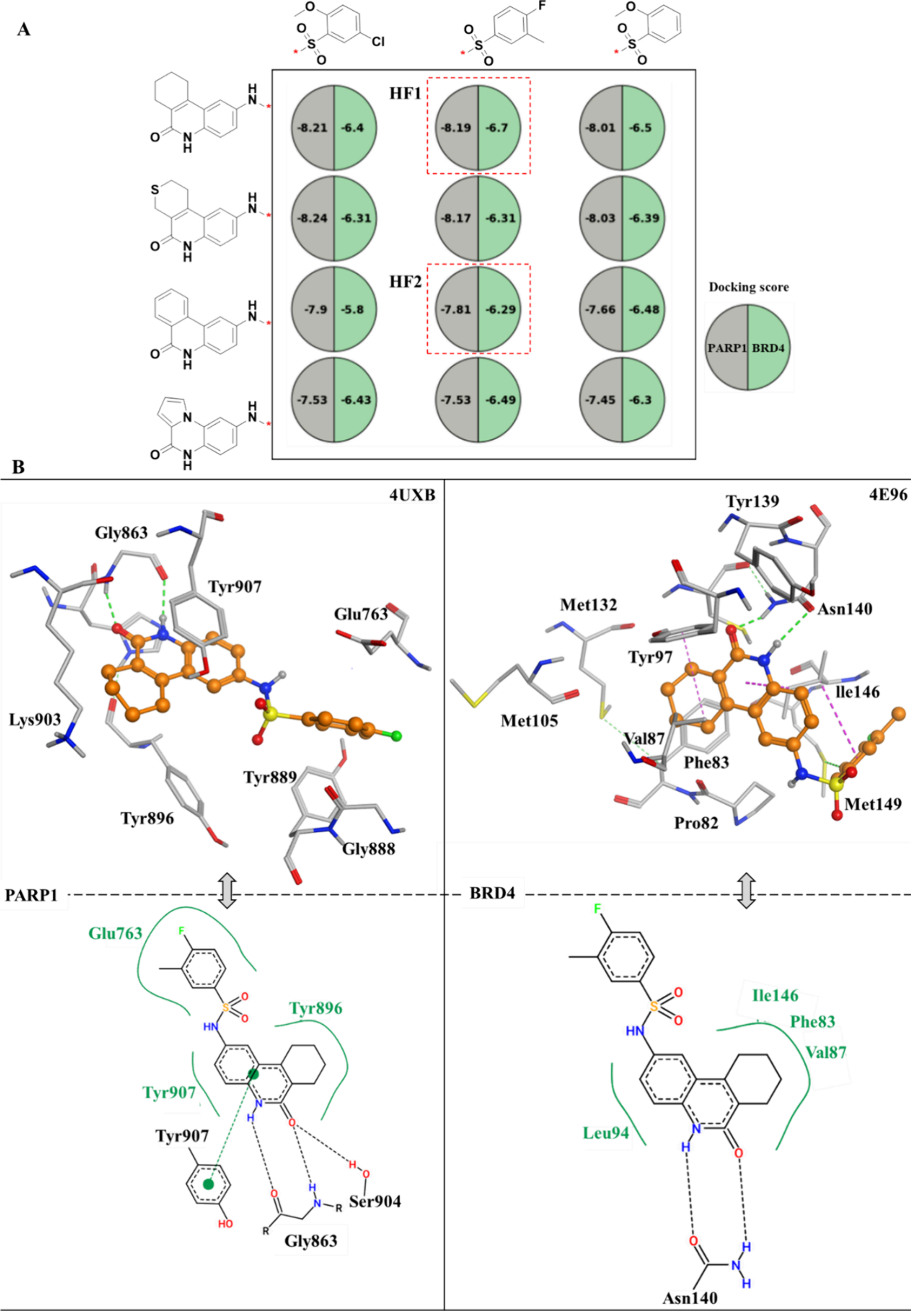
**Virtual analogues exploration and prioritization via molecular docking.** In (A), a data set of 12 analogues was created by pairing three substituents with four distinct scaffolds. This data set was structured similarly to a SAR matrix, with the substituents corresponding to columns and the scaffolds to rows. Within each cell of this matrix, a pie chart was employed. Each pie chart was divided into two sections, each containing numerical values representing the docking scores for the respective target. Based on these docking scores and an analysis of the putative binding modes, compound HF1 was selected for synthesis alongside HF2, which shared the same scaffold as phenanthridin-6(5*H*)-one and served as a control. In (B), the proposed binding modes of HF1 in two protein targets, PARP1 (PDB code: 4UXB) and BRD4 (PDB code: 4E96), were presented. The 3D representation of the binding site showed the protein carbons in gray and the ligand carbons in orange. Hydrogen bonds were illustrated as green dashed lines, while hydrogen-π interactions were highlighted in magenta. Additionally, a 2D interaction diagram for each complex was provided, generated using PoseView (https://proteins.plus/). In these 2D diagrams, hydrophobic contacts between the ligand and protein were depicted as spline segments, and hydrogen bond interactions were indicated by dashed black lines.

By considering four primary scaffolds and three different substituents (Figure 4B), 12 analogues through the fusion of these four scaffolds and three substituents were obtained. These analogues were then organized in a manner reminiscent of a SAR matrix data structure (as shown in Figure 5A).^38^ Designed analogues underwent further selection through molecular docking analysis, ultimately leading to the decision to synthesize compound HF1 owing to its high docking score against both targets and reasonable putative binding modes. The proposed binding modes of HF1 with PARP1 and BRD4 are illustrated in Figure 5B. As anticipated, the tricyclic amide scaffold establishes chelated hydrogen bonds with backbone of Gly863 and engages in π–π stacking interactions with Tyr907, which are conserved interactions commonly observed among PARP1 inhibitors serving to stabilize the conformation of the binding pose.^14^ In the case of the proposed binding pose within the BRD4 protein, the scaffold similarly forms a chelated hydrogen bond with the side chain of residue Asn140. Additionally, there is evidence of an enhanced hydrophobic effect due to the presence of several nonpolar residues in the vicinity of the binding pockets, including Val87, Phe83, and Ile146. Furthermore, the comparatively less rigid, nonplanar tricyclic scaffold emerges as a more advantageous selection for both the binding pockets of PARP1 and BRD4, owing to its inherent flexibility in accommodating the hydrophobic environments. Taken together, these potential favorable interactions strongly suggest that the selected virtual analogue (HF1) has the potential to exhibit dual-target profile. Consequently, we made the decision to proceed with the synthesis of HF1 for experimental validation. Furthermore, HF2, featuring a shared scaffold initially identified in both targets (phenanthridin-6(5*H*)-one, as shown in Figure 4B), was also synthesized.

### *In Vitro* Inhibitory Activities of Designed Compounds against PARP1 and BRD4

**Figure 6:**
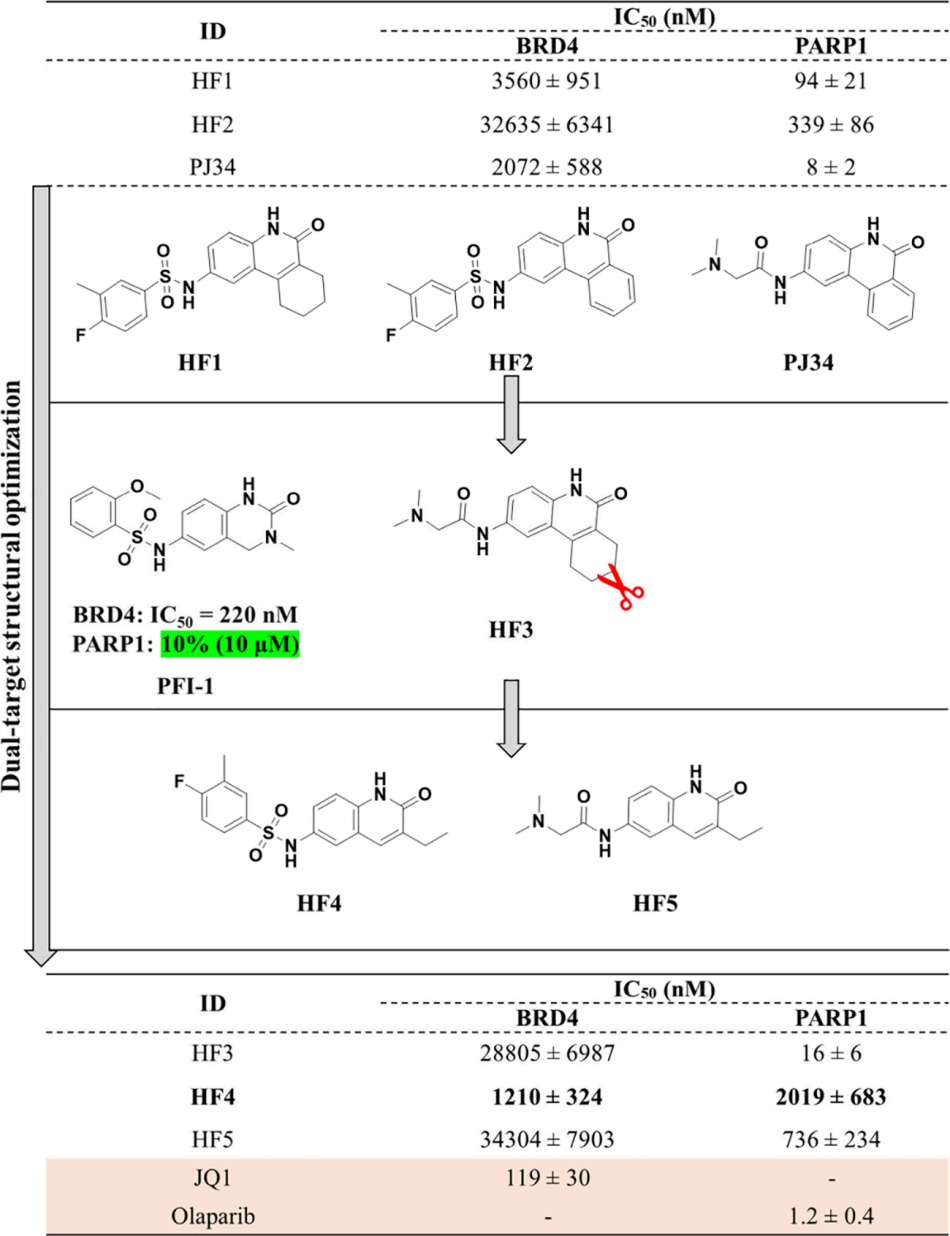
**Inhibitory activities of designed compounds and dual-target profile optimization.** Flowchart of rationally designing dual-target compounds (HF1-HF5) were shown. For designed compounds, IC_50_ values were provided.

The inhibitory effects of both enzymes were measured for HF1 and HF2. As illustrated in Figure 6, both HF1 and HF2 displayed strong inhibition of PARP1, with HF1 demonstrating superior potency with an IC_50_ value of 94 nM. When evaluating their activities against BRD4, they exhibited significantly lower potency compared with their impact on PARP1. However, HF1, featuring a nonplanar scaffold, exhibited strong affinity with an IC_50_ value of 3.5 μM against BRD4, making it 10 times more potent than HF2 with a planar aromatic ring system. The large discrepancy in BRD4 activities could be attributed to the nonplanar scaffold of HF1, which appears to fit more effectively into the hydrophobic environment formed by Val87, Phe83, and Ile146 (Figure 5B, right). Moreover, HF1 demonstrated higher solubility during activity testing, with a predicted cLogP value of 3.66 (calculated using the RDKit package^39^), which is lower than HF2’s value of 3.93 (Figure S3), potentially contributing to its performance. The dual-target profile of HF1 demonstrated a promising start of our approach. Before proceeding further, we made the decision to examine PJ34, a potent and nonselective PARP1 inhibitor,^13^ to assess its impact on BRD4 inhibition. As depicted in Figure 6 (upper panel), PJ34, featuring a planar pharmacophore (as HF2), displayed comparable BRD4 activity to HF1, albeit exhibiting roughly 10-fold greater activity against PARP1. Given the strong PARP1 activity and relatively low micromolar BRD4 activity of PJ34 relative to HF1 and HF2, we contemplated whether substituting PJ34’s scaffold with that of HF1 could synergistically enhance BRD4 affinity while maintaining strong PARP1 affinity.

### Dual-Target Activity Profile Optimization

As a consequence, compound HF3 (depicted in Figure 6) was synthesized. Unfortunately, the evaluation of HF3’s affinity indicated that fusing HF1’s scaffold with PJ34’s substituent did not synergistically enhance BRD4 inhibitory activity, as evident from the IC_50_ value of approximately 28 μM. Nevertheless, HF3 maintained its potent PARP1 inhibition, with an IC_50_ value of 16 nM. Inspired by the structure of PFI-1, a high-quality chemical probe of BET proteins,^40,41^ this compound featured a bicyclic ring structure that included an amide group and exhibited an IC_50_ value of 220 nM against BRD4.^40^ In our initial analysis, we concentrated on assessing its influence on PARP1 activity at a 10 μM concentration. However, it showed only a minor capacity to impact PARP1, resulting in an inhibition rate of a mere 10% at 10 μM concentration (Figure 6, middle).

Taking advantage of the superior BRD4 activity exhibited by PFI-1 and the enhanced PARP1 inhibition seen with HF3, we set out to optimize the tricyclic scaffold through scaffold truncation, resulting in the design of compounds HF4 and HF5. The inhibitory activities of these two compounds are depicted in Figure 6 (bottom). Notably, HF4, characterized by the key scaffold aromatization of PFI-1, observed a significant increase in its ability to inhibit PARP1 activity, as evidenced by the activity rising from 10% inhibition (tested at 10 μM concentration) to approximately 2.0 μM (IC_50_ value). However, this enhancement came at the cost of around 5-fold reduction in its capacity to inhibit BRD4. Conversely, the transition from a tricyclic scaffold to a bicyclic structure led to an approximately 20-fold decrease in effectiveness against PARP1, as indicated by the IC_50_ values of 94 nM for HF1 and ∼2 μM for HF4. With respect to compound HF5, it managed to attain a sub-micromolar IC_50_ value of 736 nM for PARP1, but its performance against BRD4 was less impressive, as it yielded an IC_50_ value of approximately 34.3 μM. A brief summary of these compounds, including their cLogP properties and activities, is presented in Figure S3. Although compounds HF3-HF5 were designed using a ligand-based method (integrating iterative analysis of activity results and known inhibitors), the docking scores for these compounds generally support their corresponding bioactivities (Figure S3). As illustrated in the predicted binding modes of HF4 (Figure S4), a conserved hydrogen bonding network is observed with the key residues Gly863 in PARP1 and Asn140 in BRD4 (Figure S4).

Moreover, we assessed the inhibitory effects of HF4 on three BET isoforms and one non-BET BRD (BRD1) to investigate its selectivity for BRD isoforms. As depicted in Table 1, HF4 exhibited potent inhibition of BRD4 in comparison to other BRDs. It is noteworthy that HF4 demonstrates some selectivity in inhibiting BRD4 BD1 and BD2, with BRD4 BD1 exhibiting a more potent inhibition, with an IC_50_ value of 204 nM (four times more potent than that of BRD4 BD2).

**Table 1:** Profiling the isoforms of BRD in compound HF4^a^

	IC_50_ (nM)	
Isoform	HF4	JQ-1
BRD1	55618 ± 13928	36427 ± 7505
BRD2	6831 ± 2198	673 ± 229
BRD3	4792 ± 1365	303 ± 98
BRD4	1210 ± 324	119 ± 30
BRD4 BD1	204 ± 68	32 ± 10
BRD4 BD2	801 ± 238	89 ± 34

a The reported IC_50_ values (nM) represent the averages derived from at least two separate experiments accompanied by standard deviation.

In summary, among the several compounds we designed, HF4 emerged as a promising candidate, exhibiting the strongest BRD4 activity at 1.2 μM (IC_50_). The selection of HF4 is based on the observation that the previously reported dual PARP1-BRD4 inhibitor ADTL-BPI1901, despite showing a less balanced activity profile by inhibiting PARP1 less effectively (IC_50_ = 4600 nM), remains potent against BRD4 (IC_50_ = 400 nM) and retains acceptable cellular activity (Figure 1C). Moreover, it features an unbulky chemical structure and exhibits well-balanced, single-digit inhibitory effects against both PARP1 and BRD4, representing good starting points for further optimization. Consequently, we decided to explore its antiproliferative activity on breast cancer cells (MCF-7, MDA-MB-231, and MDA-MB-436).

### HF4 Demonstrated Potent Antiproliferative Effects on Various Breast Cancer Cell Lines

**Figure 7:**
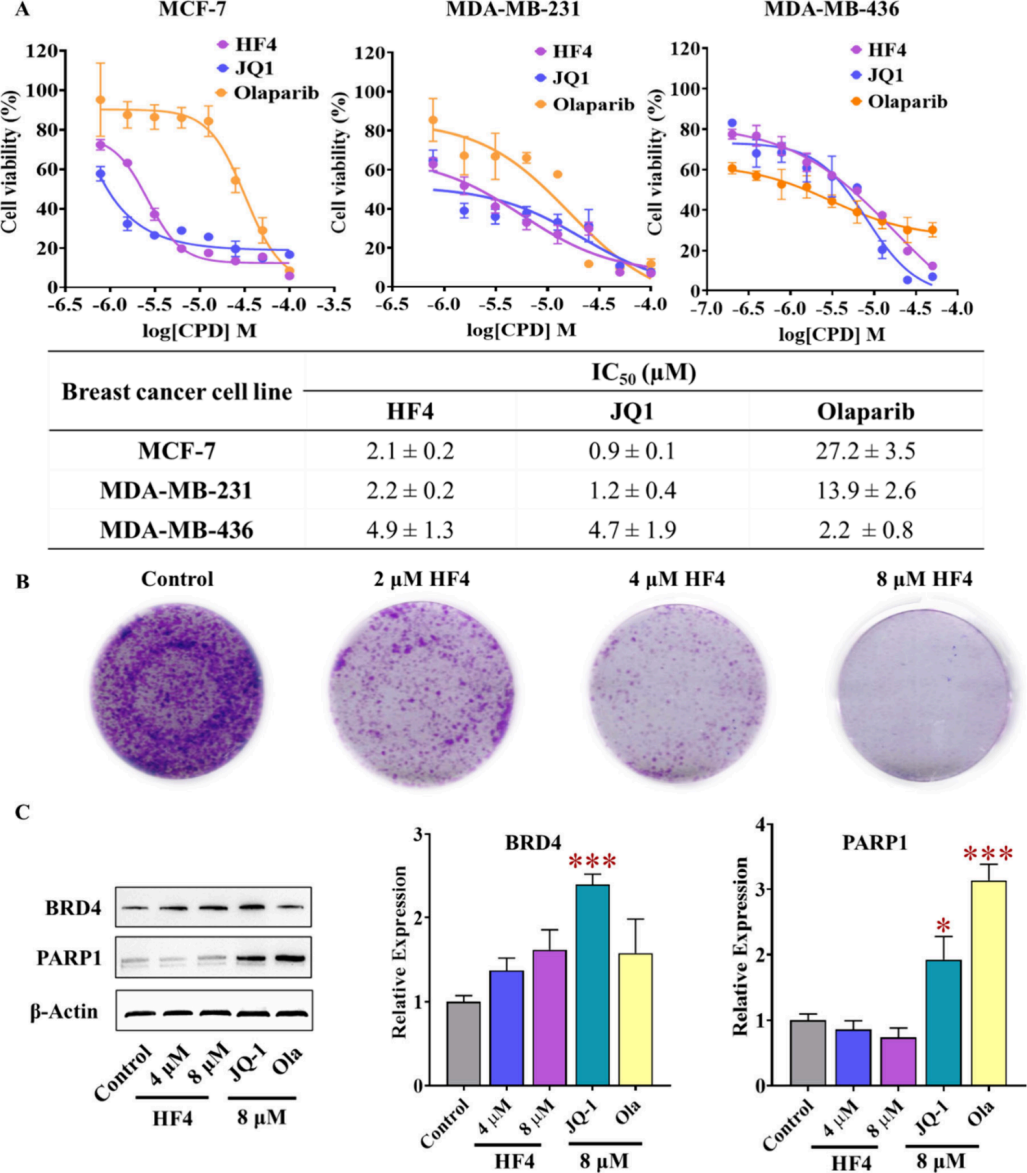
**The antitumor effect of HF4*****in vitro*****.** (A) HF4’s antitumor efficacy was assessed in MCF-7, MDA-MB-231 (wild-type BRCA breast cancer cells), and MDA-MB-436 (BRCA mutant breast cancer cells) over a 4-day treatment period. (B) HF4’s impact on the formation of MCF-7 cell colonies was evaluated. (C) Western blot analysis was performed to investigate the effects of HF4 on the expression levels of BRD4 and PARP1 in MCF-7 cells. Error bars represent the standard deviation; *p < 0.05, **p < 0.01, ***p < 0.001, compared to the control groups.

To assess the combined efficacy of inhibiting BRD4 and PARP1 in cellular settings, we investigated the antiproliferative activities of HF4 in both BRCA mutant and wild-type BRCA breast cancer cells. As illustrated in Figure 7A, HF4 exhibited a significant inhibition of proliferation in BRCA-proficient breast cancer cell lines, specifically MCF-7 and MDA-MB-231, with IC_50_ values of 2.1 and 2.2 μM, respectively. The impact of HF4 on MDA-MB-231 and MCF-7 cancer cells was comparable to that of JQ1 and notably superior to olaparib. In line with expectations, olaparib demonstrated enhanced efficacy in the BRCA-deficient cell line (MDA-MB-436) due to synthetic lethality, achieving an IC_50_ value of 2.2 μM. Our designed compound, HF4, also exhibited potent antiproliferative effects on MDA-MB-436 cells, with an IC_50_ value of 4.9 μM. Simultaneously, as HF1 displayed potent inhibition of PARP1 and exhibited low micromolar inhibition against BRD4 (Figure 6), we evaluated its antiproliferative effects in MCF-7 and MDA-MB-231 cell lines. When compared to HF4, it was found that HF1 displayed limited inhibition in both cell lines (see Figure S5), with IC_50_ values of 11.4 and 31.9 μM, respectively. Therefore, HF4 characterized by an unbulky chemical structure (HAC = 25 and MW = 360.4 Da) distinguishes itself with enhanced ligand efficiency (with a ligand efficiency index^19^ of 0.24 against BRD4 and 0.23 against PARP1), surpassing other dual PARP1-BRD4 inhibitors reported previously (ligand efficiency index ≤ 0.2 for both PARP1 and BRD4, as shown in Figure 1C). Additionally, HF4 demonstrated notable efficacy across various breast cancer cell lines, surpassing or matching the antiproliferative effects observed with previously reported dual-target inhibitors (Figure 1C).

Moreover, the colony formation assay was carried out to evaluate HF4’s enduring antiproliferative effect on representative breast cancer cell. As depicted in Figure 7B, the findings elucidate a marked decline in colony formation within MCF-7 cells after a seven-day exposure to 2 μM of HF4. Significantly, this reduction exhibits a clear dose-dependent trend, with nearly no visible colonies when the HF4 concentration reaches 8 μM. These results collectively emphasize the potential of HF4 as a compelling candidate warranting further exploration.

### HF4 Demonstrates a Limited Impact on the Expression of BRD4 and PARP1 on MCF-7 Cell Line

Apart from its strong antiproliferative effects in tested breast cancer cell lines, it was necessary to consider the likelihood of HF4 inducing drug resistance. In cells that had developed resistance to BRD4 or PARP1, an increase in the expression levels of BRD4 and PARP1 was consistently observed.^42,43^ This prompted an investigation into the impact of HF4 on the expression of these two proteins in MCF-7 cells. As illustrated in Figure 7C, when examining BRD4 expression, it is evident that JQ1 significantly enhances its levels, whereas HF4 also results in an increase but to a much lesser extent than JQ1. Concerning the impact on PARP1 expression, both JQ1 and olaparib treatment led to a significant increase. In contrast, HF4 has a limited effect on PARP1 expression and demonstrates a slight dose-dependent reduction in its levels.

Taken together, the upregulation of both BRD4 and PARP1 following JQ1 and olaparib treatment suggests the potential for the development of adaptive resistance. In contrast, the designed compound HF4 appears to have a lower likelihood of inducing adaptive resistance compared with JQ1 or olaparib.

### HF4 Enhances Apoptosis in Breast Cancer Cells by Modulating Apoptosis-Related Proteins

**Figure 8:**
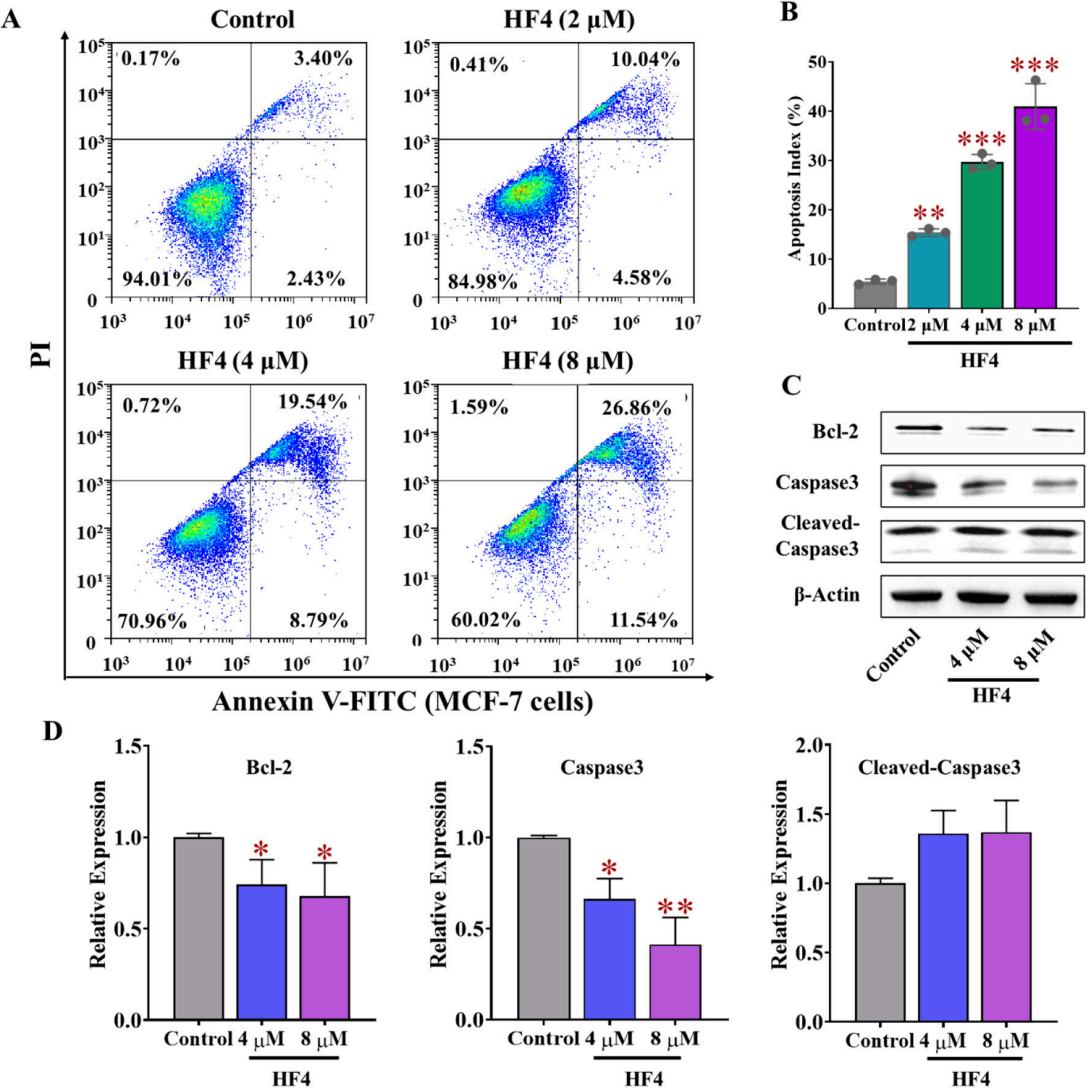
**HF4 promotes cell apoptosis in MCF-7 cells.** (A, B) The apoptotic impact of HF4 on MCF-7 cells at concentrations of 2 μM, 4 μM, and 8 μM was assessed using flow cytometry. (C, D) Western blot analysis was conducted to examine the expression levels of apoptosis-related proteins, namely, Bcl-2, Caspase-3, and Cleaved Caspase-3. Error bars represent the standard deviation; *p < 0.05, **p < 0.01, ***p < 0.001, in comparison to the control groups.

To elucidate the molecular mechanism by which HF4 inhibits breast cell growth, we assessed the impact of HF4 on MCF-7 cell apoptosis. As depicted in Figure 8A,B, the control group exhibited robust cell viability, with approximately 94% of cells remaining viable, indicative of healthy cell growth. Notably, the HF4 treatment group demonstrated a substantial increase in apoptotic cells, even at a low concentration of 2 μM. This effect displayed a dose-dependent pattern, suggesting that the HF4 treatment induced apoptosis. Furthermore, the expression of key apoptosis-related proteins (Bcl-2 and Caspase 3) was evaluated. As expected, HF4 significantly downregulated Bcl-2 and Caspase 3 expression, while upregulating cleaved Caspase 3 in a dose-dependent manner (Figure 8C,D). In summary, HF4 effectively induces apoptosis in breast cancer cells.

### HF4 Arrests the G1/S Transition and Affects the Expression Levels of Related Proteins

**Figure 9:**
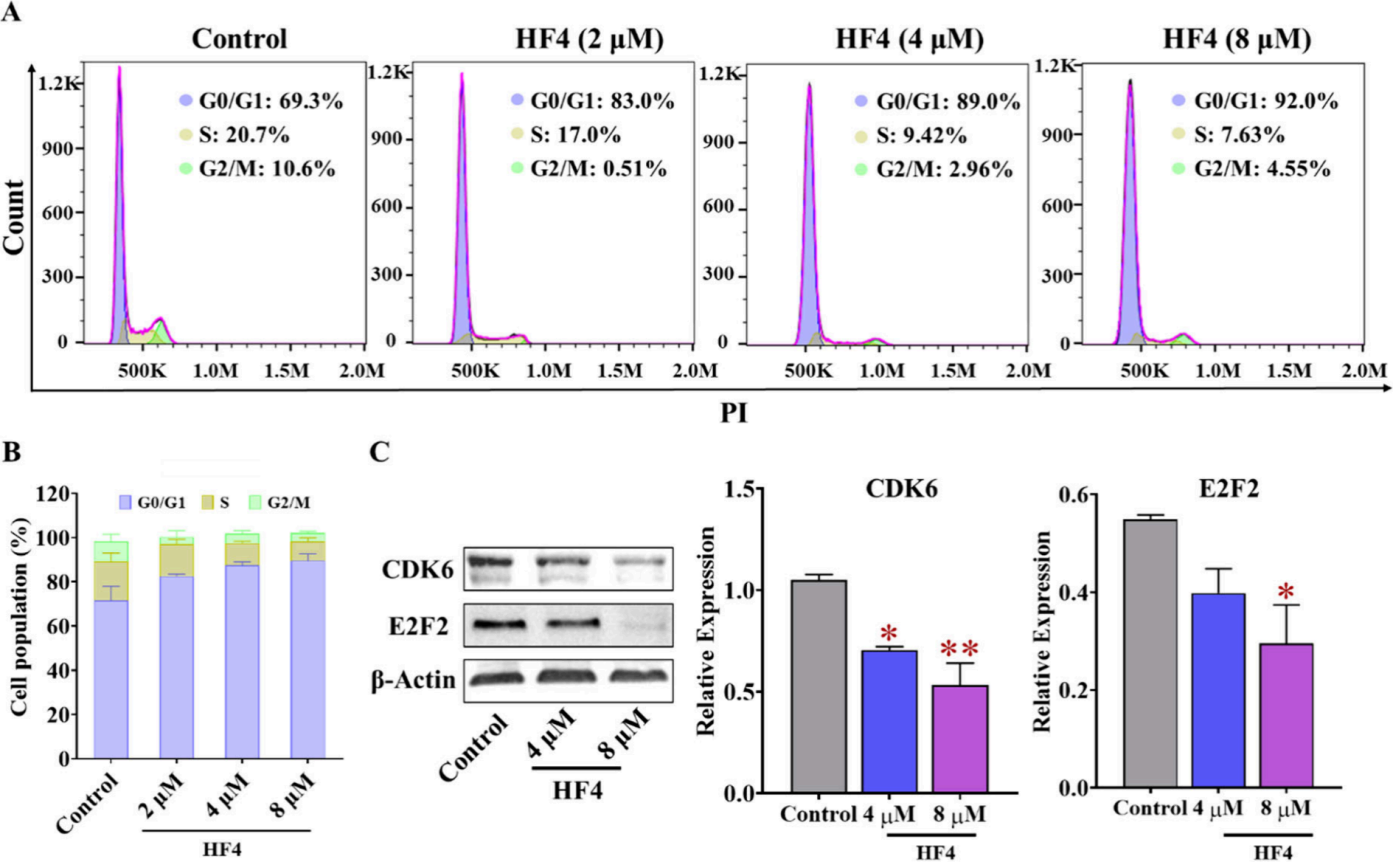
**HF4 arrests the G1/S transition in MCF-7 cells.** (A, B) The impact of HF4 on the cell cycle of MCF-7 cells was assessed at concentrations of 2 μM, 4 μM, and 8 μM using flow cytometry. (C) Western blot analysis was conducted to examine the levels of cell cycle-related proteins, including CDK6 and E2F2. Error bars represent the standard deviation; *p < 0.05, **p < 0.01, in comparison to the control groups.

BRD4 plays a crucial role in regulating transcription and epigenetic processes that govern cell cycle progression.^44^ Therefore, we conducted additional investigations to assess the impact of HF4 on the cell cycle in MCF-7 cells. In line with other known dual inhibitors of BRD4 and PARP1,^18^ HF4 impeded the transition from G1 to S phase and halted the cell cycle at the G0/G1 phase (Figure 9A,B). To confirm the effect of HF4 on the cell cycle, we examined the expression levels of key cell cycle-related proteins, including CDK6 and E2F transcription factor 2 (E2F2). Several investigations have consistently demonstrated that olaparib exerts a stimulatory effect on the expression of CDK6, a pivotal element in the G1/S transition phase, a phenomenon found to be unfavorable for the elimination of cancerous cells.^15^ Unlike olaparib, HF4 effectively and dose-dependently lowered CDK6 expression due to the synergistic inhibition of BRD4 and PARP1 (Figure 9C). Moreover, it suppressed E2F2 expression, leading to G1/S transition arrest and preventing the repair of DNA double-strand breaks induced solely by PARP1 inhibition.^15^

### HF4 Causes DNA Damage and Disrupts the Expression of Rad51

**Figure 10:**
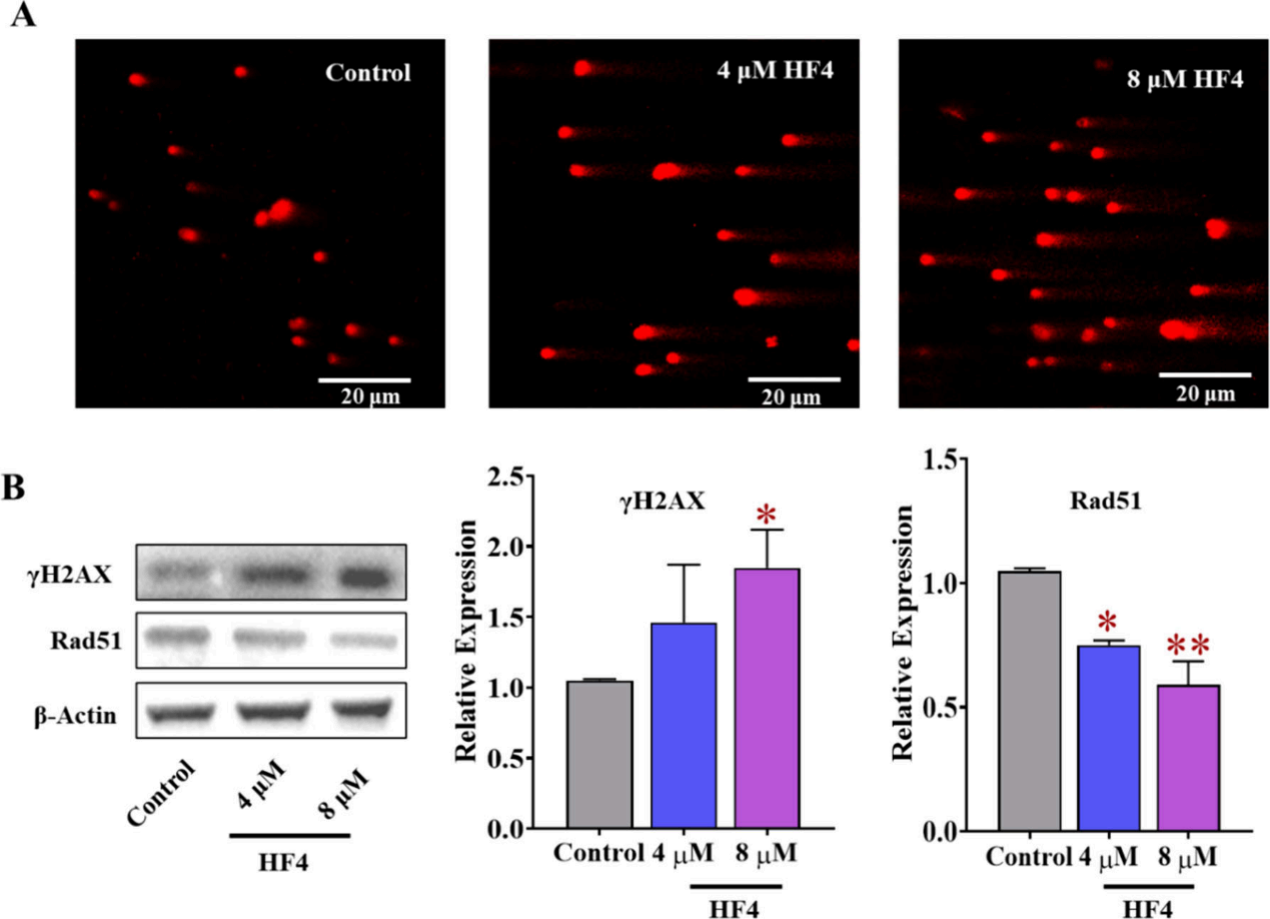
**HF4 causes DNA damage in MCF-7 cells.** (A) Comet images were captured using a confocal microscope following treatment with HF4 at concentrations of 4 and 8 μM on MCF-7 cells. (B) Western blot analysis was conducted to assess the levels of DNA damage-related proteins, such as γH2AX and Rad51. Standard deviation is represented by error bars, and statistical significance is denoted as follows: *p < 0.05, **p < 0.01 when compared to the control groups.

Apart from the cell cycle analysis, the impact of HF4 on DNA damage was assessed by using the comet assay. In comparison to the control group, an increase in both tail length and the percentage of damaged DNA was observed (Figure 10A), indicating the effective induction of DNA damage by HF4. Additionally, the expression of γH2AX, a protein associated with damaged DNA and positively correlated with the extent of DNA damage,^45^ was quantified. In line with the comet assay results, the levels of γH2AX exhibited dose-dependent upregulation (Figure 10B). Moreover, Rad51, a crucial factor contributing to homologous recombination (HR) repairment,^46^ was also investigated. As shown in Figure 10B, HF4 significantly reduced the expression of Rad51 in a dose-dependent manner, consequently hindering HR repair. These findings collectively underscore HF4’s capability to effectively induce DNA damage and impede HR repair. Taken together, our data-driven approach yielded an effective antitumor chemical entity.

### *In Silico* and Experimental Safety Assessment of HF4 on Normal Cell Lines

To further establish HF4’s advantage, we employed a web-based *in silico* cytotoxicity prediction tool (https://www.way2drug.com/clc-pred/index.php), encompassing the prediction against 391 tumor and 47 normal human cell lines,^47^ to predict the cytotoxicity of HF4 against nontumor cell lines. Applying a threshold of 0.4 (probability to be toxic against certain cell line), all predicted cell lines (eight in total) were identified as tumor cell lines, with MDA-MB-231 ranking highest, which is also validated in our experiments. The absence of nontumor cell lines among the top-ranked ones indicates a limited impact on normal cell line. When threshold of ≥0.15 was applied, among 50 predictions, only a single normal cell line (HEK293) was present in the list, with a predicted probability value below 0.2. We further reinforced the safety profile of HF4 by conducting experimental safety assessments on two normal cell lines. Illustrated in Figure S6, the IC_50_ values of HF4 for HEK293T (human embryonic kidney cell) and L02 (normal human liver cell) were 21.5 ± 7.4 μM and 26.3 ± 6.2 μM, respectively. Hence, the cytotoxicity of HF4 toward these two nontumor cells was notably lower compared to breast cancer cell lines. In summary, we conducted both computational safety predictions and experimental evaluations on normal cell lines to strengthen the assurance of HF4’s safety profile.

### Chemistry

**Scheme 1:**
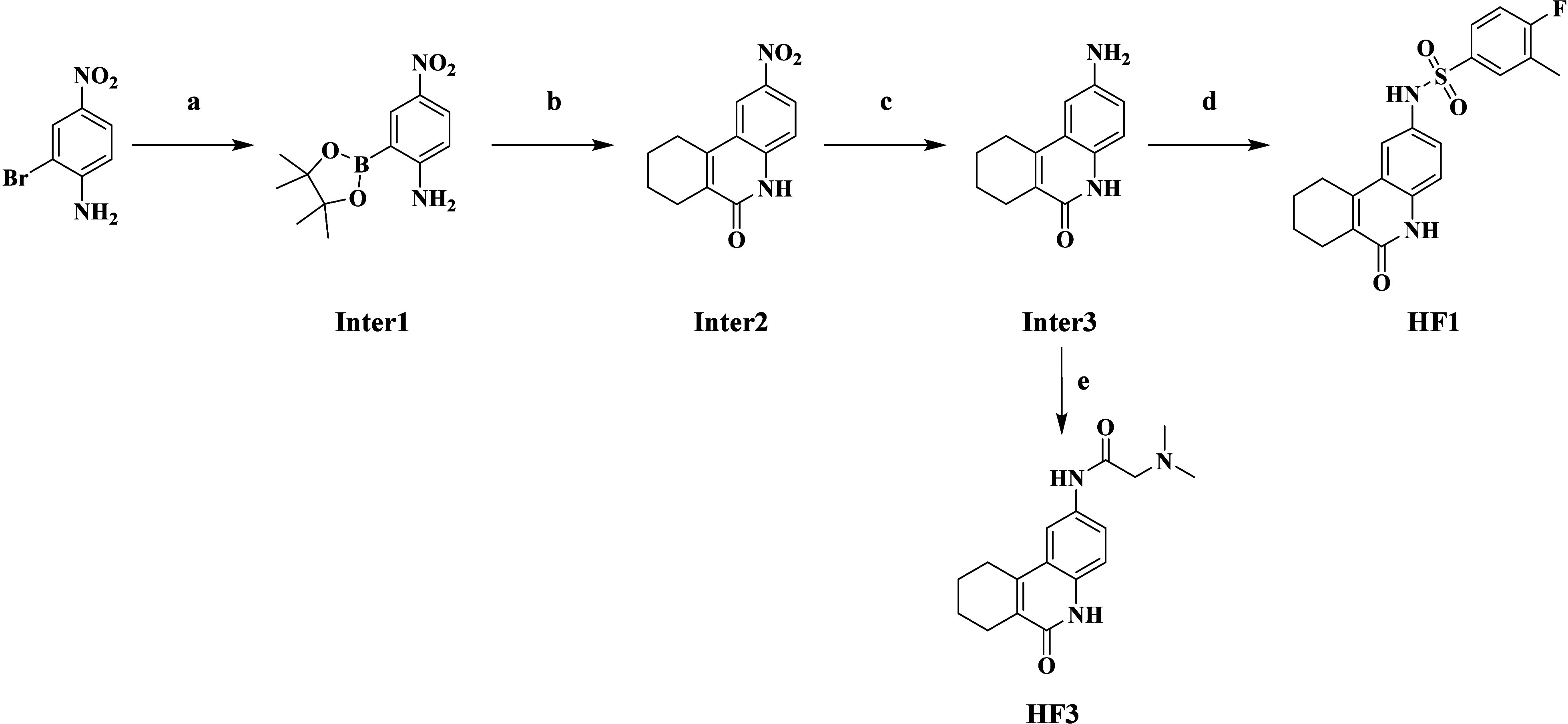
Synthetic Route of Compound HF1 and HF3 Reagents and conditions: (a) Pin_2_B_2_, Pd(dppf)Cl_2_, KOAc, DMF, 100 °C, 16 h; (b) ethyl 2-(((trifluoromethyl)sulfonyl)oxy)cyclohex-1-ene-1-carboxylate, K_2_CO_3_, Pd(dppf)Cl_2_, dioxane, H_2_O, 80 °C, 3 h; (c) Fe/NH_4_Cl, EtOH, H_2_O, 55–90 °C, 1.5 h; (d) 4-fluoro-3-methylbenzenesulfonyl chloride, Py, 25 °C, 2 h; (e) dimethylglycine, DCC, DMAP, DCM, 25 °C, 2 h.

The synthetic routes for our designed compounds (HF1-HF5) are detailed in Schemes 1 and 3. To produce compound HF1 (Scheme 1), the process began with the synthesis of borate ester Inter1. This was achieved by reacting bis(pinacolato)diboron with the commercially available material 2-bromo-4-nitroaniline through the Miyaura Borylation Reaction. Subsequently, Inter2, featuring a tricyclic ring scaffold, was obtained by the reaction of Inter1 with ethyl 2-(((trifluoromethyl)sulfonyl)oxy)cyclohex-1-ene-1-carboxylate, which was then reduced to an amine to yield Inter3. The substituted aniline reacted with sulfuryl chloride and carboxylic acid to synthesize the target compounds HF1 and HF3, respectively.

**Scheme 2:**
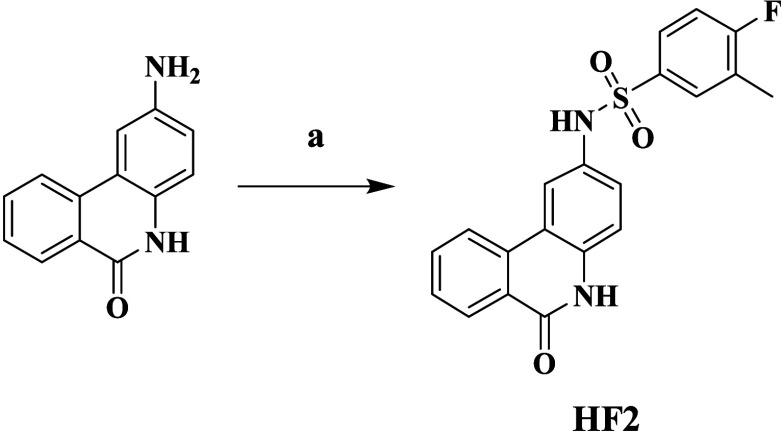
Synthetic Route of Compound HF2 Reagents and conditions: (a) 4-fluoro-3-methylbenzenesulfonyl chloride, Py, 25 °C, 2 h.

Compound HF2 was produced through the reaction of 2-aminophenanthridin-6(5*H*)-one with 4-fluoro-3-methylbenzenesulfonyl chloride under ambient conditions, as outlined in Scheme 2.

**Scheme 3:**
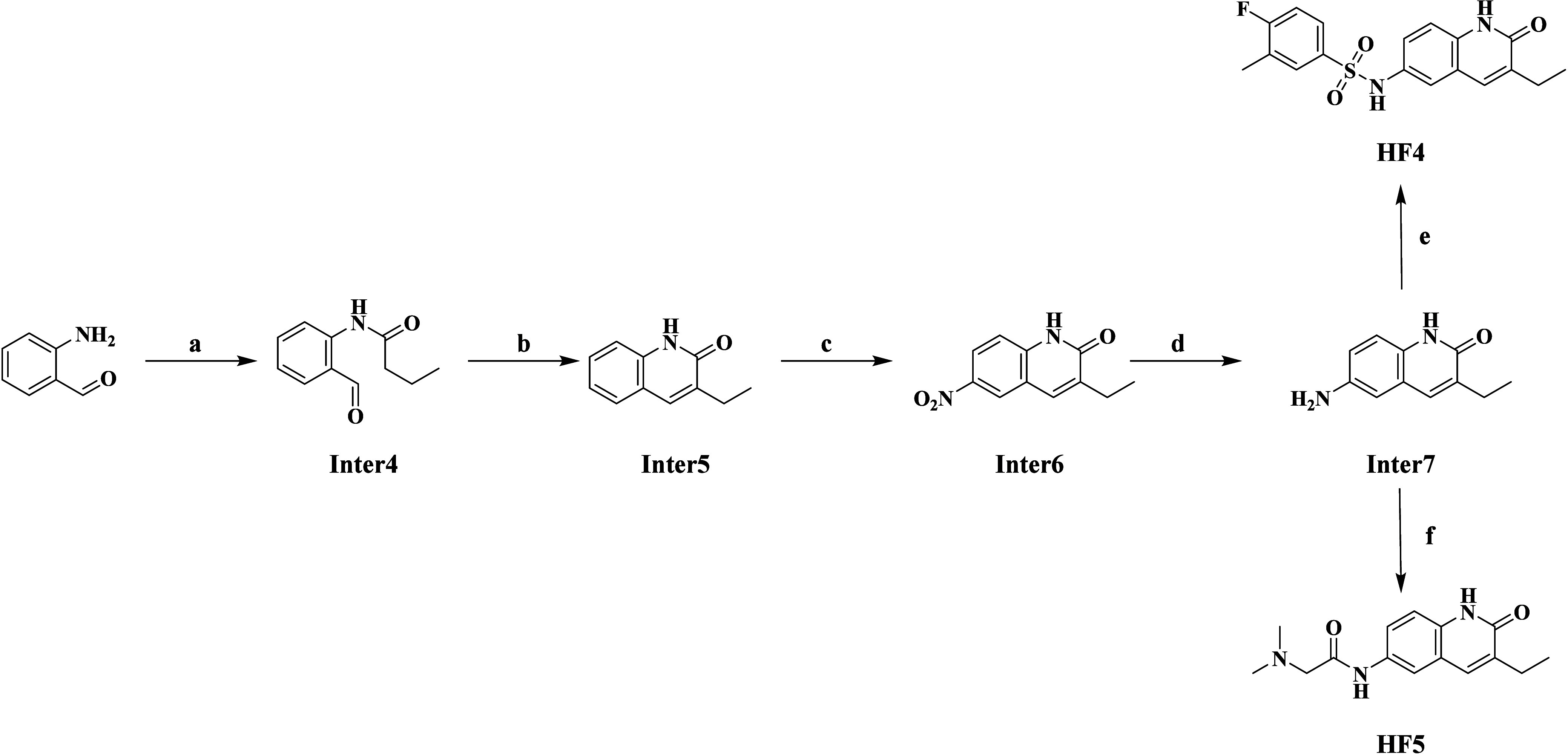
Synthetic Route of Compound HF4 and HF5 Reagents and conditions: (a) butyryl chloride, Py, THF, 0–25 °C, 2 h; (b) Cs_2_CO_3_, DMF, 80 °C, 16 h; (c) H_2_SO_4_, HNO_3_, 0 °C, 2 h; (d) Fe/NH_4_Cl, EtOH, H_2_O, 80 °C, 4 h; (e) 4-fluoro-3-methylbenzenesulfonyl chloride, Py, 25 °C, 3 h; (f) dimethylglycine, HATU, DIEA, DMF, 25 °C, 2 h.

To synthesize Compounds HF4 and HF5 (summarized in Scheme 3), 2-aminobenzaldehyde initially underwent amidation with butyryl chloride, yielding Inter4. This intermediate was subsequently cyclized to form Inter5 with a bicyclic ring structure in the presence of cesium carbonate. Next, Inter6 with a nitro group was produced through nitration and further reduced to yield Inter7 with an amine functional group. Compound HF4 was then obtained by reacting 4-fluoro-3-methylbenzenesulfonyl chloride with Inter7. Additionally, HF5 was obtained by reacting Inter7 with dimethylglycine.

## Conclusions

Multitarget compounds will continue to gain momentum in the treatment of complex multifactorial diseases. Nevertheless, the systematic design of compounds with desired drug-like properties remains a challenging endeavor. In this context, we leveraged available bioactivity data associated with PARP1 and BRD4 and employed cheminformatics tools to identify amide-based structural components, thereby enabling us to establish a good starting point for the dual-target compound design and be able to rapidly optimize dual-target activity. Among several designed compounds, HF4 emerges as the promising candidate due to its unbulky structural features, demonstrating single-digit antiproliferative activity against both BRCA-deficient and proficient breast cancer cell lines. Mechanistically, HF4 hinders the growth of breast cancer cells by inducing cell cycle arrest and causing DNA damage. In summary, our study highlights our data-driven efforts in designing inhibitors for PARP1 and BRD4 with a focus on HF4, which exhibits strong antiproliferative activity in cells, warranting further exploration and optimization.

## Materials and Methods

### General Synthesis Procedures and Description

^1^H NMR spectra were recorded at 400 MHz. The chemical shifts were recorded in parts per million relative to tetramethylsilane and with the solvent resonance as the internal standard. Deuterated reagents used deuterated dimethyl sulfoxide. Data were reported as follows: chemical shift. Multiplicity (s = singlet, d = doublet, t = triplet, m = multiplet), coupling constants (Hz), integration was collected at 400 MHz with complete proton decoupling. ^13^C NMR data were collected at 101 MHz with complete proton decoupling. Chemical shifts were reported in ppm from the tetramethylsilane with the solvent resonance as the internal standard. Deuterated reagents used deuterated dimethyl sulfoxide. High-resolution mass spectra were recorded with a Thermo Scientific LT1-Orbitrap. All chemicals were obtained from commercial sources and used without further purification. The Purification of final compounds was carried out by prep-HPLC (Boston Prime C18 150 × 30 mm × 5um). The purity of targeted compounds was determined to be >95% by reverse-phase high-performance liquid chromatography (HPLC) analysis. HPLC instrument: Shimadzu HPLC (Ultimate XB-C18,3 um 3.0 × 50 mm). Elution: MeCN in water; flow rate: 1.2 mL/min. High-performance liquid chromatography–mass spectrometry (HPLC-MS) was performed on a Shimadzu LC20-MS2010. The synthesis procedures for all compounds and NMR data were provided in Supporting Information.

### Bioactivity Data Curation

Known inhibitors targeting PARP1 and BRD4 were collected from two major repositories, ChEMBL^48^ and BindingDB,^36^ retrieved in October 2022, both of which offer publicly accessible chemical and biological data. To ensure data quality and reliability, the following criteria were applied: (a) only compounds tested against the human PARP1 protein (ChEMBL ID: CHEMBL3105) and BRD4 protein (ChEMBL ID: CHEMBL1163125) were included; (b) the chemical structures of molecules were standardized, involving salt/minor component removal, tautomerization, and charge neutralization. This standardization process was conducted using the canSARchem registration workflow,^49^ an open-access pipeline; (c) for potency measurements, *K*_*i*_, *K*_*d*_, *IC*_*50*_, and *EC*_*50*_ with were considered; (d) potency measurements that used approximate symbols like “>” or “∼” were omitted from the analysis, while those employing “=” and “<” were included; and (e) potency values were converted to their negative logarithmic form (pPOT). In cases where a compound had multiple potency values, the highest among them was employed as the final potency annotation. In the current analysis, only the compounds with pPOT ≥ 5 (10 μM) were kept for further analysis.

### Experimental Structural Data of PARP1 and BRD4 in PDBe-KB

Using the UniProt ID for the PARP1 protein (P09874) and the BRD4 protein (O60885), we retrieved ligands with structural information from the Protein Data Bank (PDB) in Europe Knowledge Base (PDBe-KB) at https://www.ebi.ac.uk/pdbe/pdbe-kb/. Initially, we obtained a total of 87 PDB structures for PARP1 and 490 PDB structures for BRD4. Subsequently, we carefully examined these structural data sets to identify the presence of small molecules. Apo structures were excluded from the analysis. The chemical structures of the ligands were then standardized using the same cleaning pipeline, i.e., canSARchem registration workflow.^49^ This process resulted in 69 ligands for PARP1 and 449 ligands for BRD4. These structural ligands were additionally subjected to compound fragmentation (see below), enabling the detection of crucial structural elements pertaining to key pharmacophores for structure-based compound optimization.

### Amide-Containing Pharmacophore Identification

In this study, two distinct pharmacophores: one involving the primary amide directly attached to ring systems (e.g., amide-containing scaffold in niraparib) and the other involving lactam structures (e.g., amide scaffold of olaparib) were considered. To identify structural components with embedded amides, a search for exocyclic single bonds within each molecule (including the PDB ligand) was conducted. Simultaneous cleavage occurred for all exocyclic single bonds within the molecules, except for the single bond directly connecting the primary amide to the ring system. Compound fragmentation was performed using the RDKit toolkit.^39^ Following compound fragmentation, only scaffolds featuring amide functional groups were preserved. To circumvent the inclusion of a “bulky pharmacophore”, only bi- or tricyclic ring systems were taken into consideration. Additionally, only scaffolds with a minimum of five bioactive compounds were retained. Accordingly, amide-containing scaffolds were gathered separately for PARP1 and BRD4, and these were utilized to detect the shared key components, serving as the starting points for the design of compounds with dual-target activity profiles.

### Network Analysis between Target and Scaffold

The scaffold and target relationships were systematically structured in a network-based format. In this format, nodes represent scaffolds and targets. A target node was linked to a scaffold node if its parent compound possessed an annotated activity against that specific target. Edges were shaded purple when at least one parent compound had been crystallized with the corresponding target and gray otherwise. The network visualization was generated using Cytoscape software (version 3.9.1).^50^

### Molecular Docking

Docking analysis was conducted using MOE software (version 2020.09, https://www.chemcomp.com), utilizing the resolved PJ34-PARP1 complex (PDB code: 4UXB) and PFI-1 in complex with BRD4 (PDB code: 4E96) as templates.^13,51^ Before docking, the crystal structures were corrected for missing atoms and bonds and protonated under physiological conditions using standard MOE methods, and water molecules were removed. The active site was defined based on the cocrystallized ligands (PJ34 or PFI-1), and other default docking parameters were applied. Initially, all ligands were assessed using the London dG function, and 50 conformations were retained for each molecule. Following this, the GBVI/WSA dG function was employed for secondary scoring, and only the conformation with the best score was preserved.

### *In Vitro* PARP1 Enzymatic Activity Measurement

The inhibition of the tested compounds on PARP1 enzymatic activity was determined by ELISA in 96-well plates. Each well was precoated with histone (20 μg/mL) diluted in 100 μL of PBS buffer (10 mM NaH_2_PO_4_, 100 mM Na_2_HPO_4_, 150 mM NaCl, pH 7.4) by incubation at 4 °C overnight. 100 μM NAD^+^, 25 μM biotinylated NAD^+^, and 200 nM slDNA diluted in 30 μL of reaction buffer (50 mM Tris, 2 mM MgCl_2_, pH 8.0) were added into each well, and then 5 μL of compound or solvent control was added at varying concentrations. The reaction was initiated by the addition of 20 μL of PARP1 (50 ng/well) at 30 °C for 1 h. Then the reaction solution was added to 50 μL of streptavidin conjugated HRP. The assay was performed at 30 °C for an additional 30 min. Finally, 100 μL of solution (H_2_O_2_ and luminol in citrate buffer 0.1 M, pH 5.4) was added and luminescent signal was measured using a multiwell spectrophotometer (Molecular Devices SpectraMax M5 microplate reader). The inhibition rate of PARP1 enzymatic activity was calculated as (Lu control – Lu treated/Lu control) × 100%. The concentration required for 50% inhibition of PARP1 enzymatic activity (IC_50_) was calculated using nonlinear regression with normalized dose–response fit using Prism GraphPad software.

### *In Vitro* BRD Enzymatic Inhibitory Activity Measurement

The assay was performed by TR-FRET technology using a BRD and its corresponding ligand (BET). The TR-FRET signal from the assay is correlated with the amount of ligand binding to the bromodomain. All of the binding reactions were conducted at room temperature. The 20 μL reaction mixture in Assay Buffer contains either bromodomains, BET Ligand, and the indicated amount of inhibitor. For the negative control (blank), 5 μL of the assay buffer was added instead of the BET ligand. The reaction mixture was incubated for 120 min. After the incubation with the ligand, the TR-FRET signal was measured using a Tecan Infinite M1000 plate reader.

### Cell Culture, Antibodies, and Reagents

The breast cancer cell lines, namely, MDA-MB-231, MDA-MB-436, and MCF-7, were procured from the Cell Resources Center at the Shanghai Academy of Life Sciences in Shanghai, China. These cell lines were maintained in Dulbecco’s Modified Eagle Medium (DMEM) supplemented with 10% fetal bovine serum, and they were cultured in a controlled environment with 5% CO_2_ at a temperature of 37 °C.

In this study, a range of antibodies was utilized, all of which were sourced from Abcam. These antibodies included anti-BRD4, anti-PARP1, anti-Bcl, anticaspase 3, anticleaved-caspase 3, anti-CDK6, anti-E2F2, anti-γH2AX, and anti-Rad51. Additionally, several assay kits were employed in the research. The MTT assay and comet assay kit were acquired from Beyotime Biotechnology, while the cell apoptosis detection kit and cell cycle detection kit were obtained from KeyGEN Biotech.

### Cell Viability Assay

MDA-MB-231, MDA-MB-436, and MCF-7 cells were seeded into 96-well plates with the number of 5 × 10^3^ in each cell and allowed to incubate for 24 h. Subsequently, various concentrations of the test compounds (ranging from 100 to 0.78 μM with twice gradient dilution) were introduced. After 4 days of incubation, MTT solution was applied, and the cells were incubated for an additional 4 h. Following the removal of the supernatant, the violet crystals were dissolved in 200 μL of DMSO, and the absorbance was measured at 570 nm.

### Colony Formation Assay

The cells were placed in a 6-well plate at a density of approximately 500 cells per well and allowed to incubate for 24 h. Subsequently, the cells were exposed to the specified concentrations of test compounds and a control vehicle for a duration of 7 days. Following this treatment period, the cells were fixed using a 4% formaldehyde solution, stained with crystal violet, and then imaged.

### Cell Cycle Detection

Cell cycle analysis was performed using a PI cell cycle detection kit. Following a 24 h treatment, cells were gathered, fixed with 75% ethanol, and stained with PI according to the manufacturer’s protocol. Subsequently, cell cycle analysis was detected using BD FACSCelesta flow cytometry (New York, USA). The obtained data were then subjected to analysis using FlowJo_V10.

### Cell Apoptosis Detection

Cell apoptosis was assessed using the Annexin V-FITC/PI apoptosis detection kit. Following a 24 h treatment, cells were harvested and rinsed with PBS. Subsequently, the gathered cells were stained in accordance with the manufacturer’s guidelines and analyzed using BD FACSCelesta flow cytometry (New York, USA). FlowJo_V10 software was employed for data analysis.

### Alkaline Comet Assay

The Alkaline comet assay was executed in accordance with the manufacturer’s instructions. Following a 48 h treatment period, cells were harvested and combined with low melting point agarose. The resulting mixture was layered onto slides coated with agarose as a normal melting point agarose. Subsequently, the cells on the slides were lysed overnight at 4 °C and then unwound in an alkaline unwinding solution for 30 min at room temperature. Following electrophoresis, the samples were neutralized and stained with propidium iodide. The outcomes were visualized using a confocal microscope (Nikon A1 HD25), with three random images captured from each slide.

### Western Blot Analysis

Following a 48 h treatment, cellular proteins were extracted using RIPA cell lysis buffer containing protease and phosphatase inhibitor cocktail. The protein concentration was assessed by using a BCA assay. Subsequently, equal amounts of these proteins were loaded onto an SDS-PAGE gel and transferred onto a PVDF membrane. The protein-loaded membrane was then blocked using a commercial blocking solution (Beyotime, China) and sequentially incubated with primary and secondary antibodies. Protein bands were visualized using an ECL chemiluminescence kit and quantified using ImageJ software.

### Statistical Analysis

Statistical analysis was conducted using GraphPad Prism 8 (GraphPad Software, Inc., La Jolla, CA, USA). All statistical results are presented as mean ± standard deviation (SD) for a sample size of n = 3, unless otherwise stated. Multiple group comparisons were carried out using analysis of variance (ANOVA). Statistical significance was defined as a *p*-value less than 0.05.

## Data Availability

The bioactivity data sets essential for reproducing the findings in this paper are freely available at https://www.ebi.ac.uk/chembl/ and https://www.bindingdb.org/rwd/bind/index.jsp. Furthermore, experimental data generated during this study is provided in the Supporting Information files.

## Supporting Information Available

^1^H NMR, ^13^C NMR, LC-MS, HRMS spectra, and HPLC purity plots of target compounds (PDF)

## Abbreviations

PARP1: poly(ADP-ribose) polymerase 1
BRD4: bromodomain-containing protein 4
FDA: U.S. Food and Drug Administration
BD: bromodomains
BET: bromodomain and extra-terminal domain
BRCA: breast cancer gene
TOPBP1: DNA topoisomerase 2-binding protein 1
Bcl-2: B-cell lymphoma-2
CDK6: cyclin-dependent kinase 6
E2F2: E2F transcription factor 2
HR: homologous recombination
NAD^+^: nicotinamide adenine dinucleotide
PI: propidium iodide
HAC: heavy atom count
MW: molecular weight
SAR: structure–activity relationship
LEI: ligand efficiency index
