# Supplementary material for: Combining Data-Driven and Structure-Based Approaches in Designing Dual PARP1-BRD4 Inhibitors for Breast Cancer Treatment
Source: J Chem Inf Model. 2024 Sep 18;64(19):7725–42. doi: 10.1021/acs.jcim.4c01421 (PMC11480993; doi:10.1021/acs.jcim.4c01421)
Supplement: Supplementary file 1 — ci4c01421_si_001.pdf [file ci4c01421_si_001.pdf]

Supplementary Information

Combining data-driven and structure-based approaches in designing dual PARP1-BRD4 inhibitors for breast cancer treatment

Bo Feng[1], Hui Yu[2], Xu Dong[1], Alejandro Díaz-Holguín[3],

Albert A. Antolin[4,5], Huabin Hu[3,4]*

[1] Department of Pharmacy, The Affiliated Hospital of Yangzhou University, Yangzhou University, Yangzhou, 225000, P. R. China

[2] Information School, University of Sheffield, 211 Portobello, Sheffield, S1 4DP, UK

[3] Science for Life Laboratory, Department of Cell and Molecular Biology, Uppsala University, BMC, Box 596, SE-751 24, Uppsala, Sweden

[4] Centre for Cancer Drug Discovery, Division of Cancer Therapeutics, The Institute of Cancer Research, London, UK

[5] ProCURE, Catalan Institute of Oncology, Oncobell, Bellvitge Institute for Biomedical Research (IDIBELL), L'Hospitalet del Llobregat, Barcelona, Catalonia, Spain

* Correspondence: huabinhu11@outlook.com (Huabin Hu)

Contents

Top ranked non-amide-based scaffolds (bicyclic/tricyclic) in BRD4 .... S3

Exemplary bioactive BRD4/PARP1 inhibitors that share amide scaffolds S4

General information of synthesized and reference compounds S5

Predicted binding modes of HF4 in PARP1 and BRD4 .... S6

Anti-proliferative effects of HF1 on breast cancer cell lines S6

Cytotoxicity of HF4 against non-tumor cells S7

General synthetic experimental conditions S8

^{1}H NMR, ^{13}C NMR, LC-MS, HRMS, and HPLC spectra for designed compounds S16

- **4-fluoro-3-methyl-N-(6-oxo-5,6,7,8,9,10-hexahydrophenanthridin-2-yl)benzenesulfonamide (HF1)** ... S19
- **2-((2-(dimethylamino)ethyl)amino)-7,8,9,10-tetrahydrophenanthridin-6(5H)-one (HF2)** S22
- **2-(dimethylamino)-N-(6-oxo-5,6-dihydrophenanthridin-2-yl)acetamide (PJ34)** S25
- **2-(dimethylamino)-N-(6-oxo-5,6,7,8,9,10-hexahydrophenanthridin-2-yl)acetamide (HF3)** S27
- **N-(3-ethyl-2-oxo-1,2-dihydroquinolin-6-yl)-4-fluoro-3-methylbenzenesulfonamide (HF4)** S34
- **2-(dimethylamino)-N-(3-ethyl-2-oxo-1,2-dihydroquinolin-6-yl)acetamide (HF5)** S37

Top ranked non-amide-based scaffolds (bicyclic/tricyclic) in BRD4

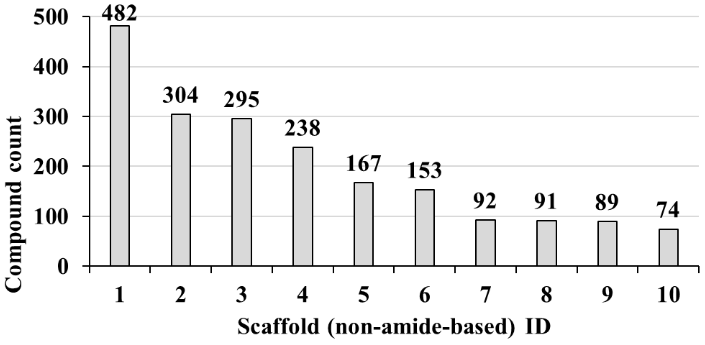

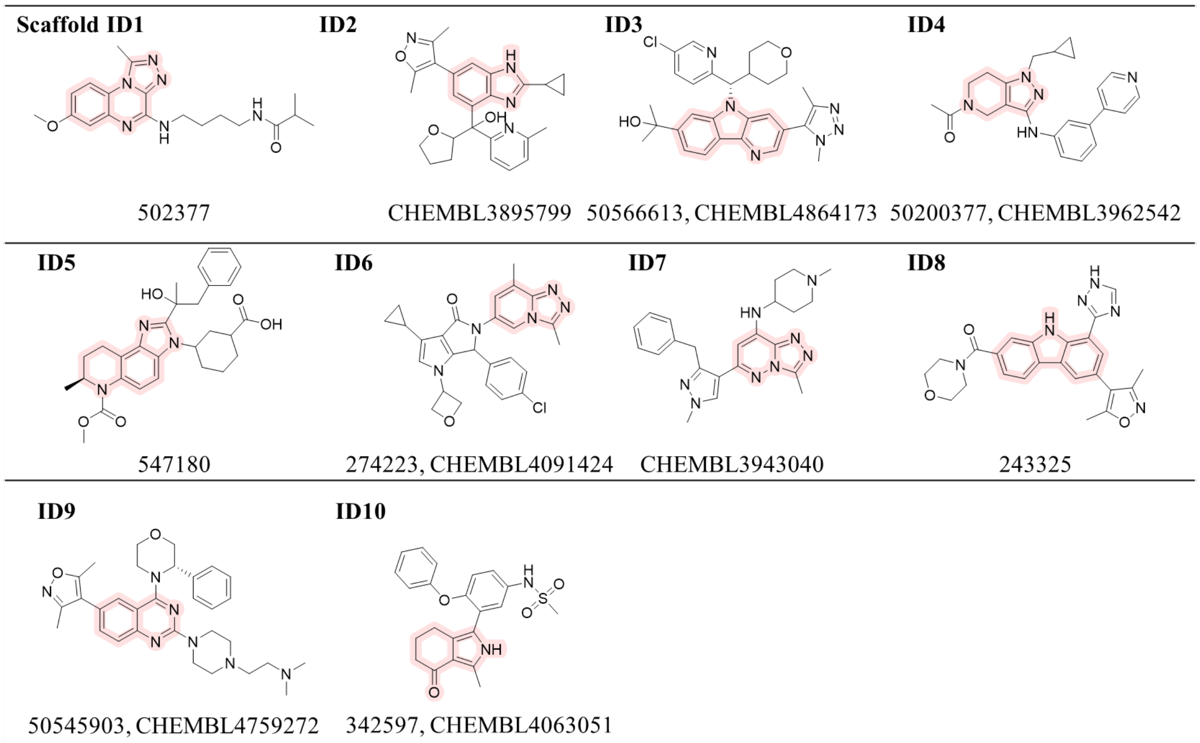

Figure S1. The top 10 non-amide-based scaffolds identified in BRD4 inhibitors. For each scaffold, an exemplary BRD4 compound is shown, with the scaffold highlighted in light red and the compound ID displayed below each structure.

Exemplary bioactive BRD4/PARP1 inhibitors that share amide scaffolds

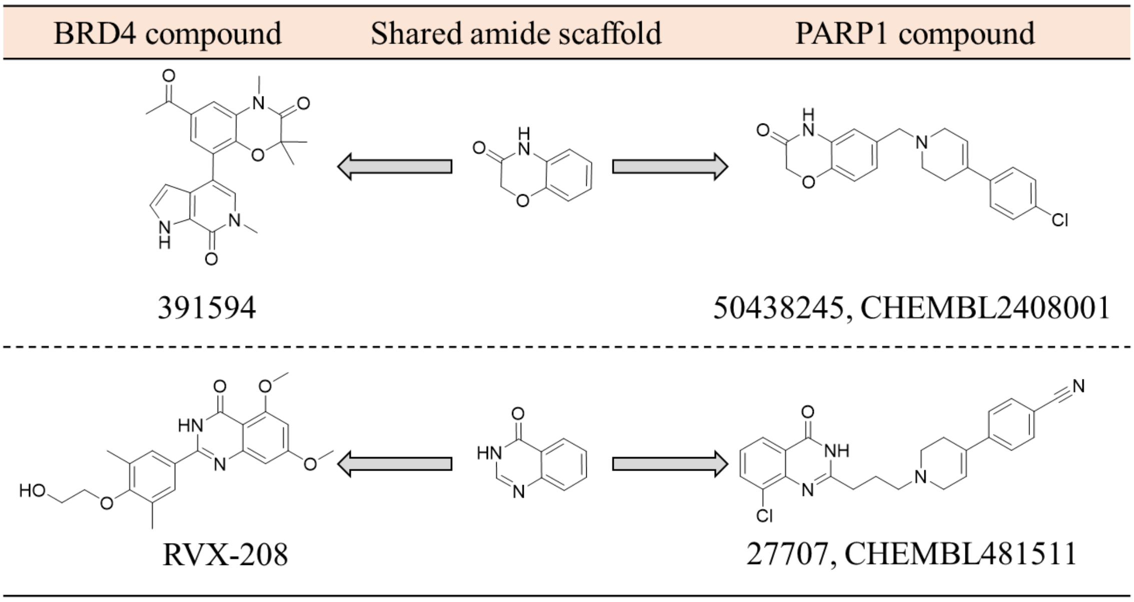

Figure S2. Exemplary bioactive BRD4/PARP1 inhibitors that share amide scaffolds. Two pairs of compounds with shared scaffolds are presented, each accompanied by their BindingDB and/or ChEMBL ID.

General information of synthesized and reference compounds

Structures	cLogP	IC_{50} (nM)		Docking score (kcal/mol)	
		PARP1	BRD4	PARP1	BRD4
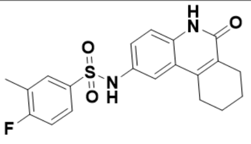 HF1	3.66	94 ± 21	3560 ± 951	-8.19	-6.70
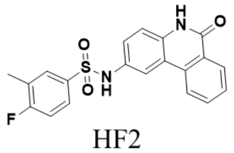 HF2	3.93	339 ± 86	32635 ± 6341	-7.81	-6.29
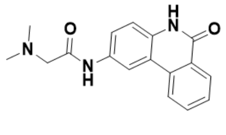 PJ34	2.18	8 ± 2	2072 ± 588	-7.78	-6.60
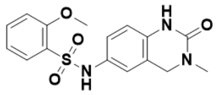 PFI-1	2.47	10% (10 μM)	220	-6.15	-7.28
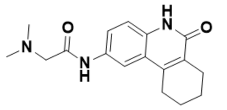 HF3	1.91	16 ± 6	28805 ± 6987	-7.98	-6.45
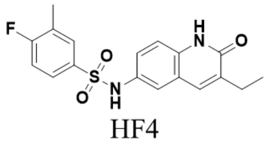 HF4	3.34	2019 ± 683	1210 ± 324	-7.51	-7.27
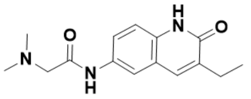 HF5	1.59	736 ± 234	34304 ± 7903	-6.64	-6.92

Figure S3. General information of synthesized and reference compounds.

Predicted binding modes of HF4 in PARP1 and BRD4

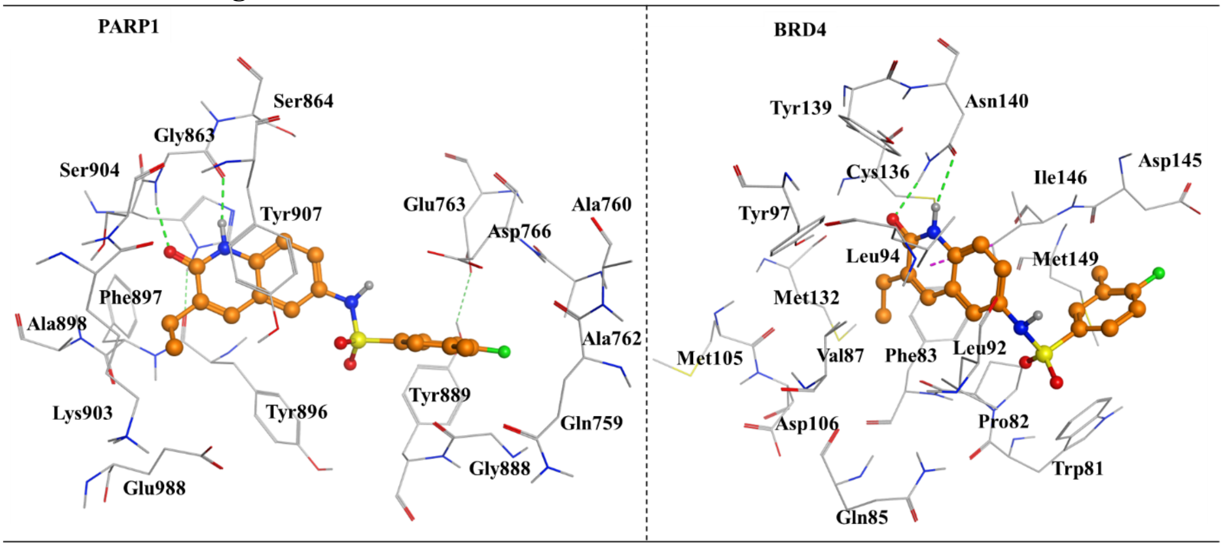

Figure S4. Predicted binding mode of HF4 in PARP1 (PDB code: 4UXB) and BRD4 (PDB code: 4E96). The 3D structure of the binding site displays protein carbons in gray and ligand carbons in orange. Hydrogen bonds are depicted as green dashed lines, with hydrogen-π interactions highlighted in magenta.

Anti-proliferative effects of HF1 on breast cancer cell lines

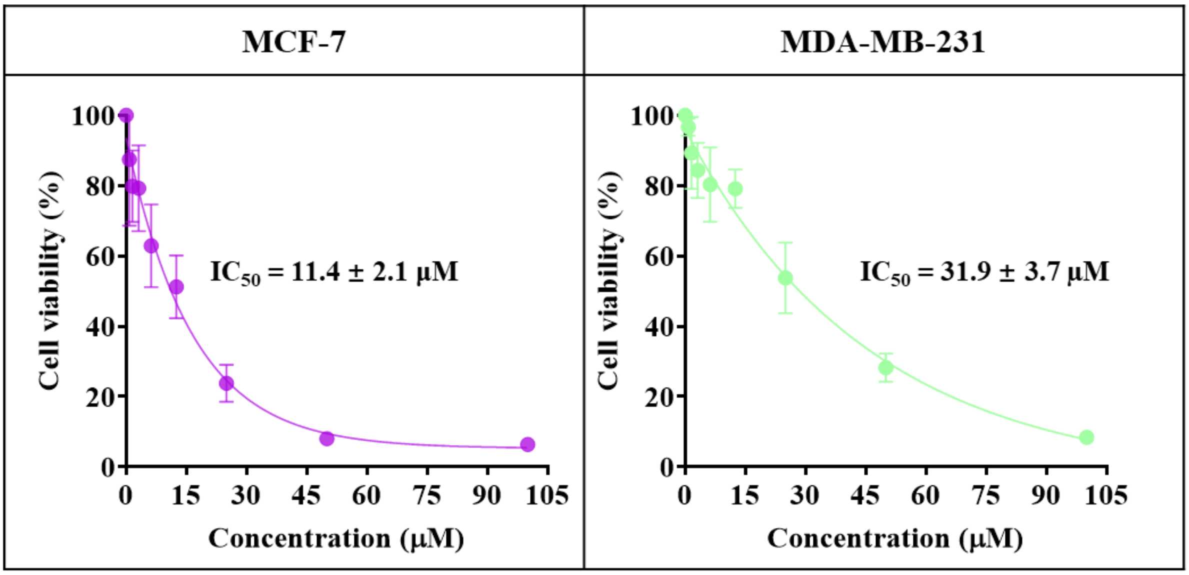

Figure S5. The antitumor effectiveness of HF1 was evaluated in MCF-7 and MDA-MB-231 cells (breast cancer cells with wild-type BRCA) during a 4-day treatment.

Cytotoxicity of HF4 against non-tumor cells

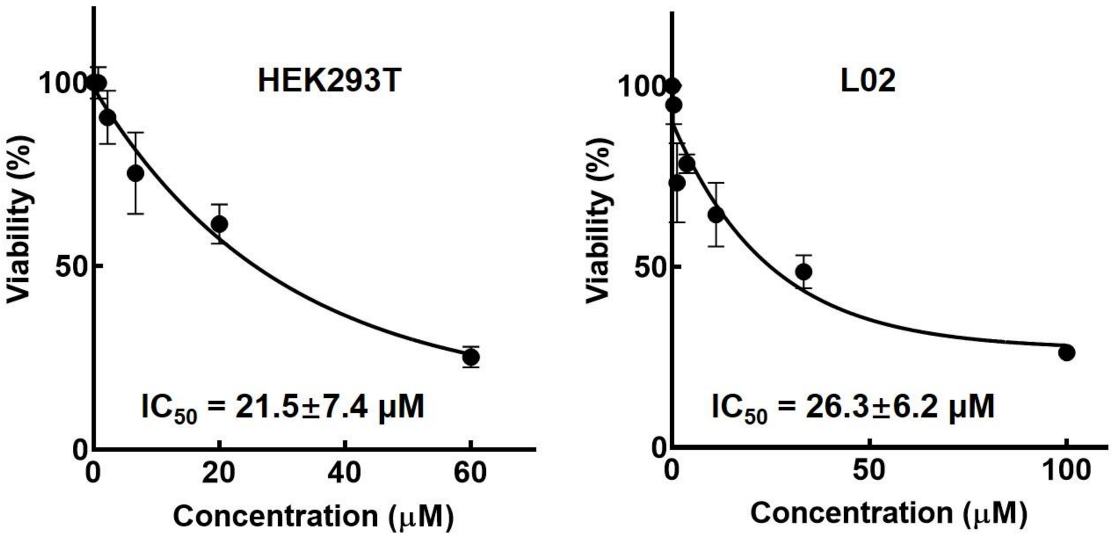

Figure S6. Cytotoxicity of HF4 against non-tumor cells during a 4-day treatment.

General synthetic experimental conditions

4-nitro-2-(4,4,5,5-tetramethyl-1,3,2-dioxaborolan-2-yl)aniline ***(Inter1)***. To a suspension of 2-bromo-4-nitroaniline (5.00 g, 23.04 mmol, 1.00 *eq*) and KOAc (5.65 g, 57.6 mmol, 2.50 *eq*) in DMF (100 mL) was added Pin_2B_2 (11.7 g, 46.1 mmol, 2.00 *eq*), and Pd(dppf)Cl_2 (1.69 g, 2.30 mmol, 0.1 *eq*). The mixture was stirred at 100 °C for 16 h. The residue was diluted with H_2O 500 mL and extracted with EtOAc 600 mL (200 mL × 3). The combined organic layers were washed with brine 600 mL (300 mL × 2), dried over $MgSO_4$, filtered and concentrated under reduced pressure to give a residue. The residue was purified by flash silica gel chromatography (ISCO®; 80 g SepaFlash® Silica Flash Column, Eluent of 0 ~ 15% Ethyl acetate/Petroleum ethergradient @ 100 mL/min). Compound **Inter1** (660 mg, 2.50 mmol, 10.8% yield, 100% purity) was obtained as a light yellow solid. ^1H NMR (400 MHz, $CDCl_3$), δ ppm: 8.54 (d, *J* = 2.6 Hz, 1H), 8.08 (dd, *J* = 2.6, 9.0 Hz, 1H), 6.53 (d, *J* = 9.0 Hz, 1H), 5.51 (br s, 2H), 1.35 (s, 12H). LC-MS (ESI) *m/z* 265.1 $[M+H]^+$.

2-nitro-7,8,9,10-tetrahydrophenanthridin-6(5H)-one ***(Inter2).*** To a mixture of **Inter1** (343 mg, 1.14 mmol, 1.00 *eq*), ethyl 2-(((trifluoromethyl)sulfonyl)oxy)cyclohex-1-ene-1-carboxylate (300 mg, 1.14 mmol, 1.00 *eq*) and K_2CO_3 (392 mg, 2.84 mmol, 2.50 *eq*) in dioxane (3.00 mL) and H_2O (0.600 mL) was added Pd(dppf)Cl_2 (41.6 mg, 56.8 μmol, 0.0500 *eq*) under N_2 atmosphere. The mixture was degassed and purged with N_2 for 3 times, and then the mixture was stirred at 80 °C for 3 h under N_2 atmosphere. The mixture was diluted with H_2O (50 mL) and extracted with EtOAc (20 mL × 3). The combined organic layers were washed with brine (30 mL × 2), dried over $MgSO_4$, filtered and concentrated under reduced pressure to give a residue. The residue was purified by flash silica gel chromatography (ISCO®; 4 g SepaFlash®

Silica Flash Column, Eluent of 60% EtOAc/Petroleum ethergradient @ 50 mL/min). Compound **Inter2** (33.0 mg, 133 μmol, 11.7% yield) was obtained as a brown solid. ^{1}H NMR (400 MHz, DMSO-d_6), δ ppm: 12.23 (br s, 1H), 8.51 (d, J = 2.0 Hz, 1H), 8.31 (dd, J = 2.1, 8.7 Hz, 1H), 7.44 (d, J = 9.2 Hz, 1H), 2.90 (br d, J = 5.9 Hz, 2H), 2.62 - 2.55 (m, 2H), 1.86 - 1.68 (m, 4H). LC-MS (ESI) *m/z* 245.0 $[M+H]^+$.

2-amino-7,8,9,10-tetrahydrophenanthridin-6(5H)-one ***(Inter3).*** To a solution of **Inter2** (160 mg, 655 μmol, 1.00 *eq*) and Fe (183.0 mg, 3.28 mmol, 5.00 *eq*) in EtOH (1.00 mL) was added a solution of NH_4Cl (175 mg, 3.28 mmol, 5.00 *eq*) in H_2O (0.500 mL) at 55 °C. The mixture was stirred at 90 °C for 1.5 h. The residue was diluted with H_2O (30 mL) and extracted with DCM (30 mL × 2). The combined organic layers were washed with brine (30 mL × 2), dried over $MgSO_4$, filtered and concentrated under reduced pressure to give a residue. The crude product was purified by reversed-phase HPLC (column: Boston Prime C18 150×30 mm×5 um; mobile phase: [water (ammonia hydroxide v/v)-ACN]; gradient:15% - 35% B over 10 min). Compound **Inter3** (25.9 mg, 121 μmol, 18.4% yield, 100% purity) was obtained as a brown solid. ^{1}H NMR (400 MHz, DMSO-d_6), δ ppm: 7.01 (d, J = 8.6 Hz, 1H), 6.82 - 6.74 (m, 2H), 6.08 - 6.01 (m, 1H), 4.93 (br s, 2H), 2.73 - 2.62 (m, 2H), 2.45 - 2.38 (m, 2H), 1.80 - 1.65 (m, 4H). LC-MS (ESI) *m/z* 215.2 $[M+H]^+$.

4-fluoro-3-methyl-N-(6-oxo-5,6,7,8,9,10-hexahydrophenanthridin-2-yl)benzenesulfonamide ***(HF1).*** To a mixture of **Inter3** (25.0 mg, 117 μmol, 1.00 *eq*) in Py (1.00 mL) was added 4-fluoro-3-methylbenzenesulfonyl chloride (24.3 mg, 116 μmol, 1.00 *eq*). The mixture was stirred at 25 °C for 2 h. The reaction mixture was added 1N HCl (30 mL) and extracted with EtOAc (30 mL). The combined organic layer was washed with 1N HCl (30 mL × 3), dried over

Na_2SO_4, filtered and concentrated under reduced pressure to give a residue. The residue was purified by prep-HPLC (column: Boston Prime C18 150×30 mm×5 um; mobile phase: [water (ammonia hydroxide v/v)-ACN]; gradient: 30%-50% B over 10 min). Compound **HF1** (9.78 mg, 24.8 μmol, 21.3% yield, 98.2% purity) was obtained as a white solid.^{1}H NMR (400 MHz, DMSO-*d6*), δ ppm: 11.57 (br s, 1 H), 10.31 - 9.65 (m, 1 H), 7.69 (br d, *J* = 5.7 Hz, 1 H), 7.55 (br d, *J* = 2.7 Hz, 1 H), 7.34 - 7.26 (m, 2 H), 7.18 - 7.09 (m, 2 H), 2.63 (br s, 2 H), 2.42 (br s, 2 H), 2.24 (s, 3 H), 1.82 - 1.63 (m, 4 H). ^{13}C NMR (101 MHz, DMSO-*d*6), δ(ppm): 164.13, 161.76, 142.27, 134.56, 133.08, 131.60, 130.94, 129.43, 129.06, 127.60, 127.51, 123.77, 120.18, 116.37, 116.04, 25.22, 24.03, 21.86, 21.77, 14.56. LC-MS (ESI) *m/z* 387.1 $[M+H]^+$. HRMS (ESI) calcd for $C_{20}H_{19}N_2O_3FS$ $[M+H]^+$: 387.1173, found 387.1173. Purity: 98.2% (HPLC).

2-((2-(dimethylamino)ethyl)amino)-7,8,9,10-tetrahydrophenanthridin-6(5H)-one ***(HF2).*** To a mixture of 2-aminophenanthridin-6(5H)-one (100 mg, 476 μmol, 1.00 *eq*) in Py (1.00 mL) was added 4-fluoro-3-methylbenzenesulfonyl chloride (99.2 mg, 476 μmol, 1.00 *eq*). The mixture was stirred at 25 °C for 2 h. The reaction mixture was added 1N HCl (10 mL) and extracted with EtOAc (10 mL). The combined organic layer was washed with 1N HCl (10 mL*3), dried over Na_2SO_4, filtered and concentrated under reduced pressure to give a residue. The residue was purified by prep-HPLC (column: Boston Prime C18 150×30 mm×5 um; mobile phase: [water (ammonia hydroxide v/v)-ACN]; gradient: 33%-53% B over 10 min). Compound **HF2** (33.0 mg, 85.4 μmol, 18.0% yield, 99.0% purity) was obtained as a white solid. ^{1}H NMR (400 MHz, DMSO-*d6*), δ ppm: 11.67 (s, 1 H), 10.33 - 10.10 (m, 1 H), 8.31 (dd, *J* = 1.0, 8.0 Hz, 1 H), 8.19 (d, *J* = 8.1 Hz, 1 H), 7.97 (d, *J* = 2.0 Hz, 1 H), 7.92 - 7.85 (m, 1 H), 7.75 (dd, *J* = 2.0,

7.0 Hz, 1 H), 7.70 - 7.58 (m, 2 H), 7.31 (t, J = 9.0 Hz, 1 H), 7.26 - 7.15 (m, 2 H), 2.24 (d, J = 0.9 Hz, 3 H). ^{13}C NMR (101 MHz, DMSO-$d6$), δ(ppm): 164.15, 160.92, 135.81, 134.28, 133.91, 133.52, 132.37, 130.97, 128.81, 128.12, 127.58, 127.52, 126.42, 126.30, 124.19, 122.54, 118.24, 117.45, 116.12, 14.56. LC-MS (ESI) *m/z* 383.1 [M+H]$^+$. HRMS (ESI) calcd for $C_{20}H_{15}N_2O_3FS$ [M+H]$^+$: 383.0860, found 383.0865. Purity: 99.0% (HPLC).

2-(dimethylamino)-N-(6-oxo-5,6-dihydrophenanthridin-2-yl)acetamide ***(PJ34).*** ^{1}H NMR (400 MHz, DMSO-d_6), δ ppm: 11.97 - 11.28 (m, 1H), 9.83 (s, 1H), 8.67 (d, J = 1.0 Hz, 1H), 8.31 (t, J = 8.0 Hz, 2H), 7.93 - 7.76 (m, 2H), 7.65 (t, J = 7.5 Hz, 1H), 7.30 (d, J = 8.8 Hz, 1H), 3.10 (s, 2H), 2.31 (s, 6H). ^{13}C NMR (101 MHz, DMSO-$d6$), δ(ppm): 169.19, 160.99, 134.47, 134.08, 133.33, 133.11, 128.51, 128.09, 126.30, 122.75, 122.54, 117.86, 116.72, 113.95, 63.81, 45.94. LC-MS (ESI) *m/z* 296.0 [M+H]$^+$. Purity: 97.3% (HPLC).

2-(dimethylamino)-N-(6-oxo-5,6,7,8,9,10-hexahydrophenanthridin-2-yl)acetamide ***(HF3)***. To a solution of 2-(dimethylamino)acetic acid (144 mg, 1.40 mmol, 1.50 *eq*) in DCM (2.00 mL) at 25 °C was added **Inter3** (200 mg, 933 μmol, 1.00 *eq*), DCC (385 mg, 1.87 mmol, 378 μL, 2.00 *eq*) and DMAP (11.4 mg, 93.3 μmol, 0.10 *eq*). The mixture was stirred at 25 °C for 2 h. The reaction mixture was added H_2O 2 mL. The reaction mixture was extracted with DCM 9.00 mL (3.00 mL × 3), dried over Na_2SO_4. Filtered and concentrated under reduced pressure to give a residue. The residue was purified by prep-HPLC (column: C18 150×30mm; mobile phase: [Water (HCl)-MeCN]; gradient: 0%-36% B over 9 min). **Compound HF3** (5.00 mg, 16.7 μmol, 1.79 % yield) was obtained as a white solid. ^{1}H NMR (400 MHz, DMSO-d6), δ ppm: 11.43 - 11.85 (m, 1 H), 10.83 - 11.15 (m, 1 H), 9.65 - 10.30 (m, 1 H), 7.88 - 8.19 (m, 1 H), 7.54 - 7.76 (m, 1 H), 7.16 - 7.33 (m, 1 H), 4.01 - 4.32 (m, 2 H), 2.80 - 2.98 (m, 6 H), 2.70

- 2.79 (m, 2 H), 2.40 - 2.49 (m, 2 H), 1.76 - 1.88 (m, 2 H), 1.64 - 1.76 (m, 2 H). ^{13}C NMR (101 MHz, DMSO-*d*6), δ(ppm): 161.90, 160.62, 133.97, 132.45, 129.29, 122.03, 120.04, 116.10, 114.01, 111.04, 49.06, 44.01, 25.34, 24.07, 21.91, 21.81. LC-MS (ESI) *m/z* 300.1 $[M+H]^+$. HRMS (ESI) calcd for $C_{17}H_{21}N_3O_2$ $[M+H]^+$: 300.1707, found 300.1681. Purity: 96.6% (HPLC).

N-(2-formylphenyl)butyramide ***(Inter4).*** To a solution of 2-aminobenzaldehyde (6.00 g, 49.5 mmol, 1.00 *eq*) in THF (50.0 mL) was added Py (3.92 g, 49.5 mmol, 4.00 mL, 1.00 *eq*) and butanoyl chloride (5.28 g, 49.5 mmol, 5.18 mL, 1.00 *eq*) at 0 °C and purged with N_2 for 3 times. The mixture was stirred at 25 °C for 2 h under N_2 atmosphere. The residue was diluted with 1M HCl 50.0 mL. The reaction mixture was concentrated under reduced pressure to remove THF and extracted with EtOAc 300 mL (100 mL × 3). The combined organic layers were washed with brine 200 mL (100 mL × 2), dried over Na_2SO_4, filtered and concentrated under reduced pressure to give a residue. The residue was purified by flash silica gel chromatography (SiO_2, Eluent of 0~50% Ethyl acetate/Petroleum ether gradient). **Inter4** (8.60 g, 45.0 mmol, 90.8% yield) was obtained as a yellow oil. ^{1}H NMR (400 MHz, DMSO-d6), δ ppm: 10.69 - 10.89 (m, 1 H), 9.88 - 10.07 (m, 1 H), 8.11 - 8.29 (m, 1 H), 7.79 - 7.95 (m, 1 H), 7.57 - 7.77 (m, 1 H), 7.24 - 7.43 (m, 1 H), 2.34 - 2.45 (m, 2 H), 1.58 - 1.73 (m, 2 H), 0.91 - 1.00 (m, 3 H). LC-MS (ESI) *m/z* 192.2 $[M+H]^+$.

3-ethylquinolin-2(1H)-one ***(Inter5).*** To a solution of **Inter4** (7.00 g, 36.6 mmol, 1.00 *eq*) in DMF (70.0 mL) was added Cs_2CO_3 (59.6 g, 183 mmol, 5.00 *eq*). The mixture was stirred at 80 °C for 16 h under N_2 atmosphere. The reaction mixture was filtered. The reaction mixture was added H_2O 200 mL. The reaction mixture was extracted with Ethyl acetate 600 mL (200 mL × 3). The combined organic layers were washed with brine 200 mL, dried over Na_2SO_4,

filtered and concentrated under reduced pressure to give a residue. The residue was purified by flash silica gel chromatography (SiO_2, Eluent of 0~50% Ethyl acetate/Petroleum ether gradient). **Inter5** (2.40 g, 13.9 mmol, 37.9% yield) was obtained as a yellow oil. ^{1}H NMR (400 MHz, DMSO-d6), δ ppm: 11.44 - 11.92 (m, 1 H), 7.69 - 7.75 (m, 1 H), 7.57 - 7.65 (m, 1 H), 7.39 - 7.48 (m, 1 H), 7.24 - 7.33 (m, 1 H), 7.10 - 7.21 (m, 1 H), 2.51 - 2.52 (m, 2 H), 1.14 - 1.25 (m, 3 H). LC-MS (ESI) *m/z* 173.8 $[M+H]^+$.

3-ethyl-6-nitroquinolin-2(1H)-one ***(Inter6).*** To an ice-cold solution of concentrated H_2SO_4 (2.00 mL) and concentrated HNO_3 (1.38 g, 21.9 mmol, 986 μL, 15.2 *eq*). **Inter5** (250 mg, 1.44 mmol, 1 *eq*) was added in small portions. And then the mixture was stirred at 0 °C for 2 h. The mixture was poured into ice. The solid was formed and filtered, washed with water and dried. The residue was purified by prep-HPLC (column: C18 150×30 mm; mobile phase: [Water (HCl)-MeCN]; gradient: 22% - 62% B over 9 min). **Inter6** (10.7 mg, 49.0 μmol, 3.40% yield) was obtained as a yellow solid. ^{1}H NMR (400 MHz, DMSO-d6), δ ppm: 12.26 - 12.48 (m, 1 H), 8.64 - 8.76 (m, 1 H), 8.27 - 8.45 (m, 1 H), 8.00 - 8.10 (m, 1 H), 7.41 - 7.56 (m, 1 H), 2.59 - 2.64 (m, 2 H), 1.23 - 1.29 (m, 3 H). LC-MS (ESI) *m/z* 219.2 $[M+H]^+$.

6-amino-3-ethylquinolin-2(1H)-one ***(Inter7).*** To a solution of **Inter6** (100 mg, 458 μmol, 1.00 *eq*) in H_2O (1 mL) and EtOH (1 mL) was added Fe (51.2 mg, 916 μmol, 2.00 *eq*) and NH_4Cl (49.0 mg, 916 μmol, 2.00 *eq*). The mixture was stirred at 80 °C for 4 h. The reaction mixture was added H_2O 10.0 mL. The reaction mixture was extracted with Ethyl acetate 60.0 mL (20.0 mL × 3), dried over Na_2SO_4, filtered and concentrated under reduced pressure to give a residue. The residue was purified by prep-HPLC (column: Boston Prime C18 150×30 mm×5 um; mobile phase: [water (ammonia hydroxide v/v)-ACN]; gradient: 10% - 30% B over 10 min).

Inter7 (9.80 mg, 52.1 μmol, 11.4% yield) was obtained as a white solid. ^{1}H NMR (400 MHz, DMSO-d6), δ ppm: 11.30 - 11.38 (m, 1 H), 7.39 - 7.55 (m, 1 H), 6.97 - 7.05 (m, 1 H), 6.74 - 6.81 (m, 1 H), 6.66 - 6.72 (m, 1 H), 4.76 - 5.13 (m, 2 H), 2.42 - 2.48 (m, 2 H), 1.09 - 1.18 (m, 3 H). LC-MS (ESI) *m/z* 189.3 $[M+H]^+$.

N-(3-ethyl-2-oxo-1,2-dihydroquinolin-6-yl)-4-fluoro-3-methylbenzenesulfonamide ***(HF4).*** To a solution of **Inter7** (30.0 mg, 159 μmol, 1.00 eq) in Py (0.50 mL) at 0 °C was added 4-fluoro-3-methyl-benzenesulfonyl chloride (33.3 mg, 159 μmol, 1.00 *eq*). The mixture was stirred at 25 °C for 3 h. The reaction mixture was added H_2O 5.00 mL. The reaction mixture was extracted with EtOAc 15.0 mL (5 mL × 3), dried over Na_2SO_4. Filtered and concentrated under reduced pressure to give a residue. The residue was purified by prep-HPLC (column: C18 150×30 mm; mobile phase: [Water (HCl)-MeCN]; gradient: 20% - 60% B over 9 min). **HF4** (17.9 mg, 49.6 μmol, 31.1 % yield) was obtained as a white solid. ^{1}H NMR (400 MHz, DMSO-d6), δ ppm 11.56 - 11.84 (m, 1 H), 10.01 - 10.27 (m, 1 H), 7.67 - 7.73 (m, 1 H), 7.63 - 7.66 (m, 1 H), 7.52 - 7.59 (m, 1 H), 7.31 - 7.33 (m, 1 H), 7.26 - 7.30 (m, 1 H), 7.14 - 7.18 (m, 1 H), 7.10 - 7.14 (m, 1 H), 2.49 (br s, 2 H), 2.21 - 2.27 (m, 3 H), 1.05 - 1.18 (m, 3 H). ^{13}C NMR (101 MHz, DMSO-*d*6), δ(ppm): 162.17, 161.71, 136.59, 135.46, 134.83, 131.63, 130.84, 127.48, 127.43, 124.10, 120.16, 119.88, 116.46, 116.30, 115.97, 23.35, 14.58, 13.10. LC-MS (ESI) *m/z* 361.0 $[M+H]^+$. HRMS (ESI) calcd for $C_{18}H_{17}N_2O_3FS$ $[M+H]^+$: 361.1017, found 361.1020. Purity: 99.2% (HPLC).

2-(dimethylamino)-N-(3-ethyl-2-oxo-1,2-dihydroquinolin-6-yl)acetamide ***(HF5).*** To a solution of 2-(dimethylamino) acetic acid (59.2 mg, 574 μmol, 1.20 *eq*) in DMF (0.50 mL) was added **Inter7** (90.0 mg, 478 μmol, 1.00*eq*), DIEA (124 mg, 956 μmol, 167 μL, 2.00 *eq*) and HATU

(273 mg, 717 μmol, 1.50 *eq*), the mixture was stirred at 25 °C for 2 h. The reaction mixture was added H_2O 2.00 mL. The reaction mixture was extracted with EtOAc 10 mL, dried over Na_2SO_4. Filtered and concentrated under reduced pressure to give a residue. The residue was purified by prep-HPLC (column: C18 150×30 mm; obile phase: [Water NH_3H_2O-NH_4HCO_3)-MeCN]; gradient: 1%-41% B over 9 min). **Compound HF5** (28.6 g, 105 μmol, 21.9% yield) as obtained as a white solid. ^{1}H NMR (400 MHz, DLC-MSO-d6), δ ppm: 11.59 - 11.76 (m, 1 H), 9.66 - 9.83 (m, 1 H), 7.91 - 8.05 (m, 1 H), 7.63 - 7.70 (m, 1 H), 7.55 - 7.63 (m, 1 H), 7.12 - 7.29 (m, 1 H), 3.00 - 3.14 (m, 2 H), 2.49 - 2.54 (m, 2 H), 2.16 - 2.35 (m, 6 H), 1.08 - 1.25 (m, 3 H). ^{13}C NMR (101 MHz, DMSO-*d*6), δ(ppm): 168.96, 162.19, 136.19, 135.12, 134.46, 133.25, 122.52, 119.81, 117.58, 115.29, 63.71, 45.85, 23.40, 13.23. LC-MS (ESI) *m/z* 274.1 $[M+H]^+$. HRMS (ESI) calcd for $C_{15}H_{19}N_3O_2$ $[M+H]^+$: 274.1550, found 274.1537. Purity: 99.4% (HPLC).

^{1}H NMR, ^{13}C NMR, LC-MS, HRMS, and HPLC spectra for designed compounds

4-nitro-2-(4,4,5,5-tetramethyl-1,3,2-dioxaborolan-2-yl)aniline (Inter1)

^{1}H NMR

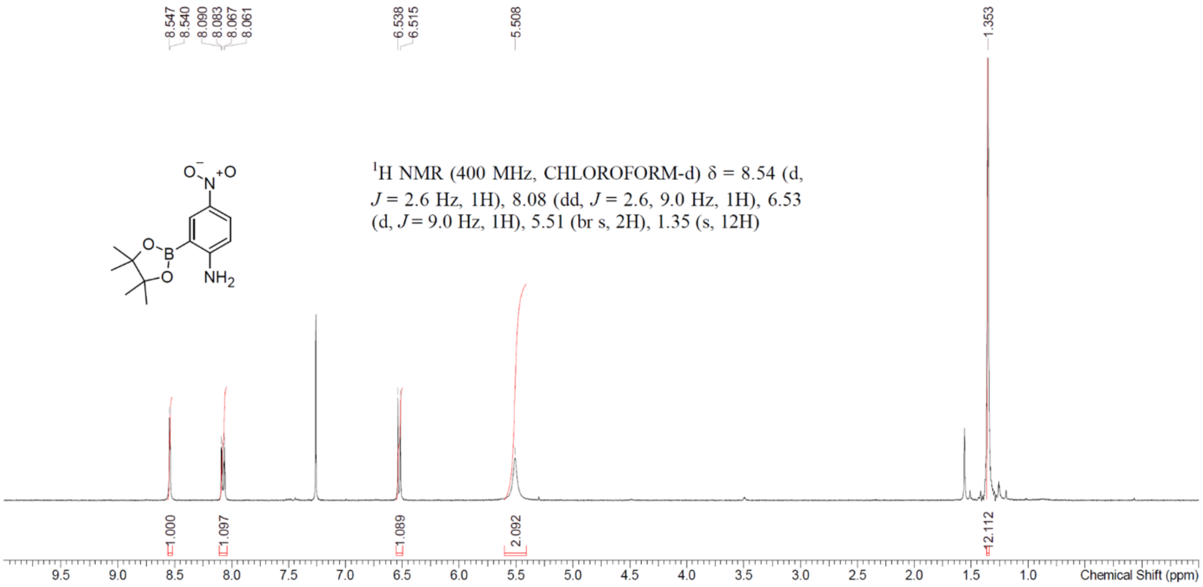

LC-MS

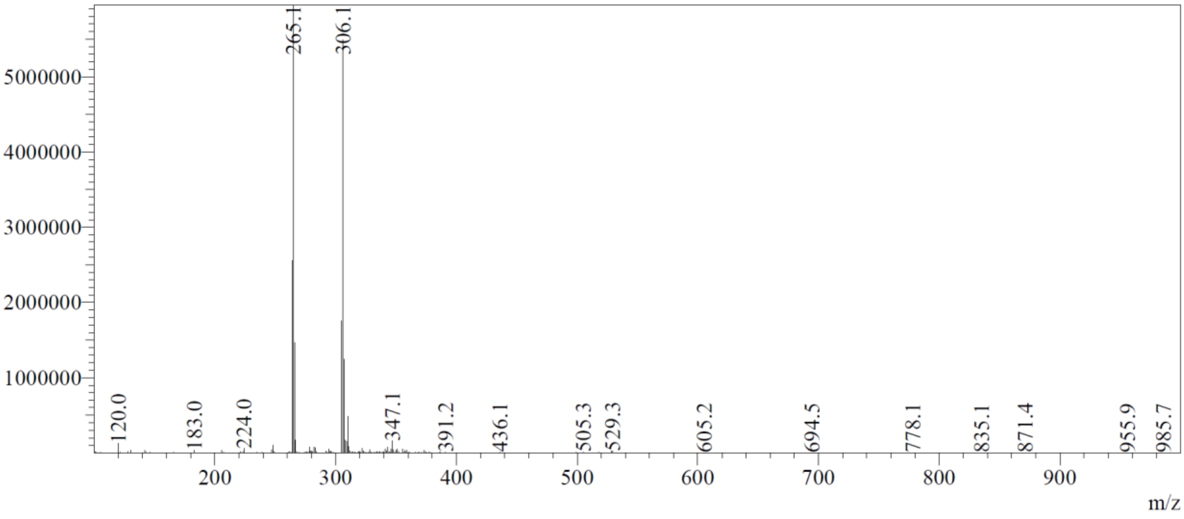

2-nitro-7,8,9,10-tetrahydrophenanthridin-6(5H)-one (Inter2)

^{1}H NMR

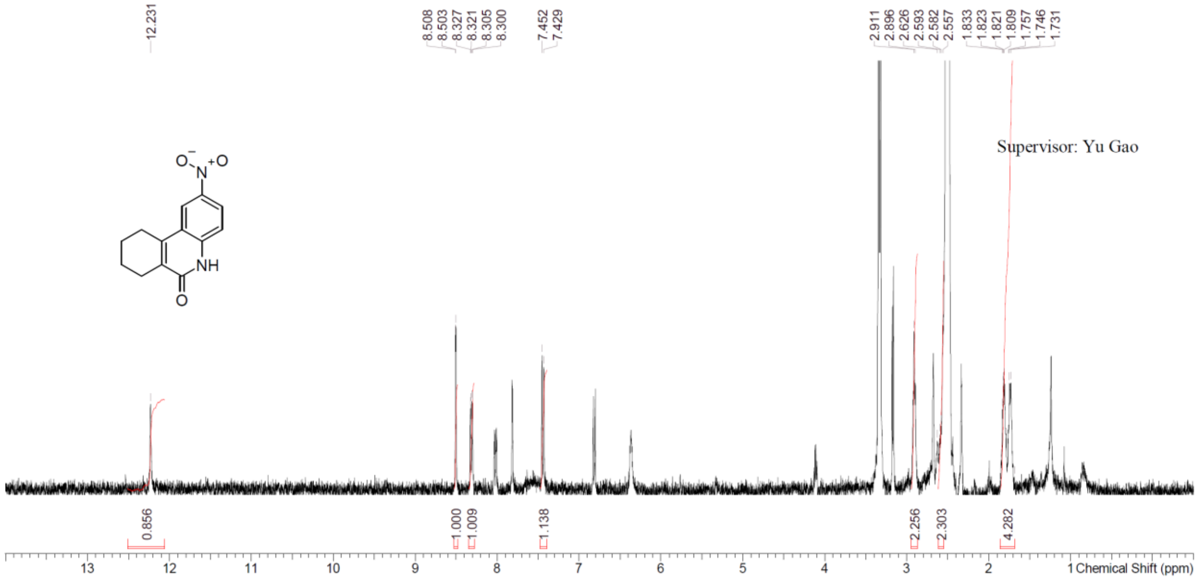

LC-MS

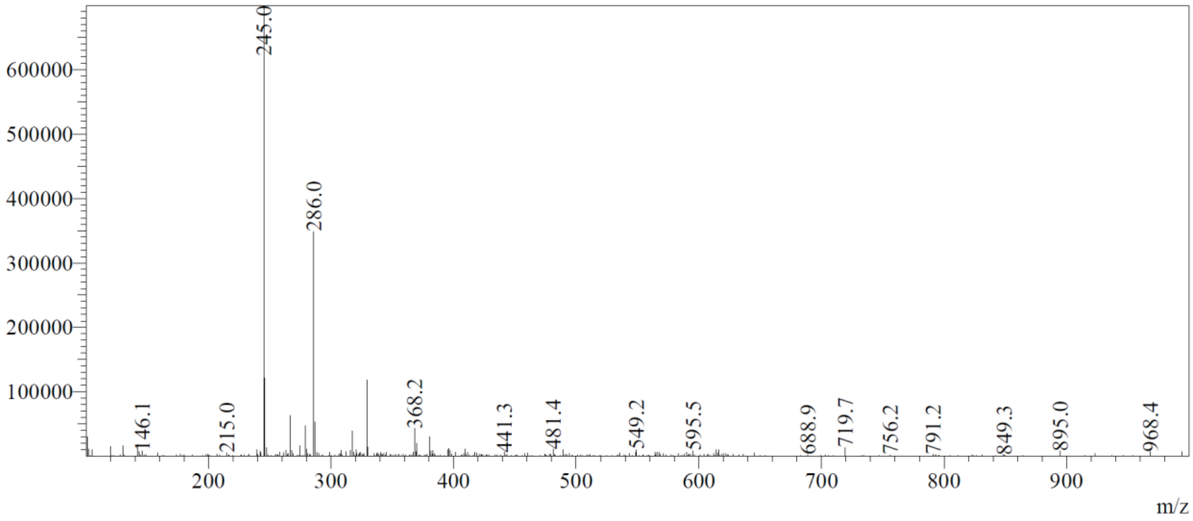

2-amino-7,8,9,10-tetrahydrophenanthridin-6(5H)-one (Inter3)

^{1}H NMR

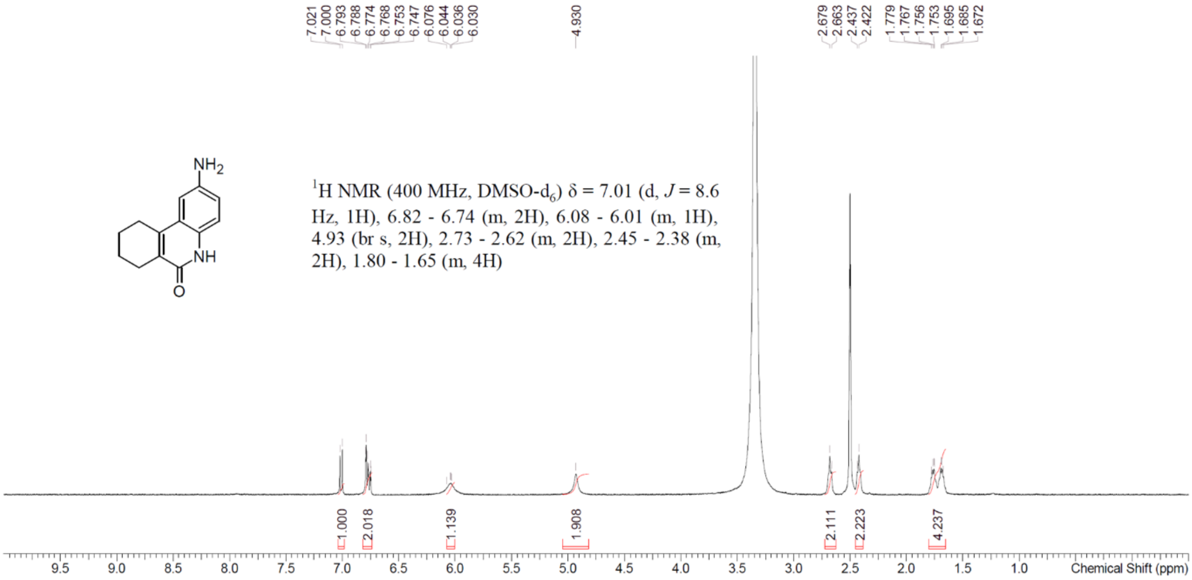

LC-MS

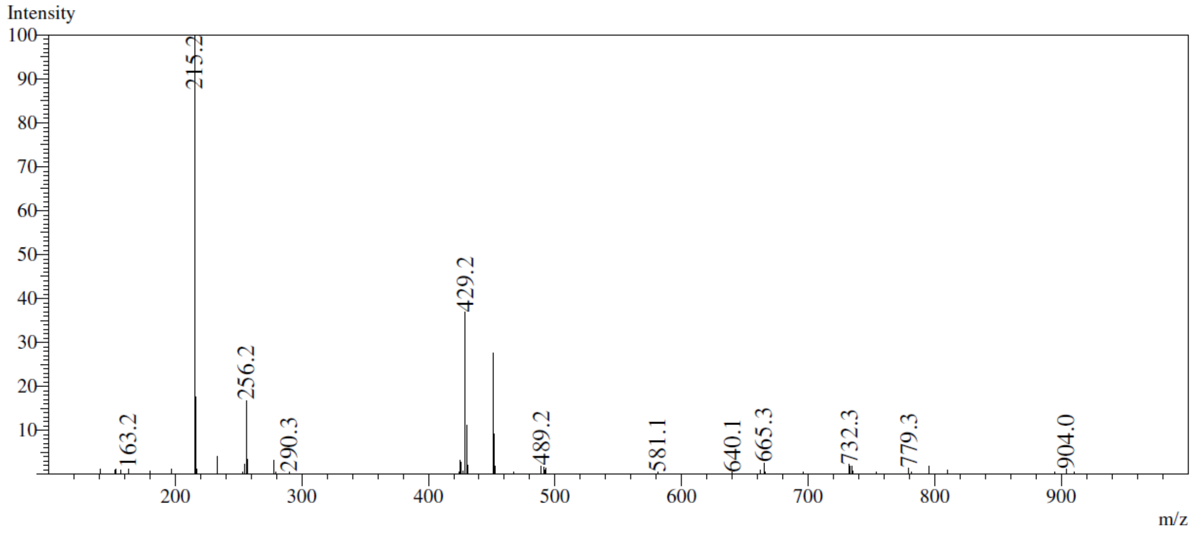

4-fluoro-3-methyl-N-(6-oxo-5,6,7,8,9,10-hexahydrophenanthridin-2-yl)benzenesulfonamide (HF1)

^{1}H NMR

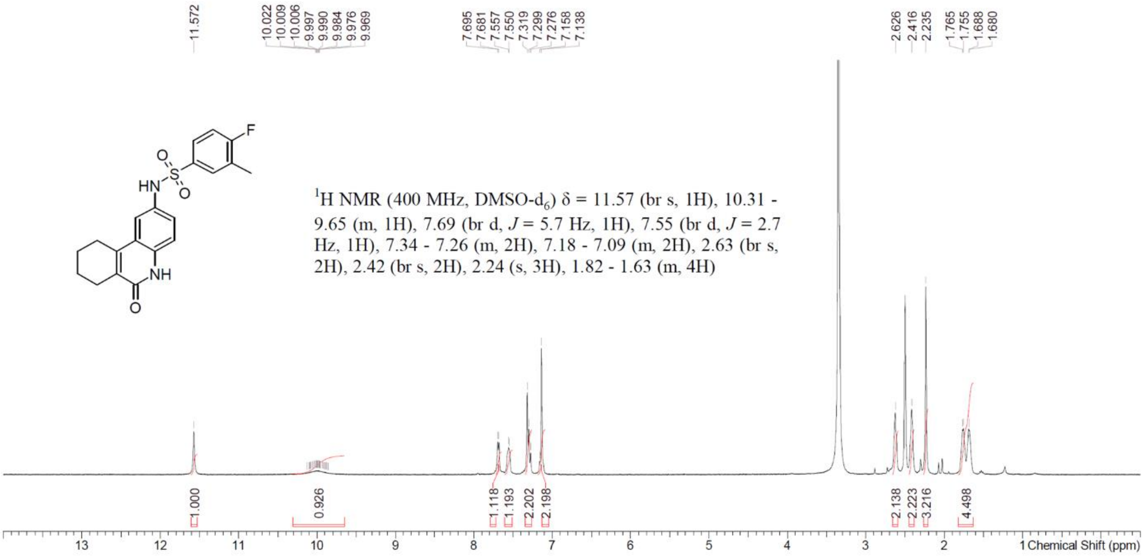

^{13}C NMR

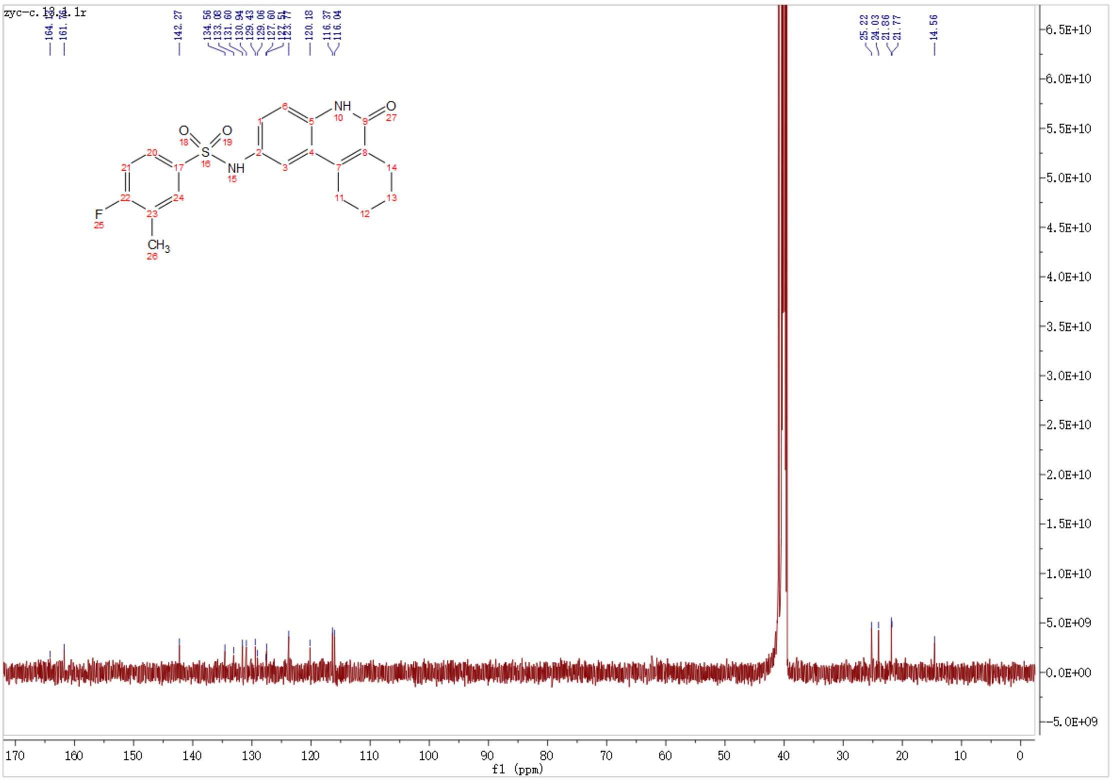

LC-MS

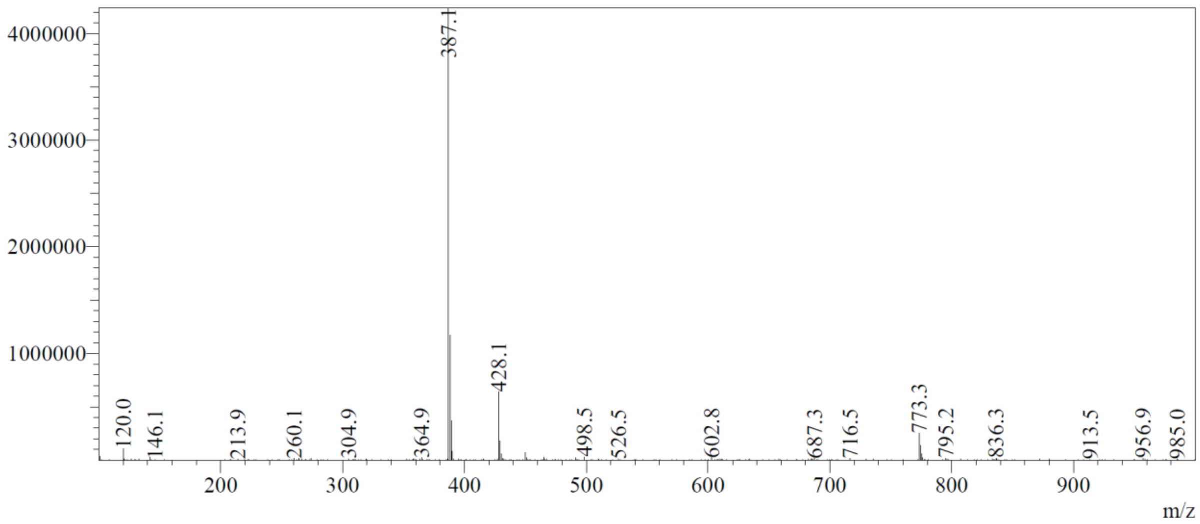

HPLC

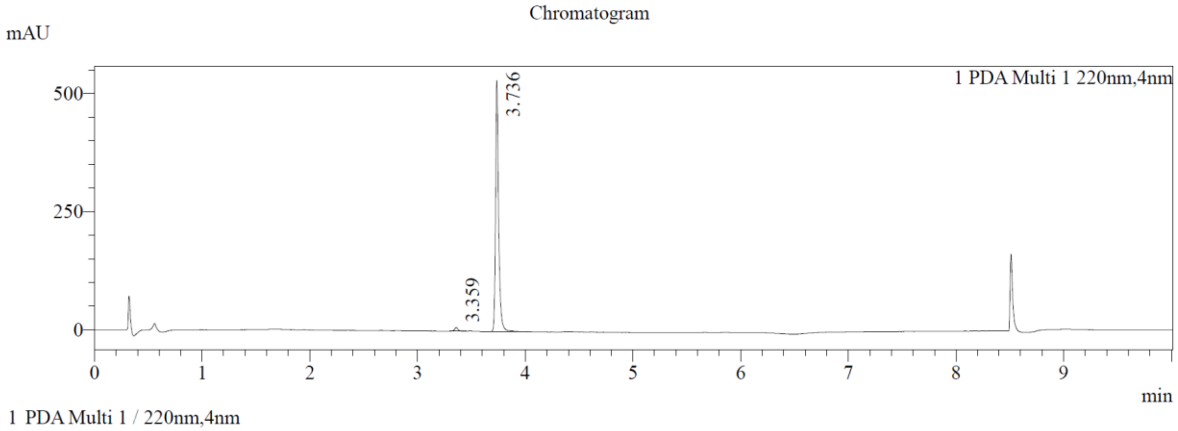

Integration Result

Peak Table

PDA Ch1 220nm

Peak#	Ret. Time	Height	Height%	USP Width	Area	Area%
1	3.359	7922	1.580	0.060	18954	1.769
2	3.736	493335	98.420	0.050	1052653	98.231

HRMS

Elmt	Val.	Min	Max	Elmt	Val.	Min	Max	Elmt	Val.	Min	Max	Elmt	Val.	Min	Max	Use Adduct
H	1	19	19	O	2	2	4	P	3	0	0	I	3	0	0	H
2H	1	0	0	18O	2	0	0	S	2	0	1					Na
C	4	20	20	F	1	0	1	Cl	1	0	0					
N	3	2	2	Si	4	0	0	Br	1	0	0					

Error Margin (ppm):	5000	DBE Range:	not fixed	Electron Ions:	both
HC Ratio:	unlimited	Apply N Rule:	yes	Use MSn Info:	yes
Max Isotopes:	all	Isotope RI (%):	1.00	Isotope Res:	10000
MSn Iso RI (%):	75.00	MSn Logic Mode:	AND	Max Results:	23

Event#: 1 MS(E+) Ret. Time : 1.013 Scan# : 153

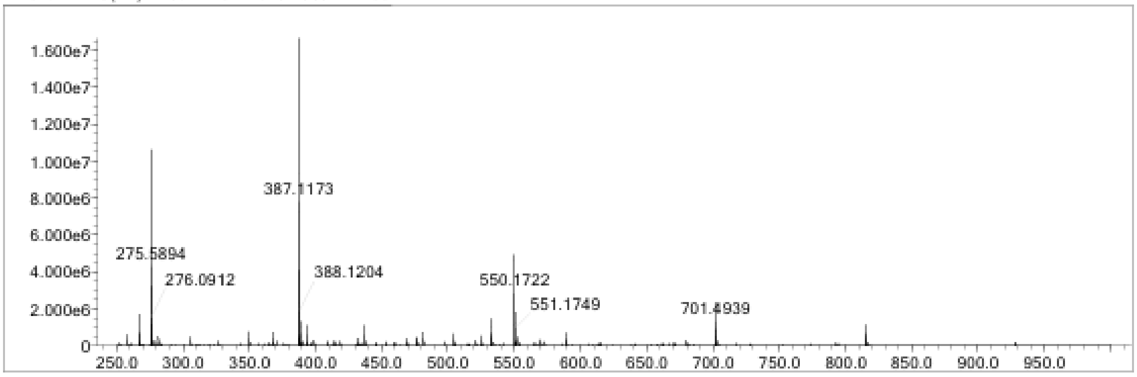

Measured region for 387.1173 m/z

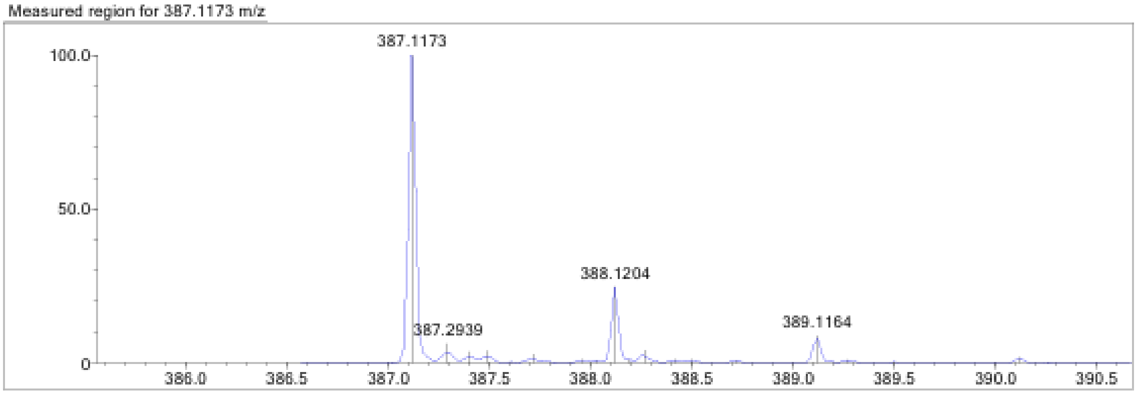

C20 H19 N2 O3 F S [M+H]+ : Predicted region for 387.1173 m/z

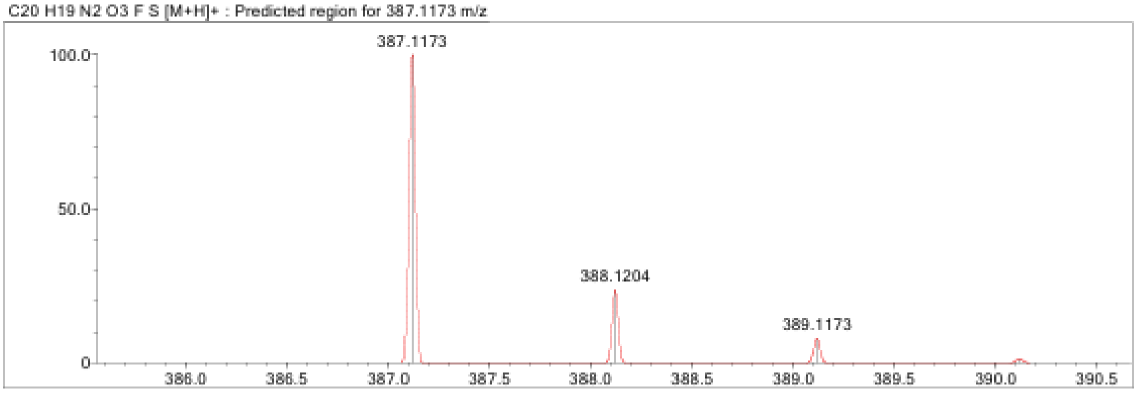

Rank	Score	Formula (M)	Ion	Meas. m/z	Pred. m/z	Df. (mDa)	Df. (ppm)	Iso	DBE
1	96.38	C20 H19 N2 O3 F S	[M+H]+	387.1173	387.1173	-0.0	0.00	96.38	12.0

2-((2-(dimethylamino)ethyl)amino)-7,8,9,10-tetrahydrophenanthridin-6(5H)-one (HF2)

^{1}H NMR

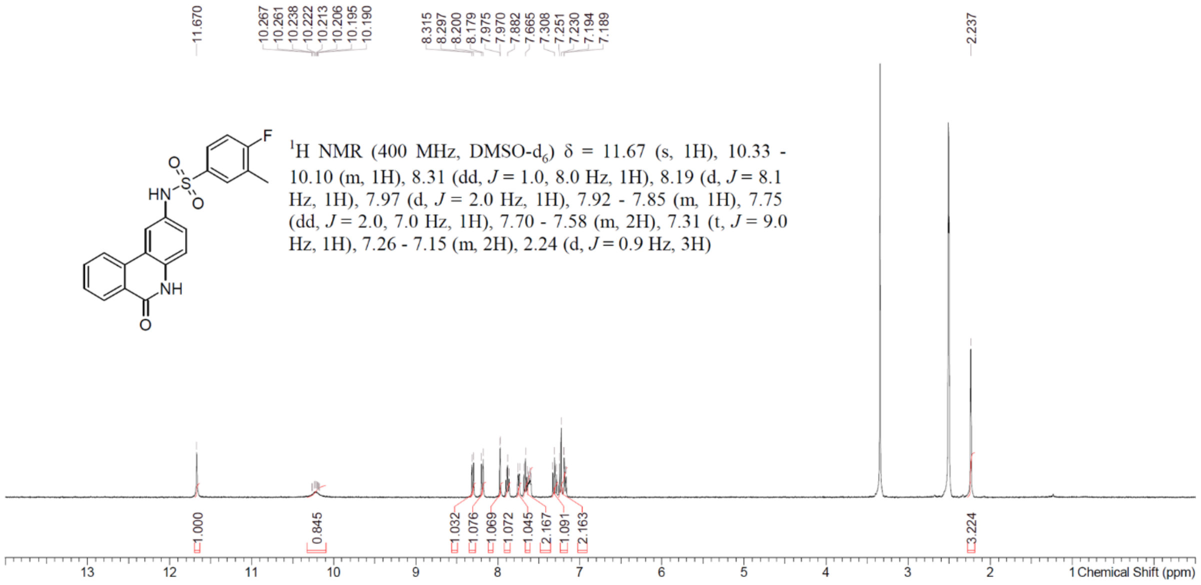

^{13}C NMR

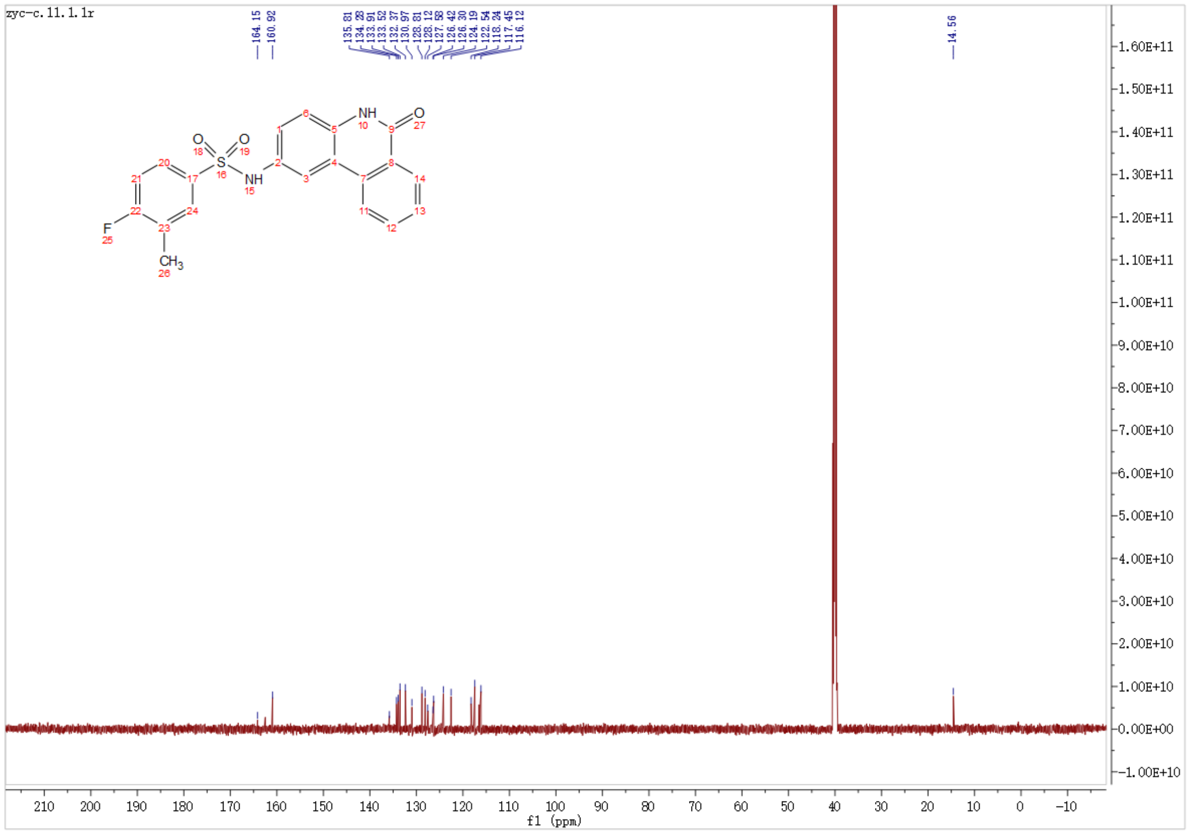

LC-MS

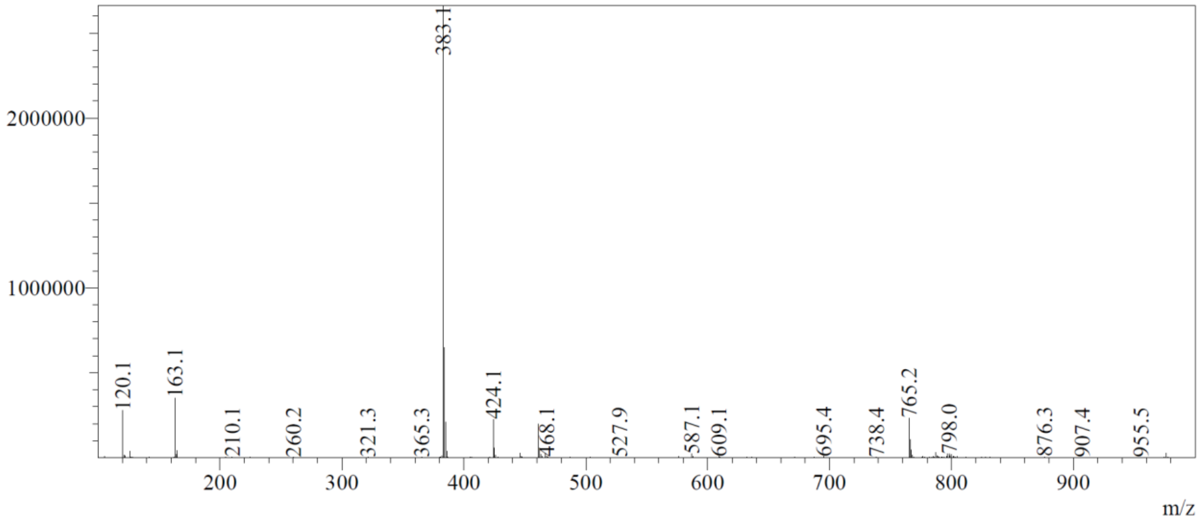

HPLC

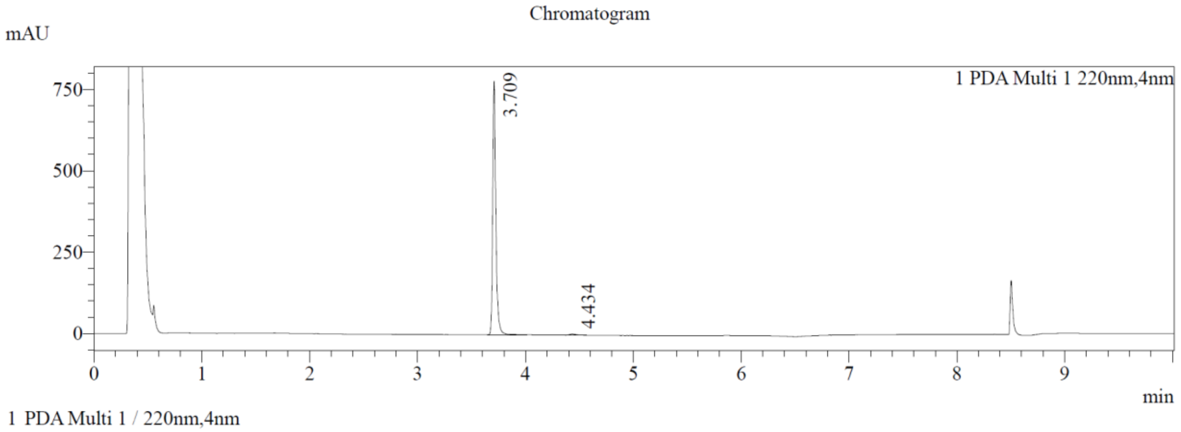

1 PDA Multi 1 / 220nm,4nm

Integration Result

Peak Table

PDA Ch1 220nm

Peak#	Ret. Time	Height	Height%	USP Width	Area	Area%
1	3.709	762221	99.501	0.050	1556778	99.010
2	4.434	3822	0.499	0.099	15559	0.990

HRMS

Elmt	Val.	Min	Max	Elmt	Val.	Min	Max	Elmt	Val.	Min	Max	Elmt	Val.	Min	Max	Use Adduct
H	1	15	15	O	2	2	4	P	3	0	0	I	3	0	0	H
2H	1	0	0	18O	2	0	0	S	2	0	1					Na
C	4	20	20	F	1	0	1	Cl	1	0	0					
N	3	2	2	Si	4	0	0	Br	1	0	0					

Error Margin (ppm): 5000
HC Ratio: unlimited
Max Isotopes: all
MSn Iso RI (%): 75.00

DBE Range: not fixed
Apply N Rule: yes
Isotope RI (%): 1.00
MSn Logic Mode: AND

Electron Ions: both
Use MSn Info: yes
Isotope Res: 10000
Max Results: 23

Event#: 1 MS(E+) Ret. Time : 0.933 Scan# : 141

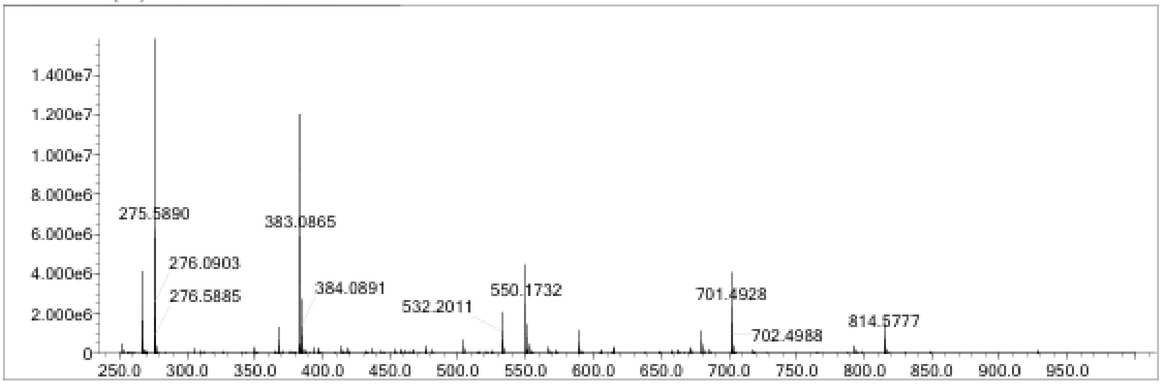

Measured region for 383.0865 m/z

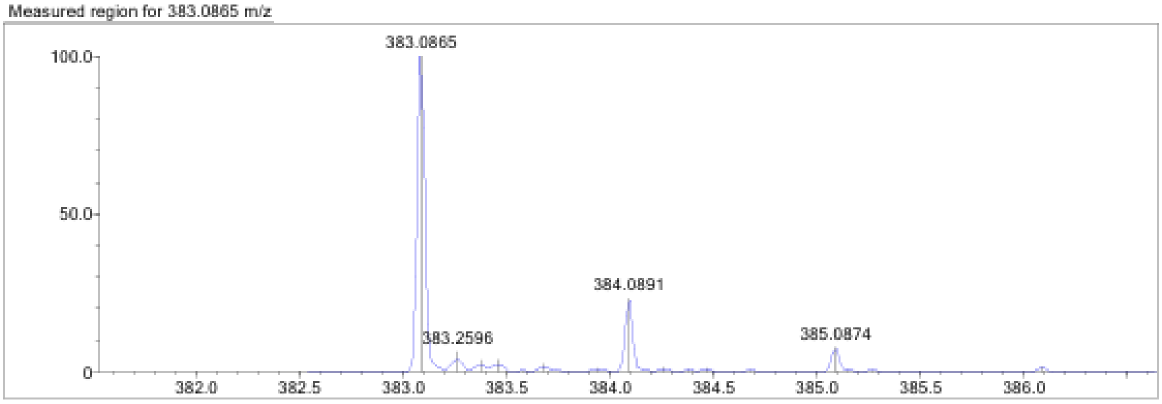

C20 H15 N2 O3 F S [M+H]+ : Predicted region for 383.0860 m/z

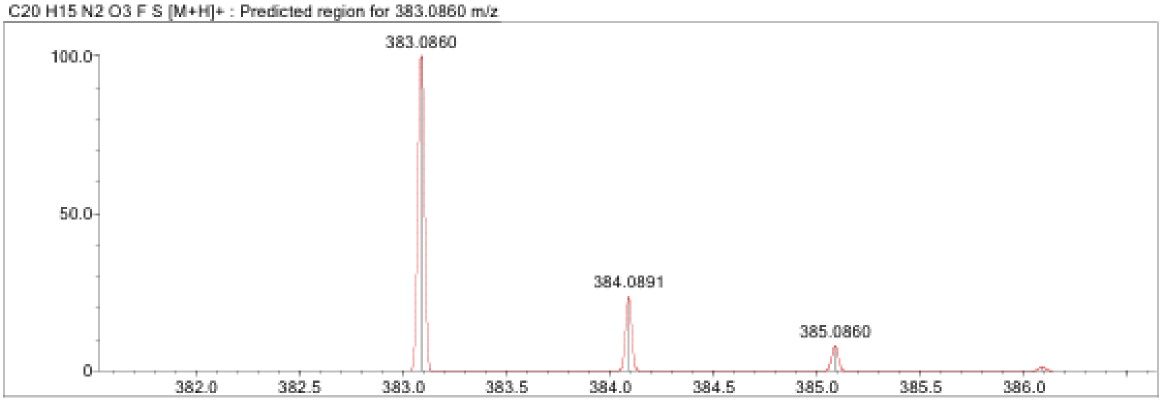

Rank	Score	Formula (M)	Ion	Meas. m/z	Pred. m/z	Df. (mDa)	Df. (ppm)	Iso	DBE
1	81.29	C20 H15 N2 O3 F S	[M+H]+	383.0865	383.0860	0.5	1.31	81.93	14.0

2-(dimethylamino)-N-(6-oxo-5,6-dihydrophenanthridin-2-yl)acetamide (PJ34)

^{1}H NMR

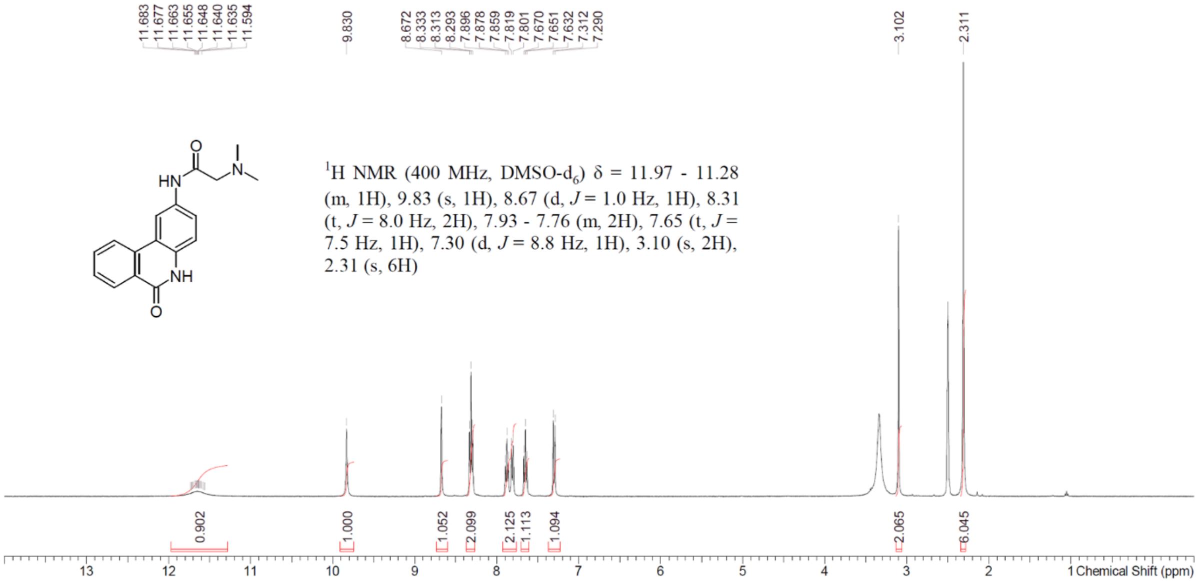

^{13}C NMR

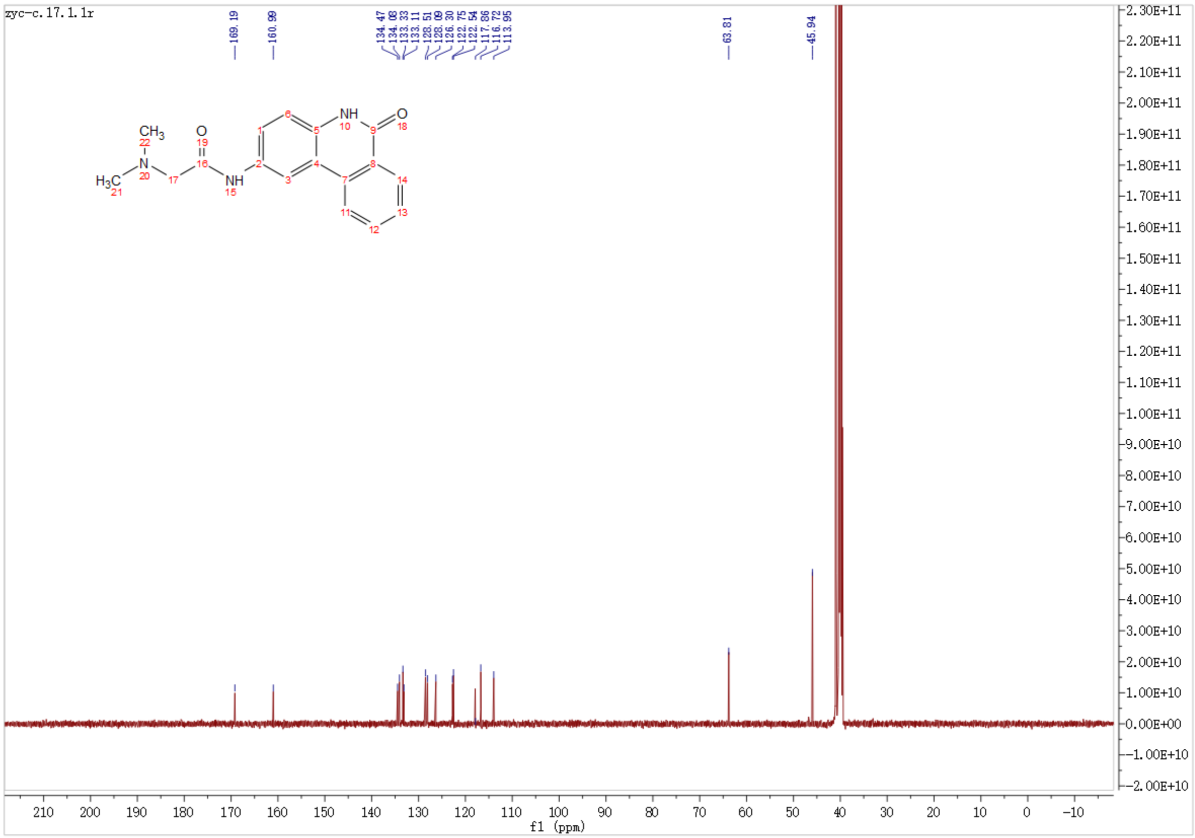

LC-MS

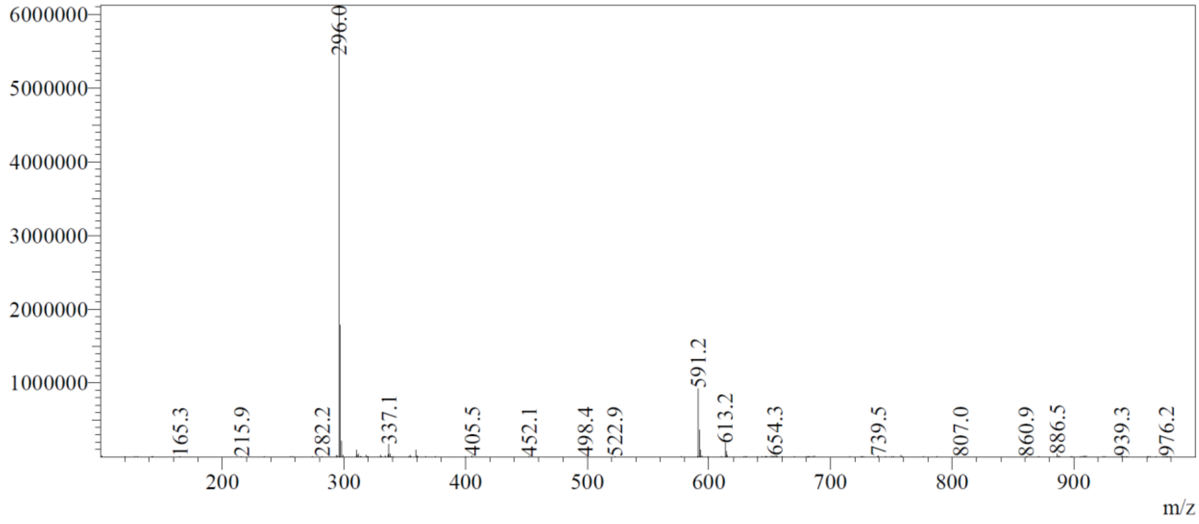

HPLC

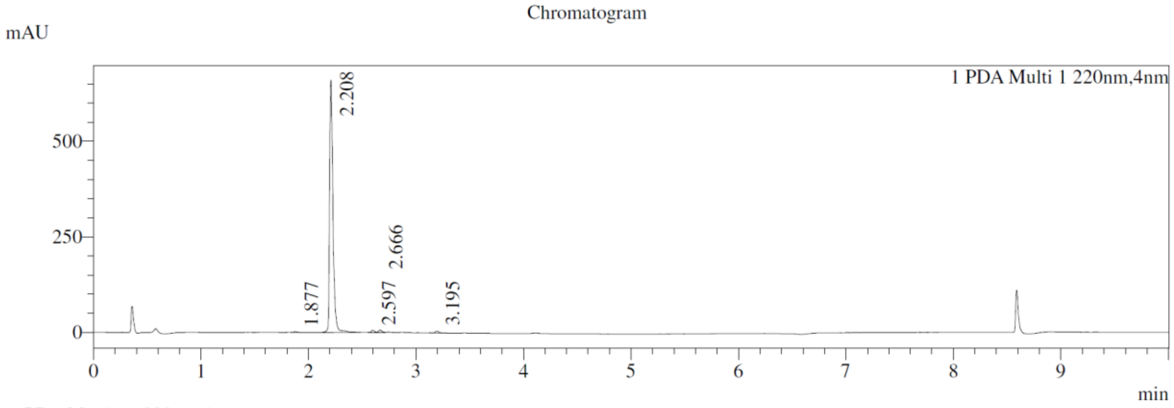

1 PDA Multi 1 / 220nm,4nm

===

Integration Result

===

Peak Table

PDA Ch1 220nm

Peak#	Ret. Time	Height	Height%	USP Width	Area	Area%
1	1.877	2442	0.379	0.057	5226	0.354
2	2.208	626294	97.136	0.055	1434508	97.299
3	2.597	5350	0.830	0.051	10303	0.699
4	2.666	5679	0.881	0.057	12049	0.817
5	3.195	4996	0.775	0.060	12239	0.830

2-(dimethylamino)-N-(6-oxo-5,6,7,8,9,10-hexahydrophenanthridin-2-yl)acetamide (HF3)

^{1}H NMR

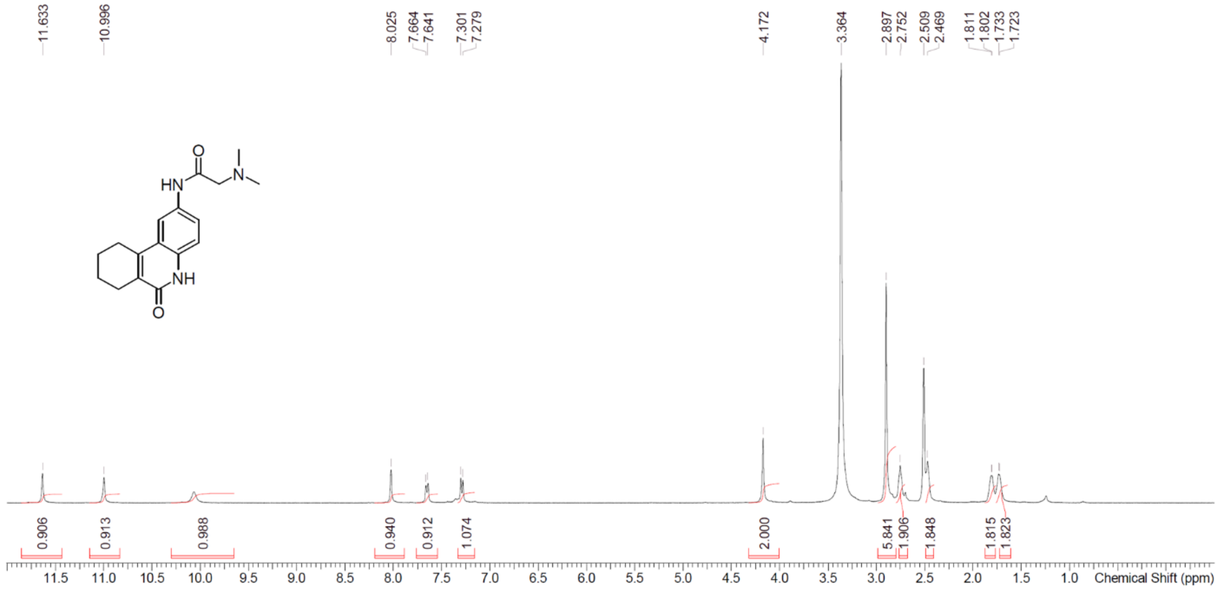

^{13}C NMR

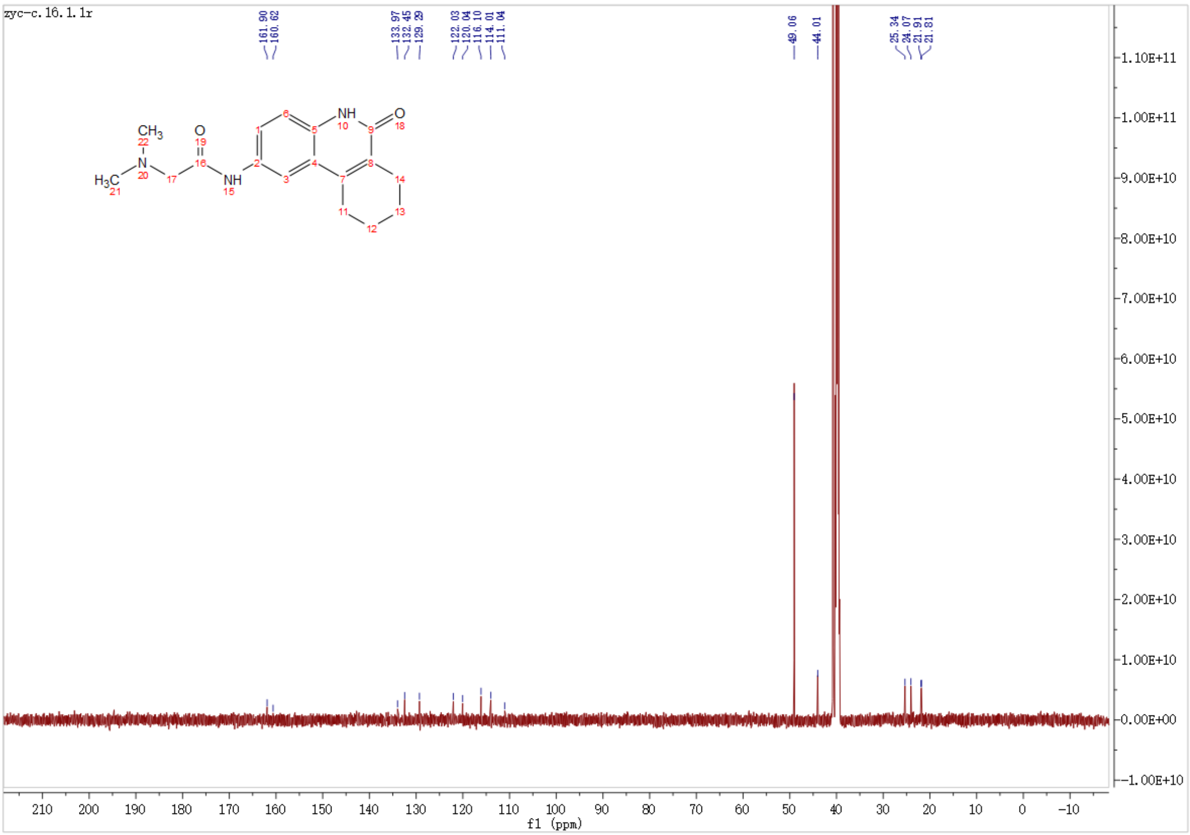

LC-MS

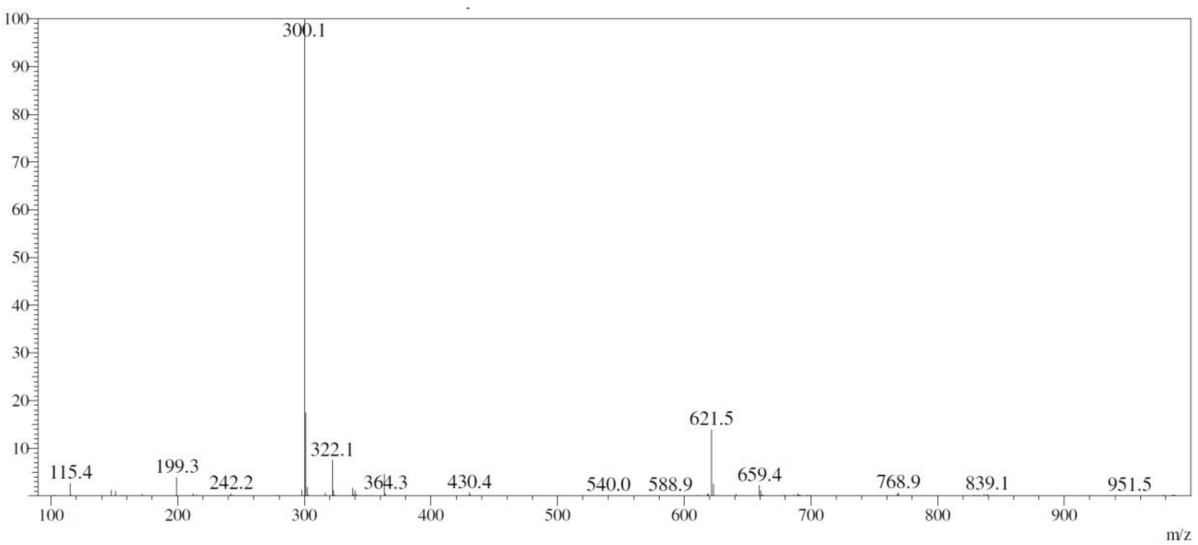

HPLC

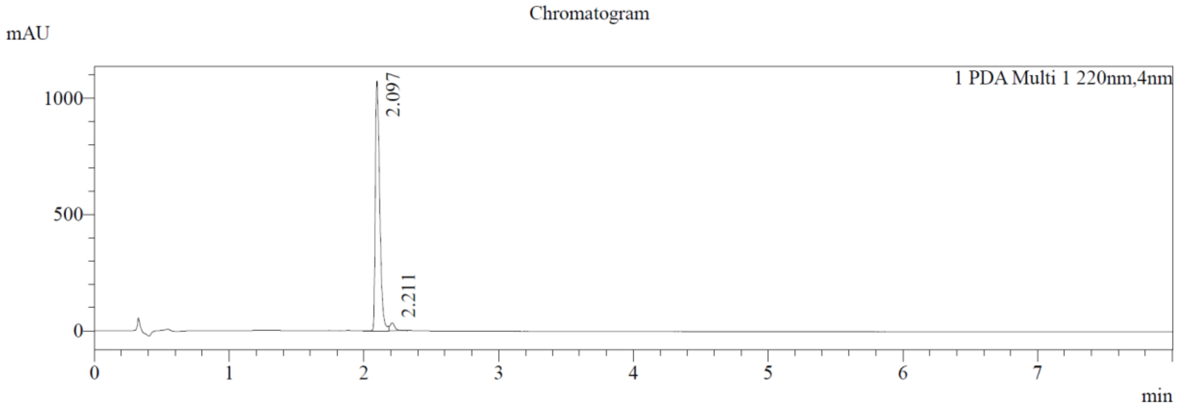

1 PDA Multi 1 / 220nm,4nm

Integration Result

Peak Table

PDA Ch1 220nm

Peak#	Ret. Time	Height	Height%	USP Width	Area	Area%
1	2.097	1029498	96.822	0.060	2458438	96.595
2	2.211	33795	3.178	0.073	86650	3.405

HRMS

Elmt	Val.	Min	Max	Elmt	Val.	Min	Max	Elmt	Val.	Min	Max	Elmt	Val.	Min	Max	Use Adduct
H	1	21	21	N	3	3	3	Si	4	0	0	Br	1	0	0	H
2H	1	0	0	O	2	2	2	P	3	0	0	I	3	0	1	Na
B	3	0	0	18O	2	0	0	S	2	0	2					
C	4	17	17	F	1	0	0	Cl	1	0	0					

Error Margin (ppm):	50	DBE Range:	not fixed	Electron Ions:	both
HC Ratio:	unlimited	Apply N Rule:	yes	Use MSn Info:	yes
Max Isotopes:	all	Isotope RI (%):	1.00	Isotope Res:	10000
MSn Iso RI (%):	75.00	MSn Logic Mode:	AND	Max Results:	23

Event#: 1 MS(E+) Ret. Time : 1.133 Scan# : 171

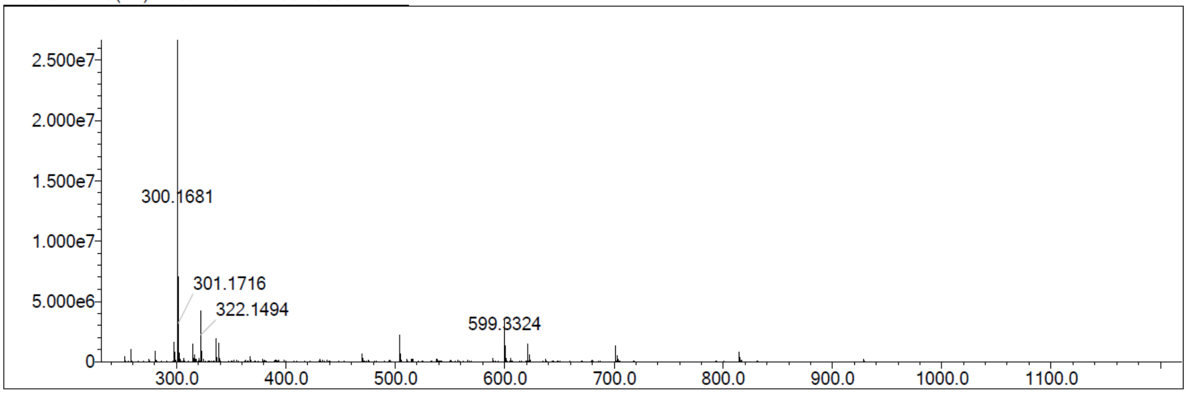

Measured region for 300.1706 m/z

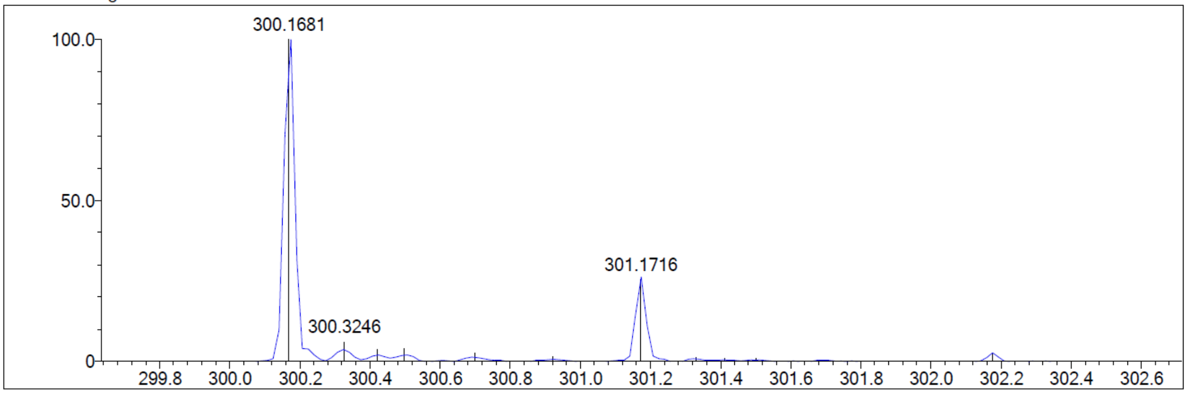

C17 H21 N3 O2 [M+H]+ : Predicted region for 300.1707 m/z

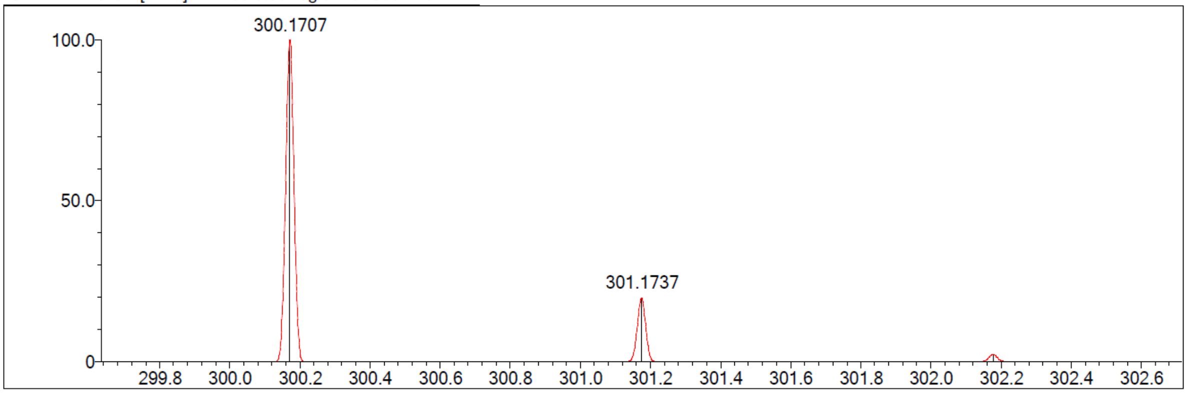

Rank	Score	Formula (M)	Ion	Meas. m/z	Pred. m/z	Df. (mDa)	Df. (ppm)	Iso	DBE
1	85.13	C17 H21 N3 O2	[M+H]+	300.1706	300.1707	-0.1	-0.33	85.13	9.0

N-(2-formylphenyl)butyramide (Inter4)

^{1}H NMR

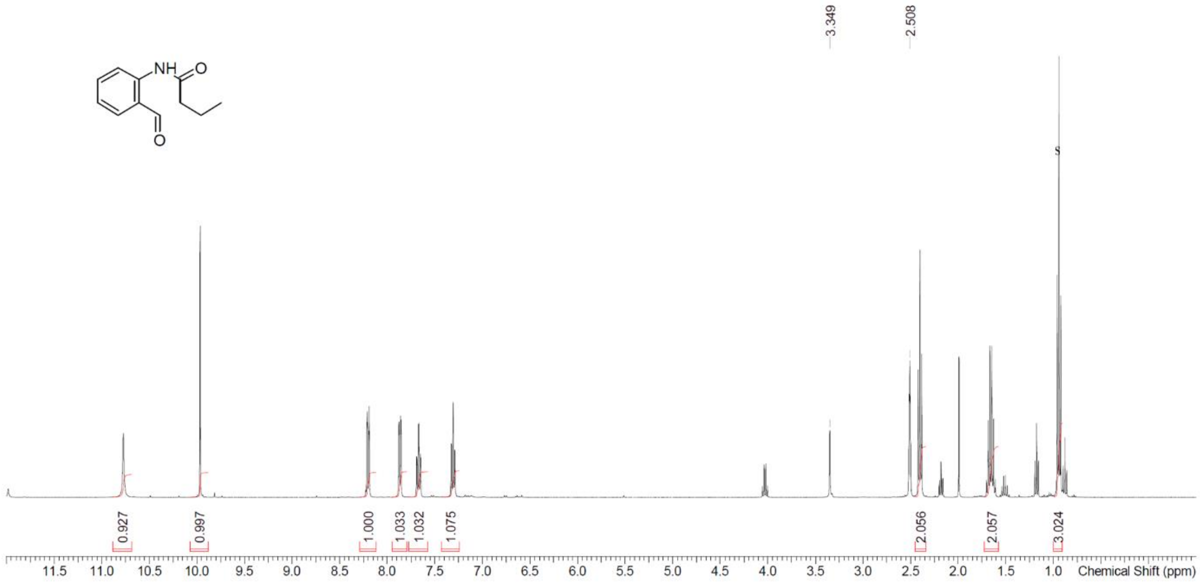

LC-MS

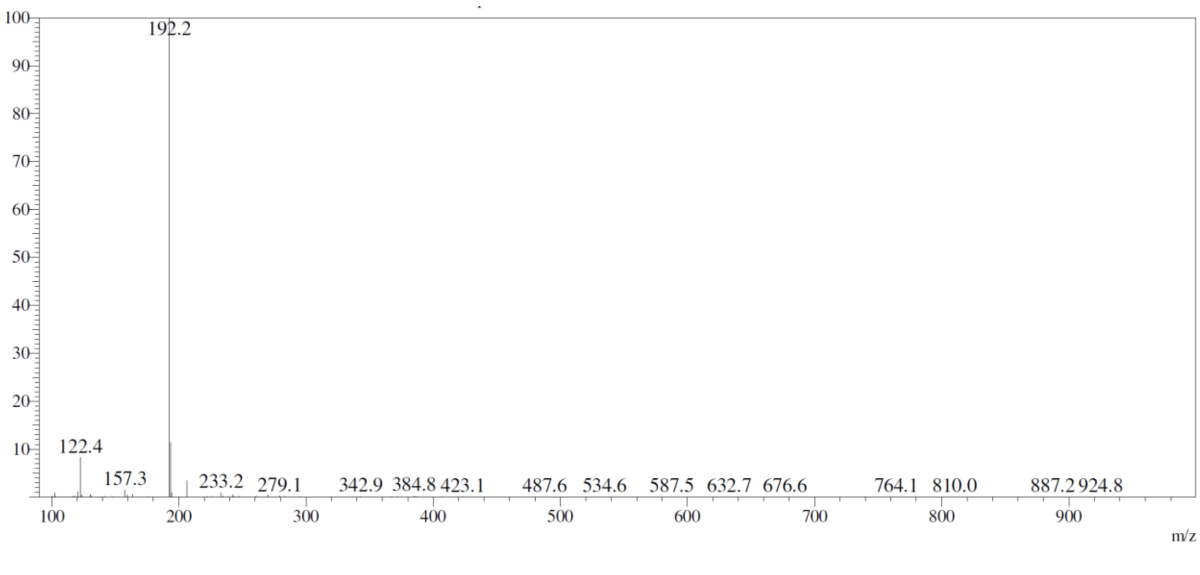

3-ethylquinolin-2(1H)-one (Inter5)

^{1}H NMR

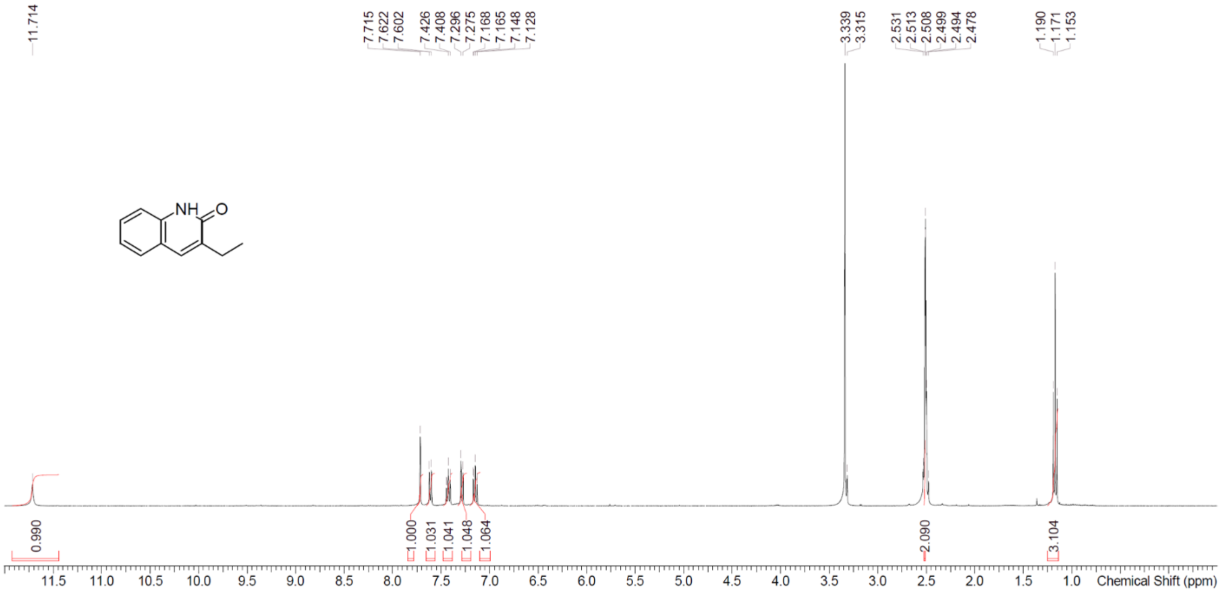

LC-MS

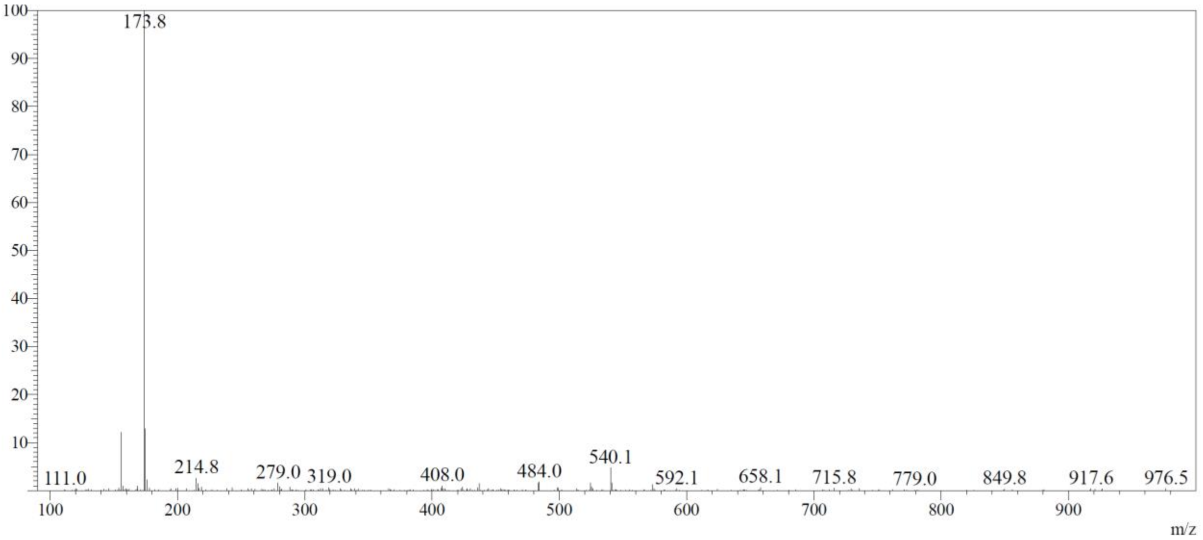

3-ethyl-6-nitroquinolin-2(1H)-one (Inter6)

^{1}H NMR

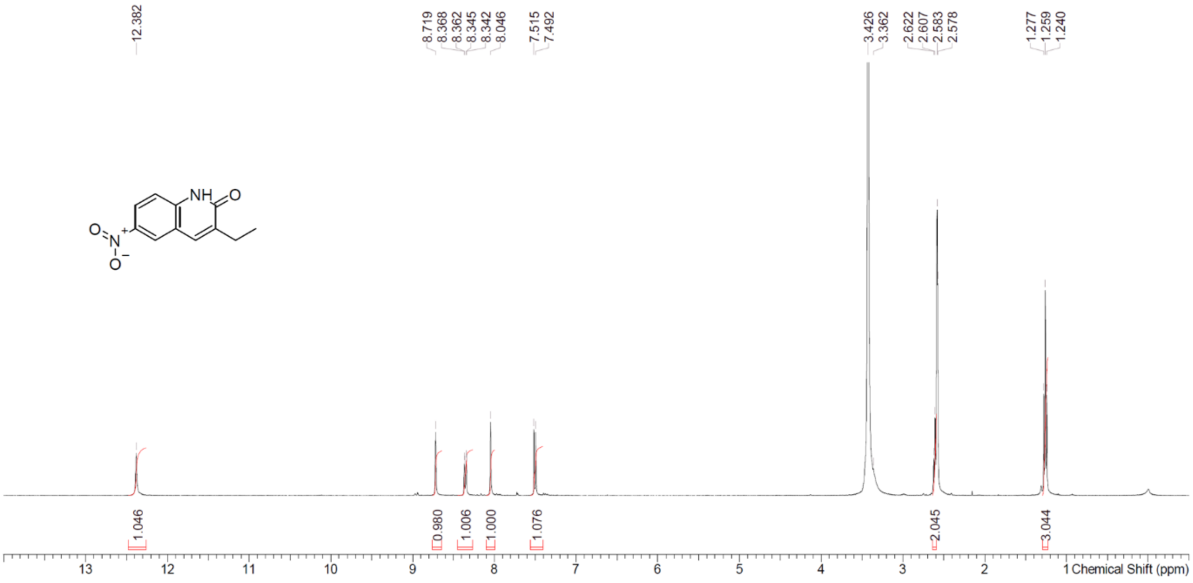

LC-MS

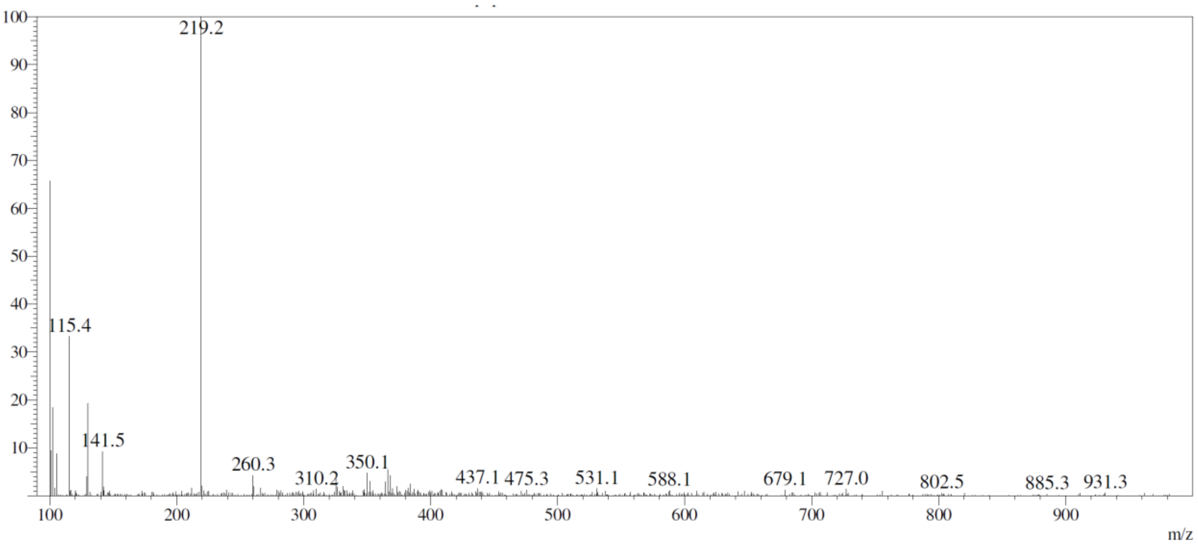

6-amino-3-ethylquinolin-2(1H)-one (Inter7)

^{1}H NMR

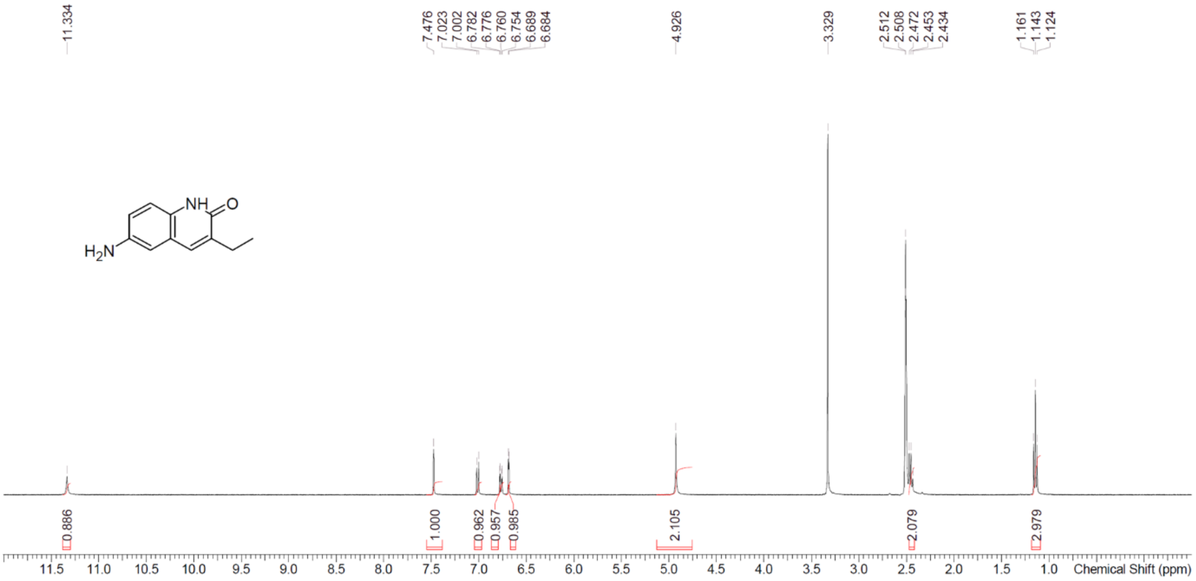

LC-MS

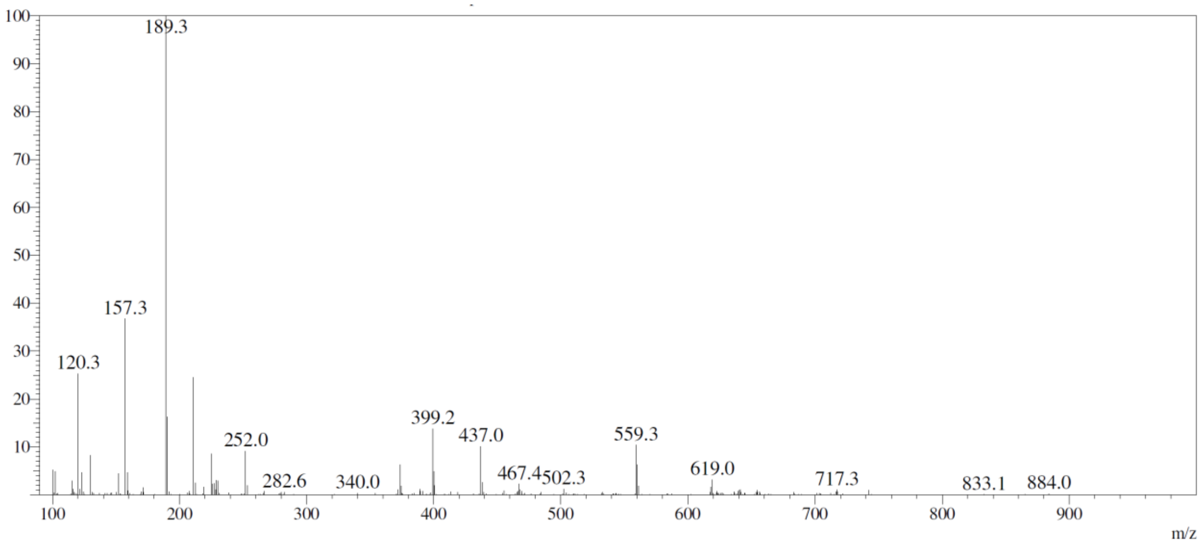

N-(3-ethyl-2-oxo-1,2-dihydroquinolin-6-yl)-4-fluoro-3-methylbenzenesulfonamide (HF4)

^{1}H NMR

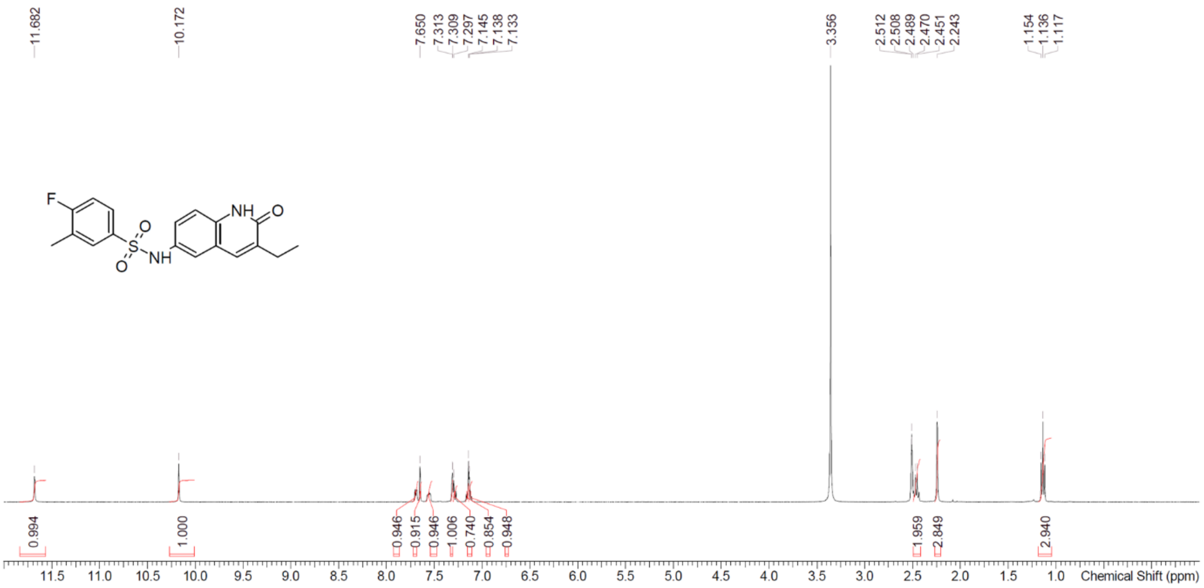

^{13}C NMR

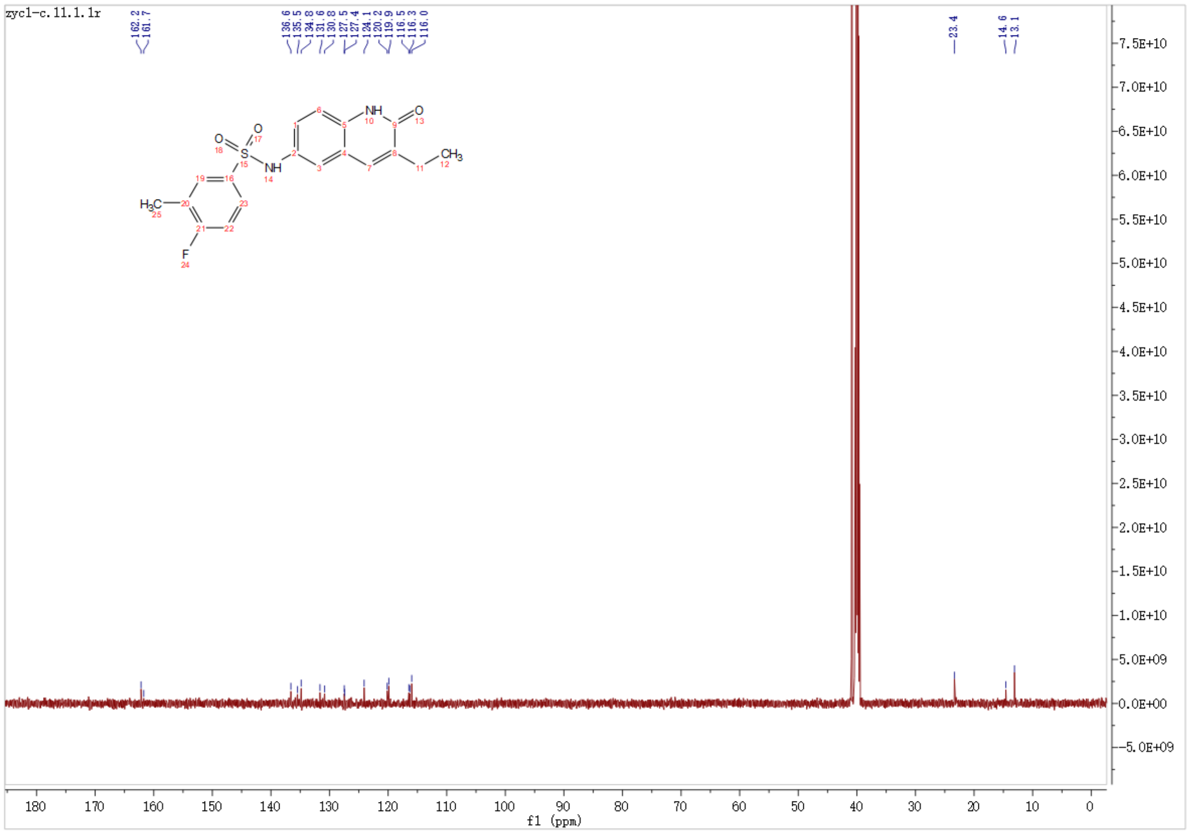

LC-MS

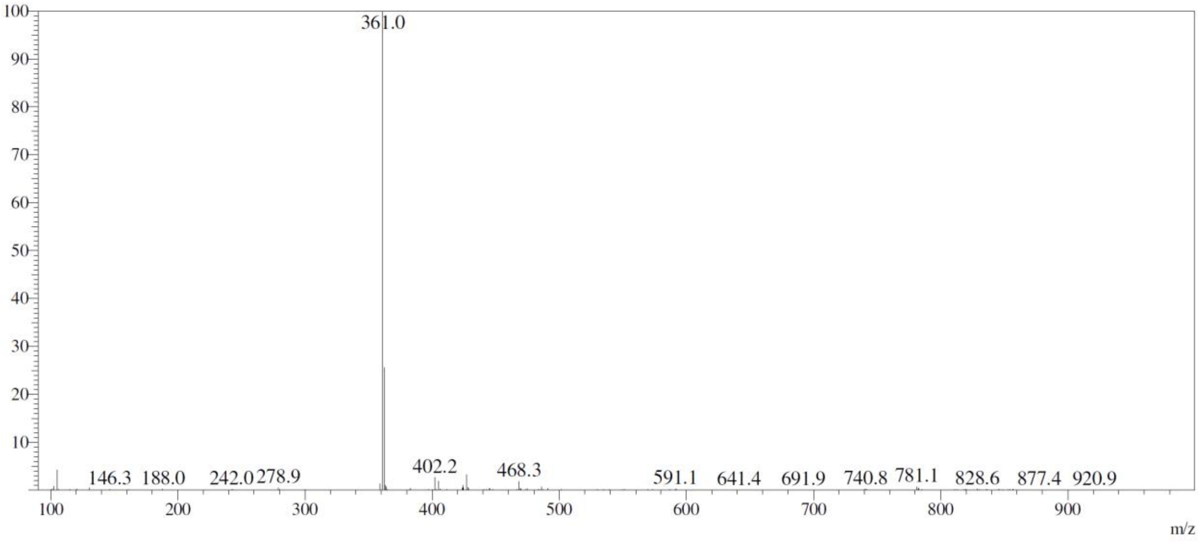

HPLC

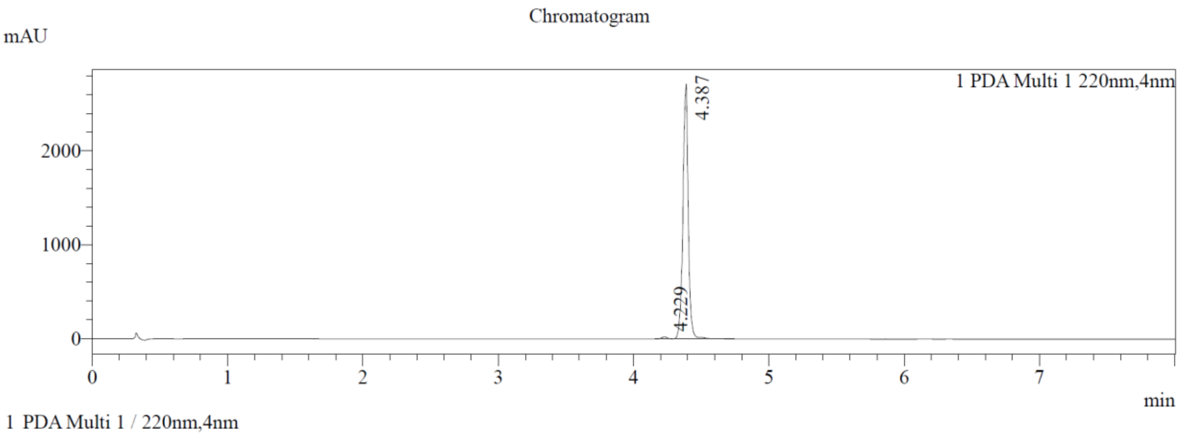

Integration Result

Peak Table

PDA Ch1 220nm

Peak#	Ret. Time	Height	Height%	USP Width	Area	Area%
1	4.229	23130	0.861	0.067	60967	0.817
2	4.387	2661825	99.139	0.066	7401365	99.183

HRMS

Elmt	Val.	Min	Max	Elmt	Val.	Min	Max	Elmt	Val.	Min	Max	Elmt	Val.	Min	Max	Use Adduct
H	1	17	17	O	2	2	4	P	3	0	0	I	3	0	0	H
2H	1	0	0	18O	2	0	0	S	2	0	1					Na
C	4	18	18	F	1	0	1	Cl	1	0	0					
N	3	2	3	Si	4	0	0	Br	1	0	0					

Error Margin (ppm):	5000	DBE Range:	not fixed	Electron Ions:	both
HC Ratio:	unlimited	Apply N Rule:	yes	Use MSn Info:	yes
Max Isotopes:	all	Isotope RI (%):	1.00	Isotope Res:	10000
MSn Iso RI (%):	75.00	MSn Logic Mode:	AND	Max Results:	23

Event#: 1 MS(E+) Ret. Time : 1.027 Scan# : 155

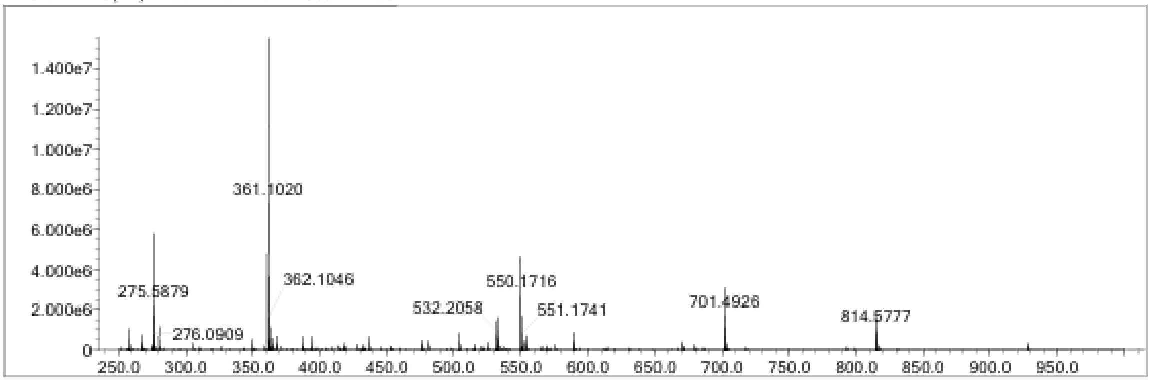

Measured region for 361.1020 m/z

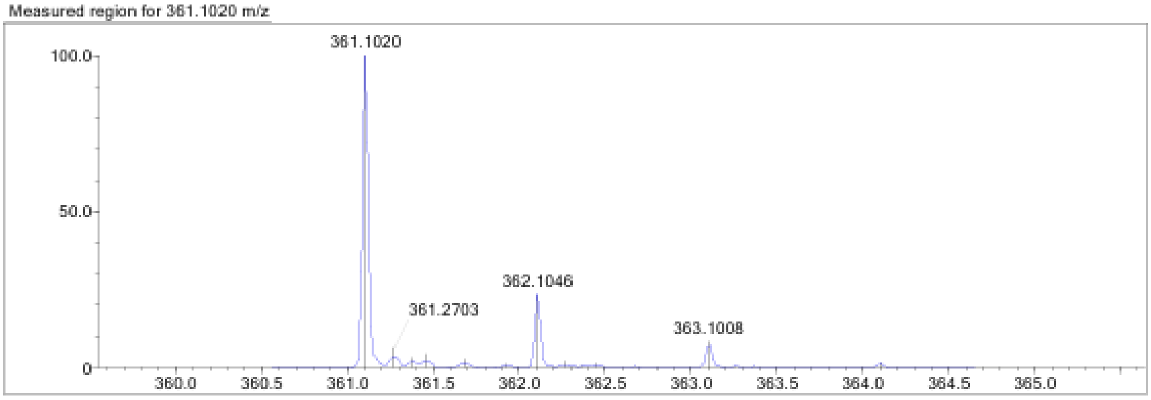

C18 H17 N2 O3 F S [M+H]+ : Predicted region for 361.1017 m/z

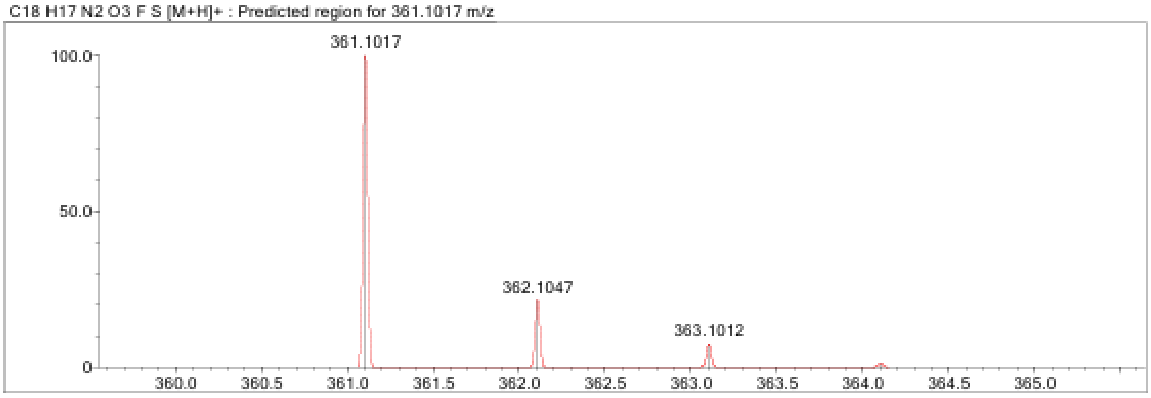

Rank	Score	Formula (M)	Ion	Meas. m/z	Pred. m/z	Df. (mDa)	Df. (ppm)	Iso	DBE
1	79.75	C18 H17 N2 O3 F S	[M+H]+	361.1020	361.1017	0.3	0.83	79.75	11.0

2-(dimethylamino)-N-(3-ethyl-2-oxo-1,2-dihydroquinolin-6-yl)acetamide (HF5)

^{1}H NMR

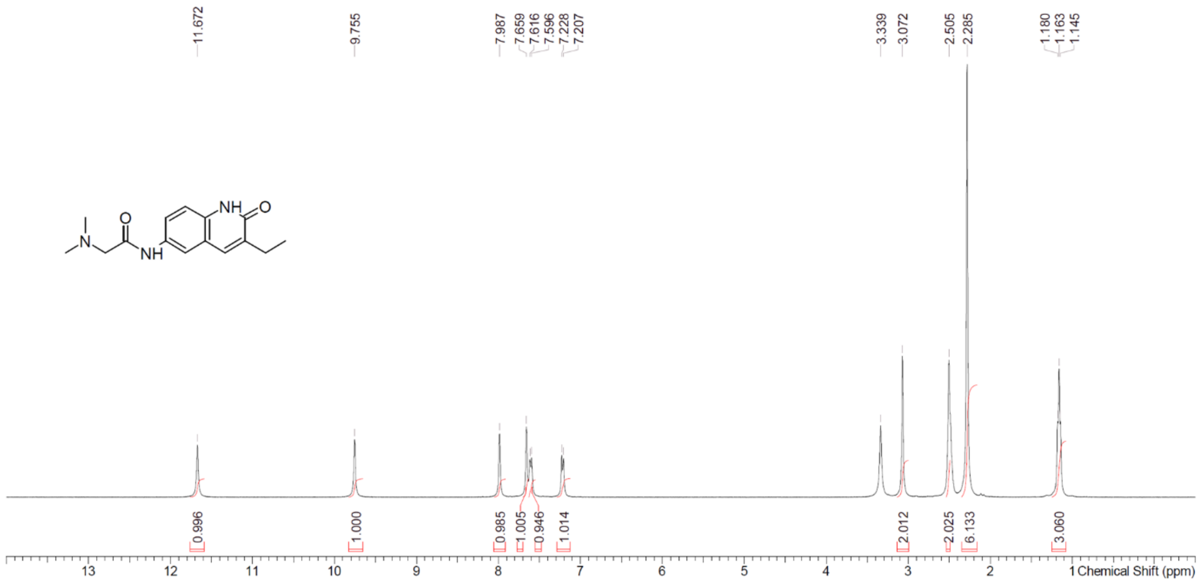

^{13}C NMR

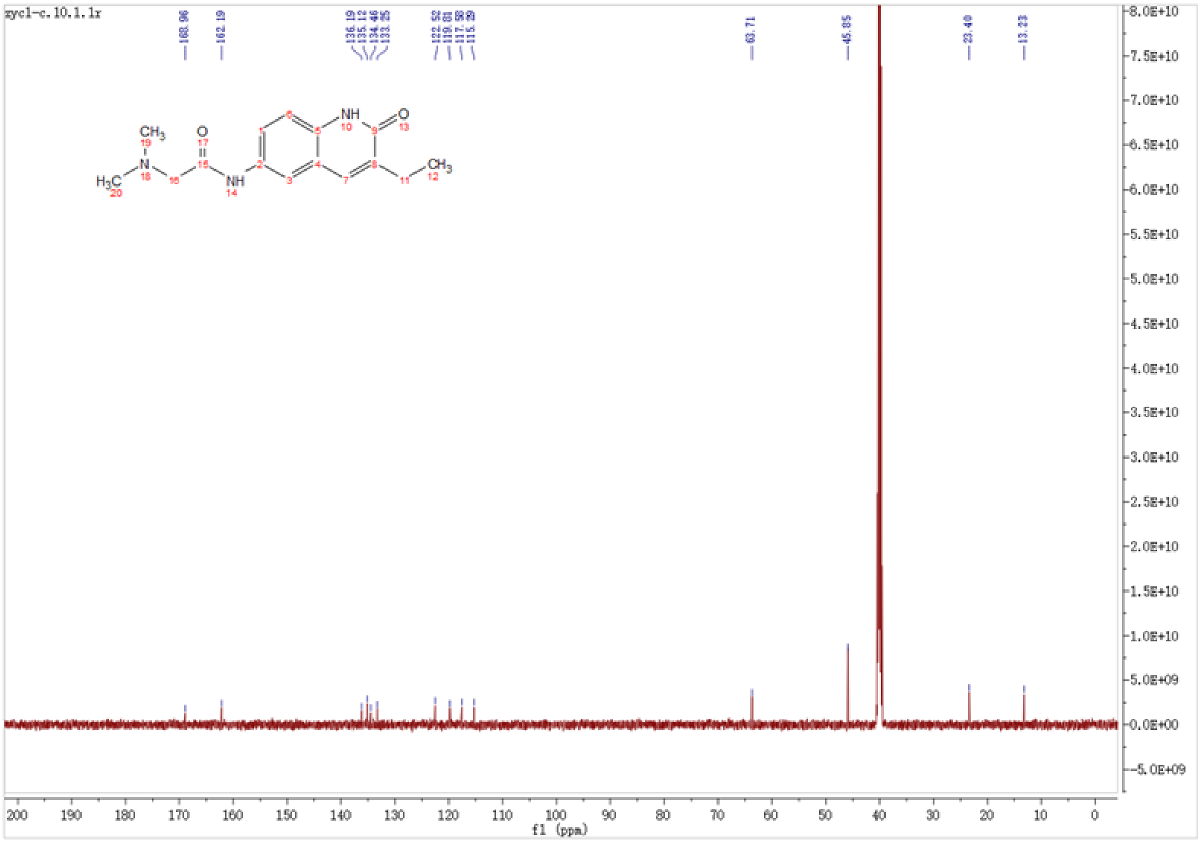

LC-MS

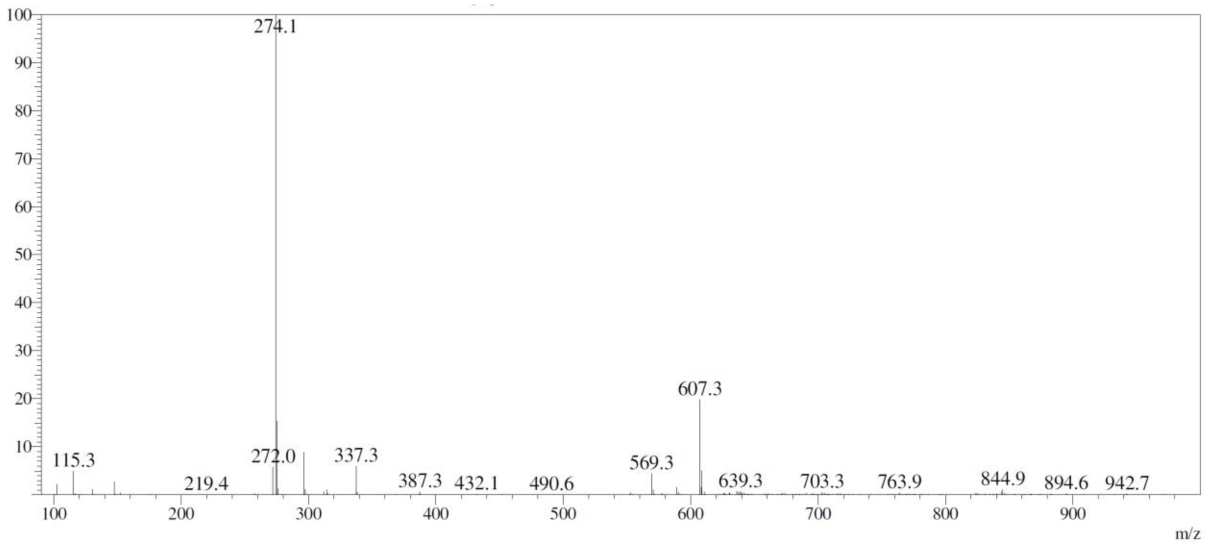

HPLC

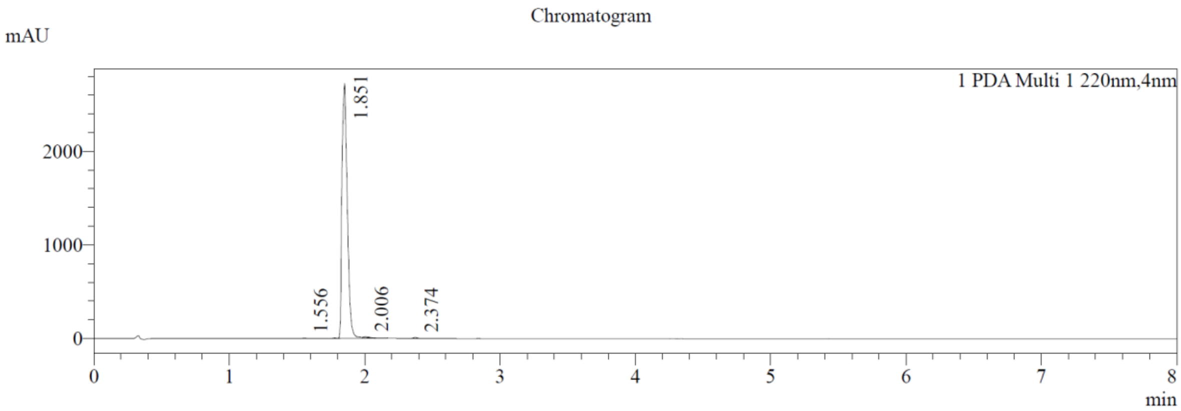

1 PDA Multi 1 / 220nm,4nm

==

Integration Result

==

Peak Table

PDA Ch1 220nm

Peak#	Ret. Time	Height	Height%	USP Width	Area	Area%
1	1.556	2838	0.106	0.058	6922	0.092
2	1.851	2667565	99.197	0.071	7512699	99.374
3	2.006	8103	0.301	0.070	18796	0.249
4	2.374	10643	0.396	0.051	21588	0.286

HRMS

Elmt	Val.	Min	Max	Elmt	Val.	Min	Max	Elmt	Val.	Min	Max	Elmt	Val.	Min	Max	Use Adduct
H	1	19	19	O	2	2	4	P	3	0	0	I	3	0	0	H
2H	1	0	0	18O	2	0	0	S	2	0	1					Na
C	4	15	15	F	1	0	1	Cl	1	0	0					
N	3	2	3	Si	4	0	0	Br	1	0	0					

Error Margin (ppm):	5000	DBE Range:	not fixed	Electron Ions:	both
HC Ratio:	unlimited	Apply N Rule:	yes	Use MSn Info:	yes
Max Isotopes:	all	Isotope RI (%):	1.00	Isotope Res:	10000
MSn Iso RI (%):	75.00	MSn Logic Mode:	AND	Max Results:	23

Event#: 1 MS(E+) Ret. Time : 1.293 Scan# : 195

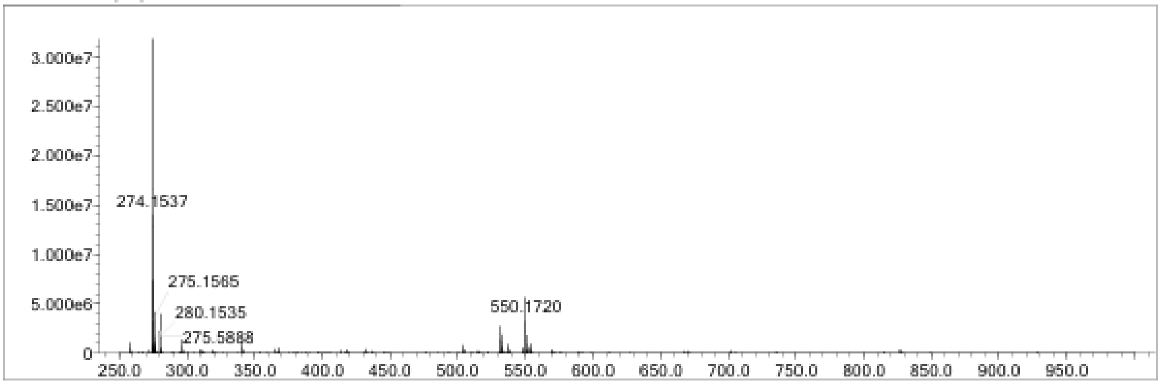

Measured region for 274.1537 m/z

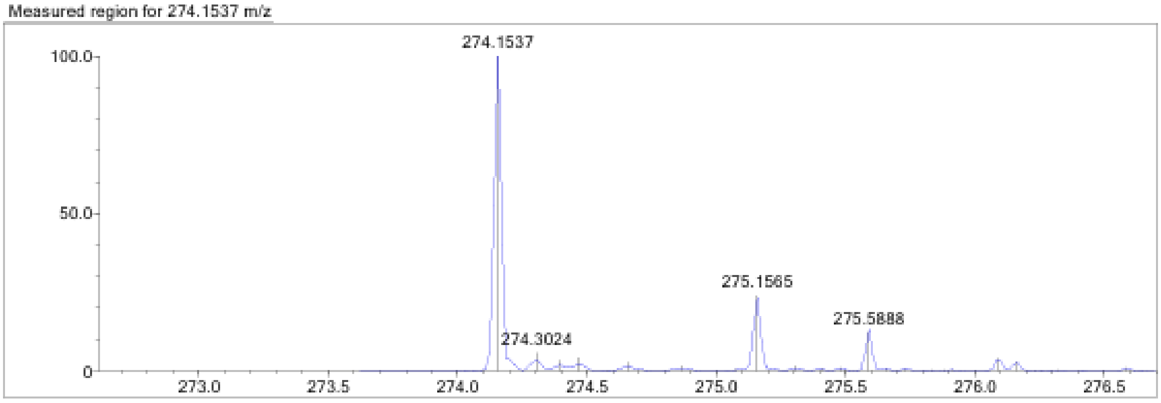

C15 H19 N3 O2 [M+H]+ : Predicted region for 274.1550 m/z

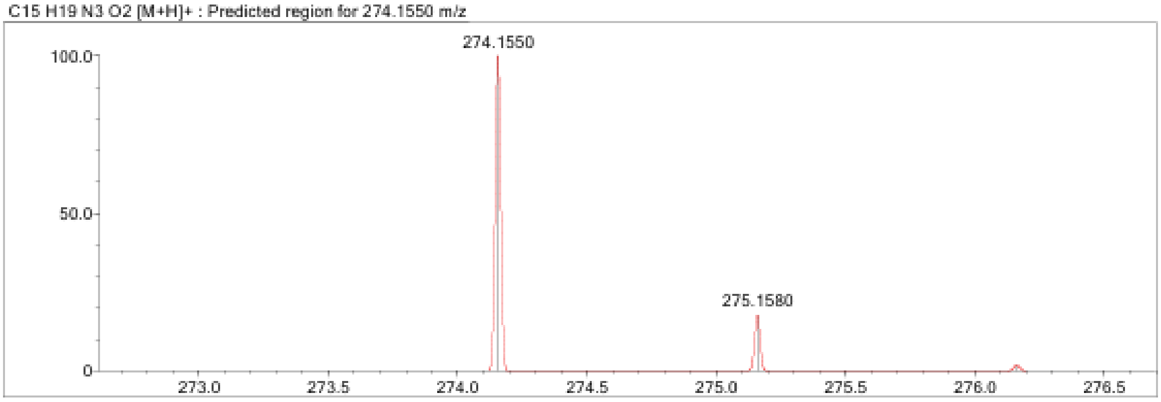

Rank	Score	Formula (M)	Ion	Meas. m/z	Pred. m/z	Df. (mDa)	Df. (ppm)	Iso	DBE
1	82.05	C15 H19 N3 O2	[M+H]+	274.1537	274.1550	-1.3	-4.74	90.51	8.0
